# The Antimicrobial Peptide Capitellacin: Chemical Synthesis of Analogues to Probe the Role of Disulphide Bridges and Their Replacement with Vinyl Sulphides

**DOI:** 10.3390/antibiotics13070615

**Published:** 2024-07-02

**Authors:** Oscar A. Shepperson, Paul W. R. Harris, Margaret A. Brimble, Alan J. Cameron

**Affiliations:** 1 School of Chemical Sciences, The University of Auckland, 23 Symonds St., Auckland 1010, New Zealand; 2 School of Biological Sciences, The University of Auckland, 3A Symonds St., Auckland 1010, New Zealand; 3 Maurice Wilkins Centre for Molecular Bio-Discovery, The University of Auckland, 3A Symonds St., Auckland 1010, New Zealand

**Keywords:** β-hairpin, capitellacin, vinyl sulphide, AMP, allenamide

## Abstract

Capitellacin (**1**) is a 20-residue antimicrobial β-hairpin, produced by the marine polychaeta (segmented worms) *Capitella teletai*. Since its discovery in 2020, only very limited studies have been undertaken to understand capitellacin’s structure–activity relationship (SAR). Using fast-flow Fmoc-SPPS, a focused library of capitellacin analogues was prepared to systematically study the influence of the two disulphide bridges on its structure and activity, and their replacement with a vinyl sulphide as a potential bioisostere. Upon studying the resulting peptides’ antimicrobial activity and secondary structure, the most terminal disulphide emerged as the most critical element for maintaining both bioactivity and the secondary structure, properties which were demonstrated to be closely interlinked. The removal of the innermost disulphide bridge or disulphide replacement with a vinyl sulphide emerged as strategies with which to tune the activity spectrum, producing selectivity towards *E. coli*. Additionally, an enantiomeric d-capitellacin analogue revealed mechanistic insights, suggesting that chirality may be an inherent property of capitellacin’s bacterial membrane target, or that a hitherto unknown secondary mechanism of action may exist. Additionally, we propose the Alloc protecting group as a more appropriate alternative to the common Dde group during fast-flow Fmoc-SPPS, in particular for short-chain diamino acids.

## 1. Introduction

As the threat of antimicrobial resistance (AMR) increases and multi-drug-resistant (MDR) strains of bacteria continue to emerge in hospitals and communities [1,2], medicinal chemists have focused on the development of novel antimicrobial chemotherapeutic agents. Ideally, these should possess novel mechanisms of action to reduce the incidence of resistance observed within current MDR pathogens [3]. A key source for the discovery of such chemotherapeutics remains ‘Mother Nature’, with antimicrobial peptides (AMPs) constituting an exciting field of interest [4].

**Figure 1:**
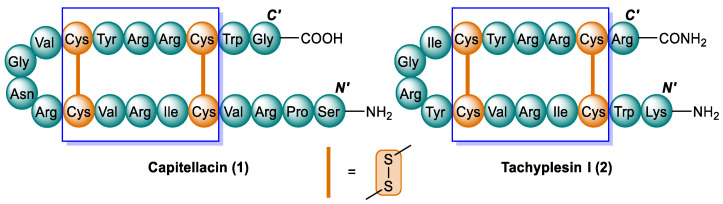
Comparison of sequences of capitellacin (**1**) and tachyplesin I (**2**). Areas of sequence homology are highlighted by boxes (blue), and Cys residues and disulphides are in orange.

AMPs are a universal defence mechanism, preserved throughout evolution in a diverse variety of species [4]. These short peptides (<50 AAs) form part of the innate immune system, the first line of defence for all complex life [4]. Despite a small number being FDA-approved, AMP applications are often limited due to concerns regarding toxicity [5]. Marine invertebrates are among the organisms most highly reliant on AMPs, with their lack of acquired immunity leaving them otherwise defenceless to their harsh and bacterial-laden environments [6,7]. When compared to their global abundance, marine invertebrates remain largely unexplored. However, the minimal number of species investigated to date have provided promising broad-spectrum AMP leads [8,9,10,11]. Recently (2020), a 20-residue peptide, capitellacin (**1**), produced by the marine polychaeta (segmented worms) *Capitella teleta*, was discovered through recombinant expression [12], garnering significant interest due to its sequence homology to the extensively studied and potent β-hairpin AMP, tachyplesin I (**2**) [12,13] (Figure 1).

**Figure 2:**
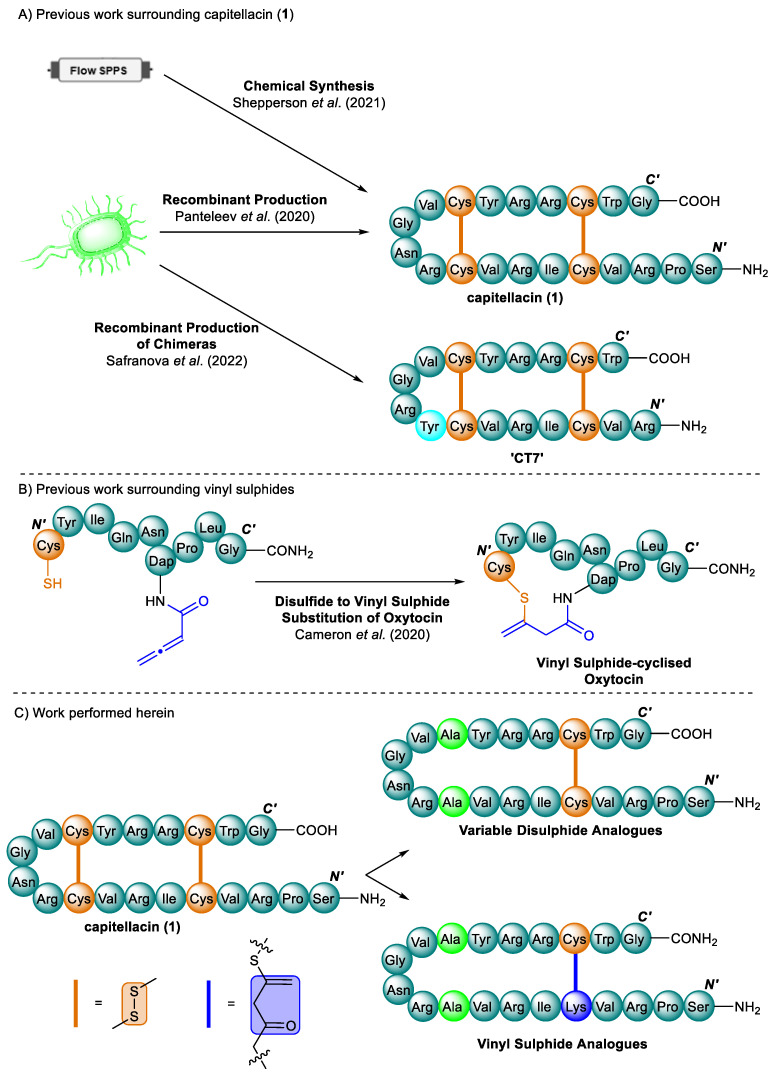
(**A**) Previous work surrounding the synthesis of capitellacin (**1**) and tachyplesin (**2**) chimeras (bright blue), recombinantly and synthetically produced [12,15,16]. (**B**) Previous work, whereby vinyl sulphide was incorporated as a disulphide mimetic [27], and (**C**) work performed herein, showing a single representative disulphide and vinyl sulphide analogue. Cys residues: orange; Ala residues: bright green; and substituted Cys residue and vinyl sulphide bridge: blue.

Nuclear magnetic resonance (NMR) spectroscopy has revealed that the solution structure of capitellacin (**1**) exists as a monomeric antiparallel β-hairpin (type IV β-turn), stabilised by a pair of parallel disulphide bonds (Cys^5^-Cys^18^ and Cys^9^-Cys^14^) [12]. Further work has shown that capitellacin (**1**) exhibits broad-spectrum activity and, uncharacteristically (for known β-hairpins), minimal lysis of human red blood cells (hRBCs), resulting in a class-leading therapeutic index [12,14]. Subsequently, our group pursued the total chemical synthesis of capitellacin (**1**), further validating its chemical structure and activity towards Gram-negative pathogens, although the previously reported activity towards *Staphylococcus aureus* could not be reproduced [15]. Most recently, Ovchinnikova and co-workers (2022 and 2024) have continued their work, following the initial expression of capitellacin (**1**), which expresses a series of chimeric analogues inspired by tachyplesin I (**2**) (Figure 2A). Alongside their investigation of the activity of these chimeras, they determined that capitellacin (**1**) acted via a ‘carpet’ or detergent-like mechanism, whereby the peptide accumulates on the surface of the bacterial membrane via a dimer, causing fluctuations in conductivity, which, at a certain threshold, result in complete membrane destruction [16,17]. Despite its structural similarity to tachyplesin I, which carries a net charge of +7 at physiological pH, capitellacin has a reduced net charge of +5, which may be at least in part responsible for its different activity profile, especially its reduced haemolytic potential.

Previous works surrounding β-hairpin AMPs have commonly involved the determination of their SAR, in particular, the effect of the removal or substitution of the disulphide bonds, with notable β-hairpins (e.g., tachyplesin I [2]) exhibiting an alternative mechanism of action (MoA) upon disulphide removal [18,19]. Additionally, the removal of one or more disulphide bonds has been shown to significantly reduce β-hairpin toxicity (haemolysis) by offering greater conformational flexibility [20]. Unfortunately, this often comes at the expense of moderately reduced antimicrobial potency, but ultimately may render β-hairpins more viable as potential clinical agents [20,21,22]. The most significant work reporting on disulphide removal from bicyclic β-hairpin AMPs has been performed on the arenicin 3 variant NZ17074 [22,23]. The authors coined the terms ‘kite’ and ‘bullet’ to refer to disulphide removal by alanine substitution at the terminal and innermost bridges, respectively [22,23]. Alongside disulphide removal, disulphide replacement has been investigated on the similar, bicyclic disulphide-bridged β-hairpins gomesin and tachyplesin I (**2**) [24,25]. Gomesin underwent singular disulphide replacement with lactam bridges of varying length [24], whereas tachyplesin I (**2**) underwent bis-disulphide replacement with triazoles [25]. In both cases, the activity of the β-hairpins was maintained to varying degrees, highlighting the applicability of alternative intramolecular cyclisation chemistries, such as bioisosteres or surrogates, for disulphide bridges in AMPs.

Vinyl sulphide, formed by the thia-Michael addition of a thiol to an allenamide under mild conditions (physiological pH and aqueous solvents), represents a minimally explored motif with the potential to act as a disulphide surrogate with tuneable lengths and stereochemistry. This moiety was initially developed for the chemoselective bioconjugation of thiol-containing peptides or proteins with allenamide-functionalised fluorescent labels [26]. In 2020, we expanded upon this approach by developing a methodology to incorporate allenamide functionality into a peptide on-resin by coupling 3-butynoic acid, with the culmination of this work resulting in a vinyl sulphide-cyclised oxytocin model peptide [27] (Figure 2B). The chemical substitution of disulphide bonds is an important strategy for improving the drug-like behaviour and metabolic stability of cysteine-rich peptides [28,29]. Disulphide bonds could otherwise undergo undesired cleavage or scrambling, due to enzymatic processes or with other thiol-containing biomolecules, such as serum albumin or glutathione. Unlike other highly electrophilic Michael acceptors, such as maleimides, which can readily undergo retro-Michael decomposition in the presence of biological reducing agents, vinyl sulphide has proved to be stable and unreactive to biologically relevant thiols, further warranting its exploration as a potential disulphide surrogate [26,30]. Furthermore, vinyl selenide conjugates formed through the seleno-Michael addition of Sec to an allenamide have demonstrated excellent plasma stability, and are isosteric with the more readily accessible vinyl sulphides [31].

Inspired by the wide range of work investigating the SAR of other β-hairpins, we set out to create a library of analogues of capitellacin (**1**), to probe the removal and replacement of the two disulphide bridges. These analogues were studied to determine the minimal requirements to maintain bioactivity, and the relationship this has with peptide secondary structure. Furthermore, we sought to investigate our intramolecular vinyl sulphide cyclisation approach as a disulphide surrogate, whereby the effect of the vinyl sulphide direction, length, and stereochemistry was examined to determine its applicability as a disulphide bioisostere (Figure 2C). For the synthesis of all our analogues, our previous approach to the first chemical synthesis of capitellacin (**1**) was employed with a minor modification, whereby fast-flow solid-phase peptide synthesis (SPPS) was implemented with a high degree of efficiency [15].

## 2. Results and Discussion

### 2.1. Disulphide Library Design

**Figure 3:**
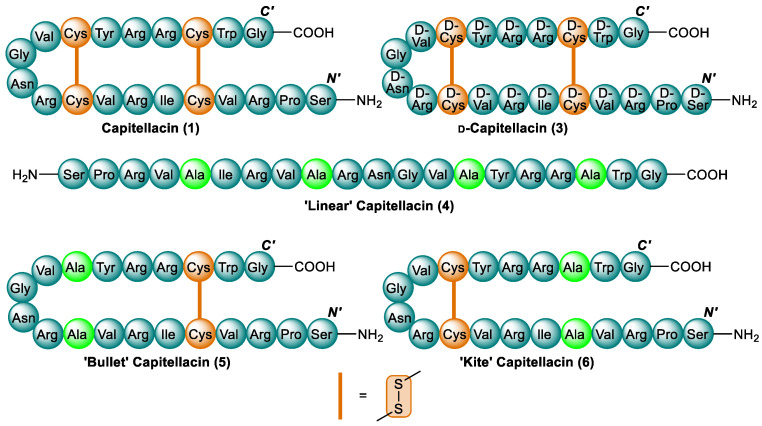
Capitellacin analogue library: d-analogue (**3**), linear analogue (**4**), and disulphide analogues (**5**–**6**). Cys residues and disulphides are in orange and Ala residues are in bright green.

The disulphide analogue series was designed to incorporate the native capitellacin (**1**) peptide alongside its d-enantiomer (**3**) and three analogues with varying degrees of disulphide intramolecular cyclisation or no disulphide (Figure 3). The varying degrees of disulphide cyclisation were similar to the work performed on the arenicin analogue NZ17074 [22], with ‘linear’ (**4**), ‘bullet’ (**5**), and ‘kite’ (**6**) systematic naming employed (Figure 3). Accordingly, this enabled the examination of the impact of each disulphide bond upon the bioactivity and solution structure, aiming to determine the minimum requirements for the maintenance of these key properties. d-enantiomer (**3**) was synthesised to probe if capitellacin acts as an antimicrobial by binding to a chiral molecular target. Any significant loss in activity due to an l- to d-enantiomeric switch often indicates a more specific protein–protein interaction, such as the MoA of the AMP, as has been previously reported for the β-hairpin thanatin [32,33]. Although the mechanism of action of native capitellacin (**1**) was recently studied [16], d-enantiomer was not examined, nor was an exact molecular target identified. A significant resultant change in the activity of d-enantiomer may suggest that capitellacin (**1**) elicits its antimicrobial activity via a more complex interaction than that previously reported [16]. Alternatively, a decrease in activity may also support the speculation that intrinsic aspects of microbial membranes may exhibit a degree of ‘handedness’, and that membrane interactions can be stereospecific [34].

### 2.2. Synthesis of Linear and Disulphide Analogues **3**–**6**

As we have previously standardised a protocol for the synthesis of native capitellacin (**1**) by fast-flow Fmoc-SPPS and on-resin orthogonal disulphide formation [15], these conditions were employed for the synthesis of analogues **3**, **5**, and **6**, as well as congener **4**, devoid of Cys residues. As multiple disulphide bonds were required for **3**, a cysteine-protecting strategy (acetamidomethyl/trityl [Acm/Trt]) analogous to our prior work was implemented [15]. Where single disulphide bonds were present (**5** and **6**), Cys (Trt) was chosen for synthesis, and iodine (I_2_) in 1,4-dioxane (dioxane) was used to effect the oxidative deprotection [15,35].

The linear peptides were assembled on-resin by fast-flow Fmoc-SPPS in accordance with our previous work, on a ca. 0.15 scale (TentaGel^®^-S-NH_2_, 0.25 mmol/g resin substitution). Resin functionalisation (Wang-like linker, HMPA [4-hydroxymethyl]phenylacetic acid) and peptide elongation were performed as previously reported, with non-cysteine amino acids employing 1-[bis(dimethylamino)methylene]-1*H*-1,2,3-triazole [4,5-b]pyridinium 3-oxide hexafluorophosphate (HATU) and *N*,*N*′-diisopropylethylamine (DIPEA), and cysteine residues employing (7-azabenzotriazol-1-yloxy)trispyrrolidinophosphonium (PyAOP) and 2,4,6-trimethylpyridine (*sym*-collidine). Following linear peptide elongation, the linear precursors of **1**, **3**, **5**, and **6** underwent oxidative deprotection with I_2_ as required, in the respective solvent for the protecting groups present [35]. The resultant final peptides were liberated from their respective resins using trifluoroacetic acid (TFA)/triisopropylsilane (TIPS)/Milli-Q water (MQ H_2_O) (95:2.5:2.5, *v*/*v*/*v*, 3 h, r.t.) to yield the desired peptides **1**, **3**–**6**. Following trituration with diethyl ether (Et_2_O), centrifugation, re-solubilisation, and lyophilisation, the desired crude peptides were obtained in moderate yields (25–60% [based on initial resin loading]). The crude peptides (**1**, **3**–**6**) were purified by semi-preparative, reverse-phase, high-performance liquid chromatography (RP-HPLC) to obtain the final isolated products of high purity (>95%) in moderate yields (Supplementary Table S1, as confirmed by RP-HPLC and high-resolution mass spectrometry (HRMS) (Supplementary Figures S1–S5, S12–S13 and S22–S26).

### 2.3. Biological Investigation of Analogues **1**, **3**–**6**

Capitellacin (**1**) and analogues **3**–**6** were tested for their antimicrobial activity towards selected pathogens (Table 1), including *Pseudomonas aeruginosa*, *Escherichia coli,* and *S. aureus*. Furthermore, the peptides were tested for their activity towards the fungus *Candida albicans*, which has not been previously investigated. For the antimicrobial testing, polymyxin B (PMB) and amoxicillin (AMX) were used as the controls for Gram-negative and Gram-positive bacteria, respectively, and amphotericin B (AMB) was employed for the fungi (Table 1).

Analogues **3**–**6** showed variable activity with respect to native capitellacin (**1**), providing insight into the role of each disulphide bond (Table 1). None of the peptides tested (**1** and **3**–**6**) demonstrated inhibitory activity towards the tested strain of *S. aureus* (ATCC25923), and only **1** demonstrated modest activity towards *C. albicans* (SC5314), with an MIC of 64 μM. The lack of activity towards *S. aureus* is in accordance with our prior report on synthetic capitellacin (**1**); however, it is noteworthy that different bacterial strains were used in the testing of the recombinant peptide [15]. Increasing the flexibility of the molecule by removing the disulphide bridges in compounds **3**–**6** did not alter the spectrum of activity, and failed to produce activity towards the tested Gram-positive pathogen or fungus. However, varying degrees of activity were observed towards *E. coli* and *P. aeruginosa* for peptides **1** and **3**–**6**. The removal of both disulphide bonds in **4** completely abolished any activity. Furthermore, we found that a single disulphide in the outermost position (‘bullet’ analogue, **5**) was sufficient to retain a potency towards *E. coli* equivalent to that of capitellacin (**1**). However, a 4-fold reduction in activity towards *E. coli* was observed for the ‘kite’ analogue (**6**). Interestingly, while retaining potency towards *E. coli*, a 4-fold reduction in potency towards *P. aeruginosa* was observed for the ‘bullet’ analogue. This suggests that the ‘bullet’ structural simplification is well tolerated if the aim is to offer a more selective antimicrobial compound, and may also result in a change in mechanism or target binding. Cooper and co-workers found that the removal of either disulphide bridge from tachyplesin I also reduced activity, although in some cases only modestly, depending on the choice of residue substation (e.g., Cys for Ala or Ser) [36]. Others have made similar observations for tachyplesin I and arenacin 3 [20,22]. Of further interest, the d-enantiomer of capitellacin (**3**) exhibited a 2–4-fold reduction in activity towards the Gram-negative strains tested compared to synthetic capitellacin (**1**). As previously eluded to, such a result suggests that capitellacin (**1**) may exert its antimicrobial activity in a more complex fashion than previously described [16]. Finally, for the peptides examined, a positive correlation was observed between hydrophobicity (as judged by an analytical RP-HPLC) and activity towards *E. coli* (Supplementary Table S2), with the exception of d-enantiomer (**3**), which had an identical retention time as that of l-enantiomer (**1**).

### 2.4. Circular Dichroism Investigation of Analogues **1**, **3**–**6**

**Figure 4:**
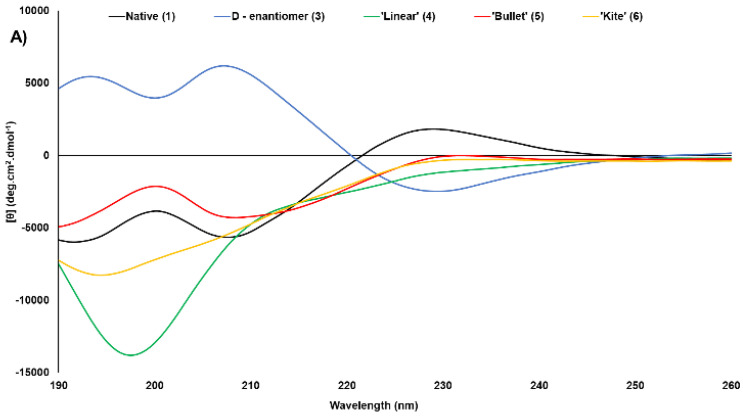

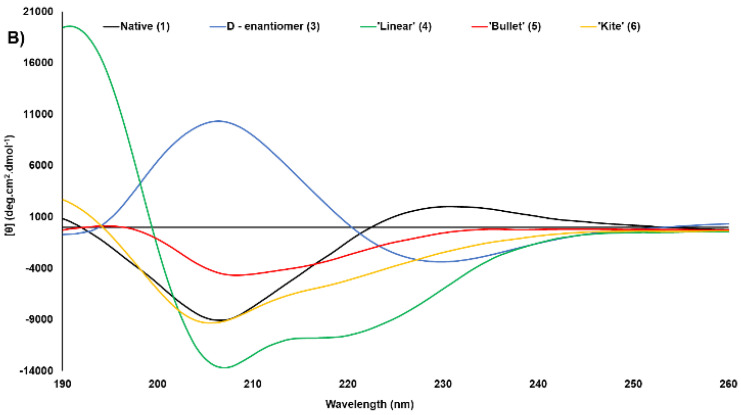
(**A**) CD spectra overlay of capitellacin (**1**) and analogues **3**–**6** at 50 µM in 200 µM sodium phosphate buffer (pH ~7.4, 20 °C). (**B**) CD spectra overlay of capitellacin (**1**) and analogues **3**–**6** at 50 µM in TFE:200 µM sodium phosphate buffer 1:1 (pH ~7.4, 20 °C).

To determine the role of each disulphide in maintaining the secondary structure of capitellacin (**1**), we examined **1** alongside analogues **3**–**6** by circular dichroism (CD). The spectra were collected for two solvent systems, a sodium phosphate buffer (pH ~7.4, 200 µM) and a sodium phosphate buffer/trifluoroethanol (TFE) (1:1, *v*/*v*, pH ~7.4, 200 µM) (Figure 4). β-hairpin peptides often do not exhibit strong β-sheet characteristics in CD, as their β-sheet region contributes a relatively small proportion of ellipticity compared to that of the entire peptide [37,38]. As the secondary structure was previously defined as a β-hairpin by NMR [12], the expected lack of the characteristic β-sheet maxima/minima in CD was of no concern; instead, the CD spectrum of the native peptide (**1**) was treated as a ‘fingerprint’ for a comparative qualitative analysis.

Upon an examination of native capitellacin (**1**) and d-enantiomer (**3**), both peptides exhibited highly similar spectra in the sodium phosphate buffer (pH ~7.4), noting that enantiomers form mirror images of one another along y = 0 when examined at the same concentration (Figure 4A and Supplementary Figure S33). Pleasingly, the ‘bullet’ analogue (**5**) also exhibited a highly similar spectrum despite the removal of one disulphide bridge (Figure 4A and Supplementary Figure S33), further suggesting capitellacin (**1**) may be amenable to simplification. Comparatively, the ‘linear’ and ‘kite’ analogues (**4** and **6**) exhibited significantly different spectra to that of native capitellacin (**1**) (Figure 4A and Supplementary Figure S34). Peptides **4** and **6** likely adopted a much greater degree of random coil structure, as evidenced by the large negative maxima in the 190–200 nm region. The spectra recorded for the secondary structure-favouring environment (sodium phosphate buffer/TFE, 1:1, *v*/*v*) [39], revealed analogues **4**–**6** to be far more similar (Figure 4B). A consistent negative maxima (positive for d-enantiomer) was observed at ~208 nm, suggesting the better maintenance of the secondary structure in this solvent system. A point of interest on the CD spectra was the ‘linear’ analogue (**4**), which in the secondary structure-favouring solvent system exhibited a spectrum typical of an α-helical structure [40,41], with negative maxima at ~208 nm and ~222 nm and a strong positive maxima at ~190 nm (Figure 4B and Supplementary Figure S35). A possible reasoning for this may be due to the increased flexibility of the linear analogue (**4**). This substantial change in secondary structure would additionally explain its total loss of antimicrobial activity (Table 1). Overall, the CD spectra established that the disulphide bond between Cys^5^ and Cys^18^, which yields the ‘bullet’ analogue (**5**), has the most substantial influence on the secondary structure and ensuing antimicrobial activity of capitellacin (**1**). For further investigations of the peptidyl structure, including the substitution of the disulphide bonds, these results present analogue **5** as a great starting point, since its structure (and activity, Table 1) is not largely varied but the overall complexity of the peptide is reduced.

### 2.5. Vinyl Sulphide Library Design

**Figure 5:**
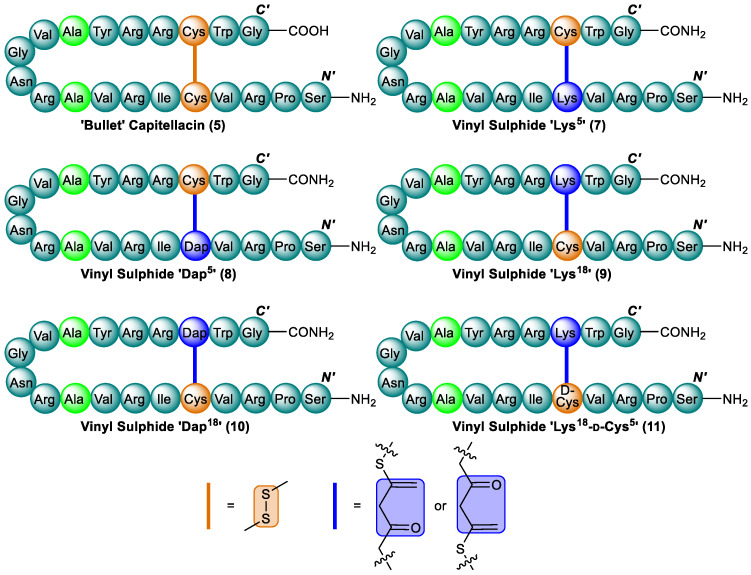
Capitellacin vinyl sulphide analogue library. ‘Bullet’ analogue (**5**) and vinyl sulphide analogues (**7**–**11**). Cys residues are in orange, Ala residues are in bright green, and substituted Cys residues and vinyl sulphide bridge are in blue.

With further SAR knowledge in-hand, we designed a series of vinyl sulphide analogues derived from ‘bullet’ capitellacin (**5**) (Figure 5). The vinyl sulphide analogues were all modified with a *C*-terminal carboxamide to improve the overall cationic character, as is commonplace in peptide development [42]. A *C*-terminal carboxamide has rarely been shown to affect peptide structure, but offers the advantage of increasing the overall positive charge at a physiological pH, promoting the desired membrane adhesion of cationic AMPs [43,44]. Additionally, the Rink-amide linker, which releases a carboxamide upon cleavage, offers a more reliable linkage strategy than ester-based linkers, particularly when exposed to the harsh reaction conditions and elevated temperature (65 °C) of flow SPPS [15,45].

The library was designed to vary the vinyl sulphide bridge length, direction, and Cys stereochemistry, whereby the analogues were synthesised and tested iteratively (Figure 5). The initial vinyl sulphide analogue (**7**) maintained the ‘bullet’ substitutions of Ala for Cys at positions 9 and 14, and incorporated an additional substitution of Lys for the native Cys at position 5. Secondly, an additional analogue was prepared with 2,3-diaminoproprionic acid (Dap) in position five (**8**). The selection of these analogues was to investigate the importance of the vinyl sulphide bridge length on the activity of ‘bullet’ capitellacin (**5**). The installation of an amine-functionalised side-chain residue (Dap/Lys) to replace the native Cys residue was required to enable the incorporation of the allenamidyl handle (3-butynoic acid [3-BA]), in accordance with our previous work (Cameron et al., 2020 [27]).

Analogues with varied bridge direction were designed to determine if the direction, due to the non-symmetrical nature of the vinyl sulphide bridge, would impact peptide activity or structure. For these analogues, the Cys residues at 9 and 14 were again replaced with Ala, and the Cys residue at position 18 was replaced with either Lys (**9**) or Dap (**10**) to enable allenamide installation (Figure 5).

Finally, we designed a further analogue wherein the importance of the stereochemistry of the Cys residue involved in the vinyl sulphide bridge was investigated. For this analogue (**11**), the Cys residues at positions 9 and 14 were replaced with Ala (to yield a ‘bullet’), and the Cys residue at position 5 was replaced with its d-enantiomer (Figure 5).

For the herein-synthesised vinyl sulphide-substituted peptides, we employed a systematic naming nomenclature that is defined by the residue and the position at which it is replaced; for example, analogue **7**, where Cys^5^ is replaced by Lys, is denoted as analogue ‘Lys5’.

### 2.6. Synthesis of Vinyl Sulphide Analogues **7**–**11**

**Scheme 1:**
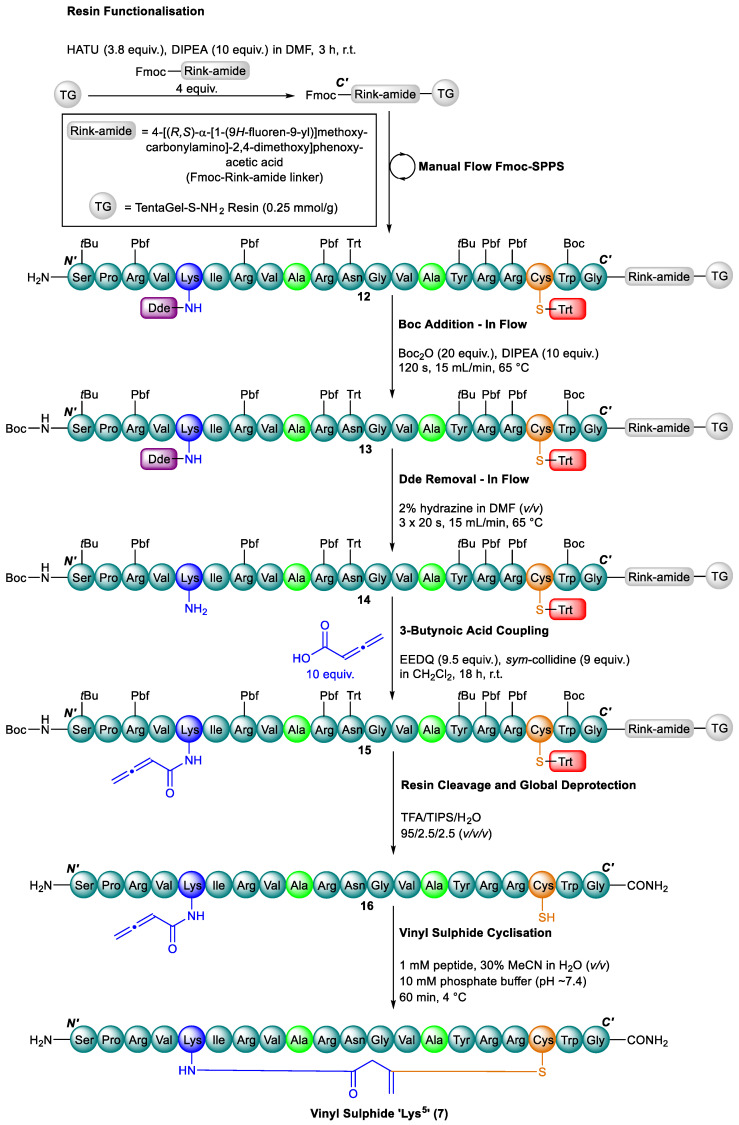
Optimised synthetic route for analogue **7**. Cys residues and thiols are in orange; Ala residues are in bright green; Cys (Trt) protection is in red; Dde protection is in purple; and substituted Cys residues, allenamidyl handle, and vinyl sulphide bridge are in blue.

As a protocol was previously standardised for the synthesis of capitellacin (**1**) [15], and was successfully employed herein for the ‘bullet’ analogue (**5**), all the linear precursors of the vinyl sulphide peptides (**7**–**11**) were synthesised via a similar methodology. The initial vinyl sulphide analogue ‘Lys5’ (**7**) was prepared on a large particle size and high-swelling TentaGel^®^-S-NH_2_ resin (600 mg, 0.15 mmol, 0.25 mmol/g), functionalised with 4-[(R,S)-α-[1-9*H*-flouren-9-yl)]methoylcarbonylamino]-2,4-dimethoxyphenoxyacetic acid (Fmoc–Rink-amide linker) (4 equiv.), using HATU (3.8 equiv.) and DIPEA (10 equiv.) in dimethylformamide (DMF) (3 h, r.t.). Following the functionalisation of the resin, peptide elongation was undertaken identically to that of the previously synthesised analogues (**1**, **3**–**6**) [15]. In order to enable the site-selective introduction of the allenamidyl handle by the coupling of 3-BA, orthogonal protection with a 1-(4,4′-dimethyl-2,6-dioxocyclohexylidene)-3-ethyl (Dde) group was introduced to Lys^5^ (Scheme 1). Following the complete elongation of the peptide under these conditions, an examination of a small portion of the resin beads by TFA-mediated resin cleavage revealed the successful synthesis of the resin-bound linear peptide (**12**) with high purity (Supplementary Figure S36).

As Fmoc-Lys(*N^ε^*-Dde)-OH was employed for the synthesis of analogue **7**, the conditions chosen for orthogonal deprotection were hydrazine in DMF (2%, *v*/*v*). During the deprotection of an *N^ε^*-Dde-protected amine effected by hydrazine, an undesired loss of the *N^α^*-Fmoc group typically occurs. It is possible to circumvent this non-specific reaction through the incorporation of the *N*-terminus as a Boc-protected *N^α^*-amine. For the synthesis of analogue **7**, it was envisaged that the terminal Fmoc protection could be exchanged for Boc for the *N*-terminal Ser^1^, followed by hydrazine removal of *N^ε^*-Dde from the side chain of Lys^5^ (Scheme 1). Following the removal of Fmoc from Ser^1^, a Boc exchange was performed with di-*tert*-butyl dicarbonate (Boc_2_O) (3.0 mmol, 20 equiv.) in DMF (30 mL, 65 °C) in the presence of DIPEA (1.5 mmol, 10 equiv.), at a flow rate of 15 mL/min (Scheme 1). Subsequent *N^ε^*-Dde removal was undertaken with 2% hydrazine (*v*/*v*) in DMF (3 × 20 s, 65 °C), at a flow rate of 15 mL/min (Scheme 1). Pleasingly, the *N*-terminal Boc exchange and *N^ε^*-Dde-removal methodologies could be performed ‘in-flow’ to afford the desired reaction products (**13** and **14**) (Supplementary Figure S37), providing the first reported example of applying these chemical reactions using Pentelute’s and co-workers’ fast-flow Fmoc-SPPS approach [46].

The coupling of 3-BA as the allenamidyl handle was performed in accordance with the optimised protocol of Cameron et al. (2020) [27], employing 3-BA (10 equiv.), EEDQ (9.5 equiv.), and sym-collidine (9 equiv.) in dry dichloromethane (CH_2_Cl_2_). The reaction took place overnight (18 h), affording a near-quantitative conversion (Scheme 1, Supplementary Figure S38). With the final on-resin linear peptide intermediate (**15**) in hand, TFA/TIPS/H_2_O (95:2.5:2.5, *v*/*v*/*v*, 3 h, r.t.) liberated the desired peptide (**16**) from the resin (Scheme 1).

**Scheme 2:**
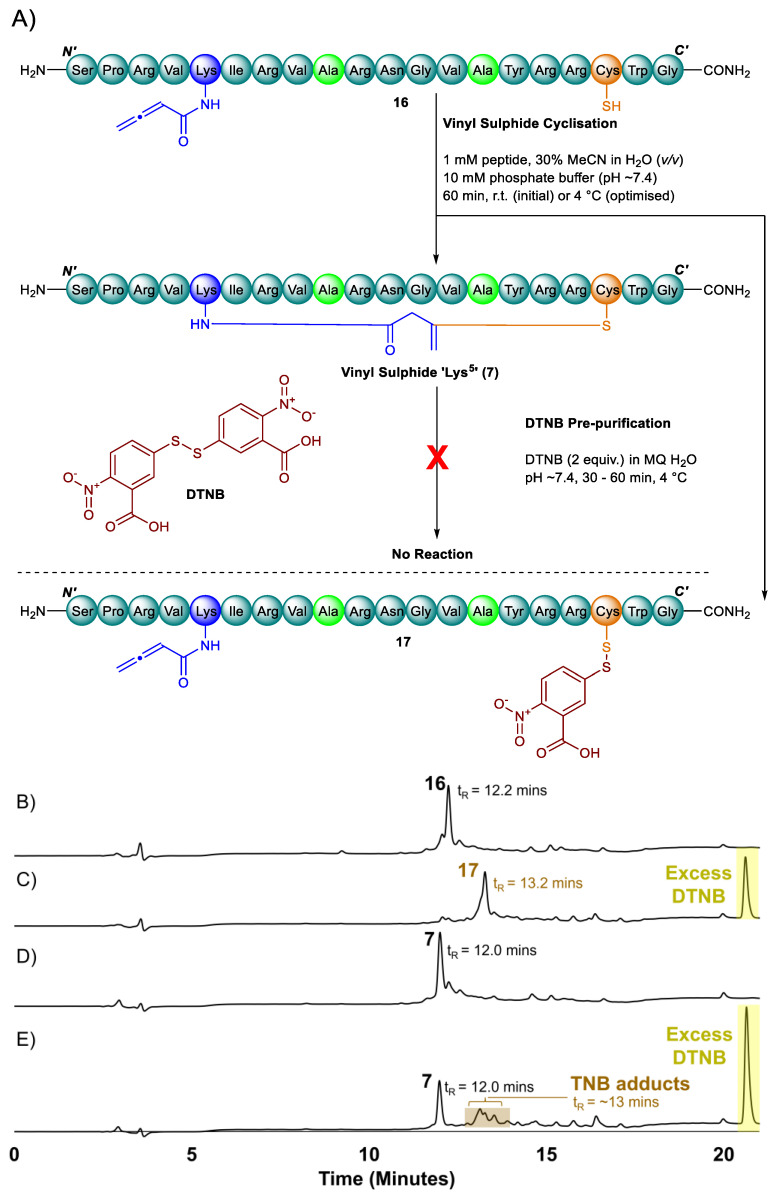
(**A**) Cyclisation of intermediate **16** into peptide **7**, with the addition of DTNB pre-purification. Cys residues and thiols are in orange; Ala residues are in bright green; DTNB/TNB adducts are in brown; and substituted Cys residues, allenamidyl handles, and vinyl sulphide bridges are in blue. (**B**) Intermediate 16 prior to treatment with phosphate buffer. (**C**) Intermediate 16 treated with DTNB prior to treatment with phosphate buffer. (**D**) Crude peptide **7** following cyclisation with phosphate buffer at r.t. (**E**) Crude peptide 7 following cyclisation with phosphate buffer and treatment with DTNB at 4 °C. TNB adducts are in gold) and excess DTNB is in yellow. Phenomenex Luna C18 column (100 Å, 5 µm, 4.6 mm × 250 mm), with a linear gradient of 5% B to 65% B over 20 min (ca. 3% B/min) at a flow rate of 1 mL/min.

The initial attempts to effect the intramolecular cyclisation of ‘Lys5’ (**7**) employed the prior conditions we used to prepare a vinyl sulphide-cyclised oxytocin mimic (Cameron et al., 2020 [27]). A portion of the crude lyophilised peptide (**16**) was subjected to a phosphate buffer (10 mM, pH ~7.4) at r.t. for 60 min (Scheme 1). The reaction progress was monitored by RP-HPLC with the addition of 5,5-dithio-bis-(2-nitrobenzoic acid), (DTNB) employed for reaction quenching (Scheme 2A). Pleasingly, the reaction progressed with a high conversion to the desired product (80–90%, as indicated by RP-HPLC) (Scheme 2, Supplementary Figure S39). Upon the addition of DTNB to the reaction solution, undesired side products bearing a free thiol that eluted with a similar retention time as the desired product, were significantly shifted. Accordingly, the DTNB addition enabled a more straightforward purification by semi-preparative RP-HPLC. The validity of the free thiol test employing DTNB is indicated in Scheme 2B-E, where the starting material (**16**), which was exposed to the same conditions and exhibited a significant retention shift, indicated the presence of a free thiol. The cyclised analogue ‘Lys5’ (**7**) was purified by semi-preparative RP-HPLC to yield the final isolated product with high purity (>95%). The purified product appeared as a single product as judged by RP-HPLC and ESI-MS (Supplementary Figure S40).

^1^H NMR spectroscopy was employed as a final tool to establish definitive confirmation of the desired vinyl sulphide product (**7**), easily characterised by the unique vinylic protons appearing as a pair of singlets between δ 6.0–δ 5.0 ppm when examined in d6-dimethylsulfoxide (DMSO-_d6_) [26,27,47]. It should be noted that for the NMR of peptides, H_2_O:D_2_O (9:1, *v*/*v*) is most commonly employed as the solvent system, with the use of water suppression by excitation sculpting [48]. However, for peptides containing vinylic protons in the range δ 6.0–δ 5.0 ppm, we observed that water suppression significantly distorts the vinylic proton signals and their subsequent integration, thus requiring the use of an alternative solvent, namely, DMSO-_d6_. Upon the examination of the initial portion of purified ‘Lys^5^’ (**7**), the ^1^H NMR spectrum revealed four signals within the vinyl proton region, suggesting the formation of isomers during vinyl sulphide cyclisation (Supplementary Figure S41). A similar phenomenon was observed in our prior work (Cameron et al., 2020 [27]), when undertaking the first reaction of an allene with thiophenol. The first pair of resonances (δ 5.14 ppm and δ 4.89 ppm) were identified as the vinylic protons of the desired product. However, the additional two signals (δ 6.24 ppm and δ 5.84 ppm) were identified as the C^α^*H* of the undesired α,β-unsaturated thermodynamic products, occurring as discrete *E*/*Z* isomers (Supplementary Figure S41). The additional peaks (δ 5.60 ppm and δ 5.49–δ 5.47 ppm) in the vinylic proton region were found to be unrelated to the vinyl sulphide bond, and were also found to occur in the ^1^H NMR spectrum of the ‘bullet’ analogue (**5**) (Supplementary Figure S27), suggesting they result from the amino acid sequence of capitellacin (**1**). This unexpected isomerisation may have occurred as a result of the reduced reaction kinetics during a more challenging cyclisation, requiring up to 1 h for completion, where Cys residues have typically reacted to completion within 10 min in prior works [26,27,47].

Given that the other product isomers were determined to be the thermodynamically favoured products, we opted to decrease the reaction temperature to 4 °C, in order to favour the formation of the desired kinetic product, which occurs as a single isomer. A second portion of the crude lyophilised peptide (**16**) was again subjected to a phosphate buffer (10 mM, pH ~7.4); however, prior to the addition of the buffer, the individual buffer and peptide solutions were pre-cooled to 4 °C. Analogous to the initial cyclisation performed at r.t., a high conversion to the desired product (*ca.* 80–90%) was again observed after 60 min, and the reaction was quenched by the addition of DTNB (Supplementary Figure S42). The cyclised analogue ‘Lys^5^’ (**7**) was purified by semi-preparative RP-HPLC to yield the final isolated product, with purity > 95% (Supplementary Figures S6 and S17). Pleasingly, the ^1^H NMR analysis revealed a single pair of vinyl proton resonances (δ 5.15 ppm and δ 4.89 ppm), confirming the formation of the desired kinetic isomer (**7**) as the sole reaction product (Supplementary Figure S28).

Following the successful synthesis of ‘Lys5’ (**7**), analogues ‘Dap5’ (**8**), ‘Lys18’ (**9**), and ‘Lys18-d-Cys5’ (**11**) were prepared in an analogous fashion (Scheme 1), employing our newly optimised cyclisation procedure at 4 °C. Following purification by semi-preparative RP-HPLC, peptides **8**, **9,** and **11** were confirmed by analytical RP-HPLC and ESI-MS (Supplementary Figures S7–S11, S18–S21). To our delight, ^1^H NMR spectroscopy evidenced the robustness of the 4 °C cyclisation method, as all the final products were observed as single products, evidenced by the presence of a sole pair of vinylic proton signals (Supplementary Figures S29, S30, and S32).

**Figure 6:**
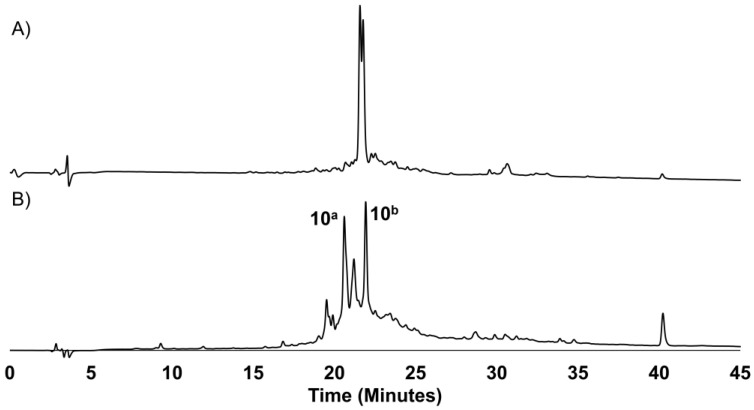
Analytical RP-HPLC chromatograms (214 nm) of the initial attempt to prepare analogue **10** incorporating Dap^18^ with *N^β^*-Dde protection. (**A**) Post-elongation by flow synthesis (including Dde removal), following cleavage of a small portion of the resin beads. (**B**) Following cyclisation in phosphate buffer (10 mM, pH 7.4). Phenomenex Luna C18 column (100 Å, 5 µm, 4.6 mm × 250 mm) with a linear gradient of 5% B to 45% B over 40 min (ca. 1%/min) at a flow rate of 1 mL/min. ^a/b^ denotes the *m*/*z* of the corresponding peptidyl products of identical mass; the desired product was unable to be determined.

Unfortunately, following the ‘in-flow’ protocol synthesis of the ‘Dap18’ (**10**) analogue, analysis by RP-HPLC and ESI-MS upon cleaving a small portion of the resin revealed two peaks of identical masses prior to the installation of the allenamidyl handle (Figure 6A and Supplementary Figures S43–S45). Although the two peaks were indistinguishable by the observed *m*/*z*, synthesis of the peptide continued under the previously employed protocol to produce the final vinyl sulphide-cyclised peptide (Figure 6B). Unfortunately, despite the peaks being resolvable by an analytical RP-HPLC and exhibiting an *m/z* corresponding to the desired product for ‘Dap18’ (**10**), these results suggest the presence of isomers which could not be easily identified unambiguously.

**Scheme 3:**
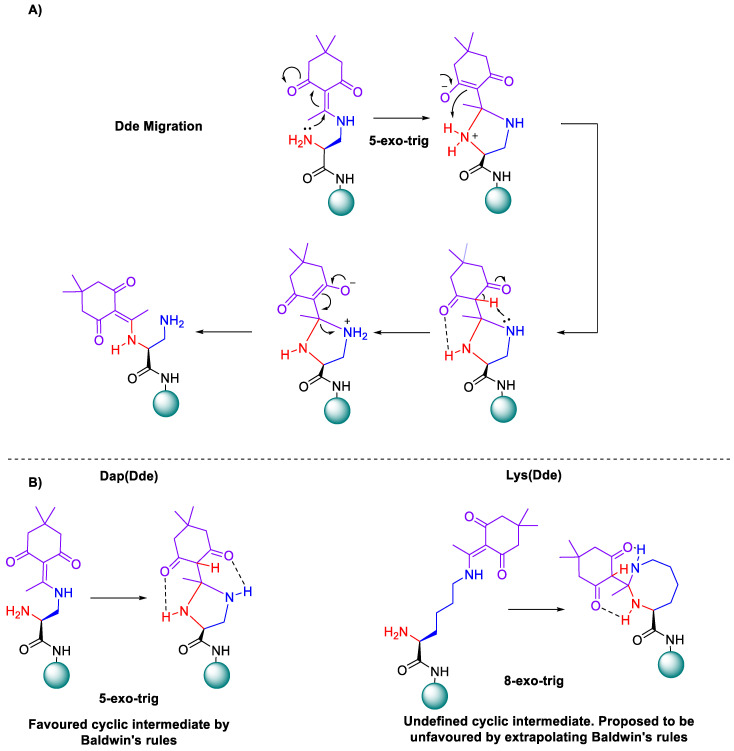
(**A**) Proposed migration of Dde (purple) from side chain (blue) to *N*-terminus (red) of Dap (Dde) during fast-flow Fmoc-SPPS elongation of analogue **10**. Resin is shown in teal. (**B**) Cyclic intermediates of Dde-protected Dap and Lys, highlighting expectedly favourable and unfavourable intermediates [50].

The Dde protecting group has previously been shown to migrate [49]. Thus, we speculated that the isomers of the growing peptide chain may have formed due to the migration of the Dde group from the *N^β^*-side-chain amino group to the *N*-terminus of the Dap^18^ residue during/following the removal of *N^α^*-Fmoc protection from the Dap^18^ residue under fast-flow Fmoc-SPPS conditions (Scheme 3A). Conceivably, the undesired formation of this product may have been accelerated by the increased temperature implemented during flow synthesis (65 °C). It should be noted that the formation of this isomer was only observed for ‘Dap18’ (**10**), with no evidence of its formation being present during the preparation of the Lys analogues (**7** and **9**). Accordingly, we believe this may be due to the favourable formation of a 6-membered ring during migration [50] (Scheme 3). In contrast, the cyclic intermediate of the Lys intermediate has not been defined as favourable; however, extrapolating Baldwin’s rules [50] suggests that it is likely unfavourable (Scheme 3B), explaining the lack of migration observed during the preparation of analogues **7** and **9**. The resulting partial migration and subsequent elongation of the peptide via the side chain would result in a peptide of identical mass, but largely structurally different, thus explaining our observations. Interestingly, however, during the synthesis of ‘Dap5’ (**8**), migration was not observed after incorporating Dap(*N^β^*-Dde), suggesting the migration may be sequence-specific, and perhaps the bulky nature of the β-branched Ile^6^ residue preceding the Dap^5^ residue could have hindered Dde migration in analogue **8**.

To avoid problematic Dde migration during fast-flow Fmos-SPPS, we proposed that the best strategy would be to change the orthogonal side-chain protecting group (Dde). We determined that *N^β^*-Alloc protection would offer the desired orthogonality, as it has not been reported to undergo migration as it is not prone to nucleophilic attack. Furthermore, the *N^β^*-Alloc-protected derivative of Fmoc-Dap is commercially available, and the orthogonal removal of the *N^β^*-Alloc group has been well documented as occurring quantitatively in a batch-wise reaction [51] upon treatment with tetrakis(triphenylphosphine)palladium(0) (Pd[PPh_3_]_4_) and phenylsilane (PhSiH_3_) in CH_2_Cl_2_ after 2 h at r.t.

Pleasingly, following the flow synthesis of the linear sequence of ‘Dap18’ (**10**) incorporating *N^β^*-Alloc protection, a single product with the desired *m/z* was observed (Supplementary Figures S43 and S46). *N^β^*-Alloc removal was quantitatively performed following a modified procedure from Abdel Monaim et al. (2017) [51]. The modified procedure employed an increased excess of Pd(PPh_3_)_4_ from 0.1 equiv. to 4 equiv. to drive rapid and quantitative conversion, as the excess reagents could simply be removed by resin filtration and washing. Following the successful orthogonal deprotection of Dap^18^, the coupling of 3-BA, resin cleavage and global deprotection, and cyclisation proceeded smoothly. Following purification by semi-preparative RP-HPLC, peptide **10** was confirmed by analytical RP-HPLC and ESI-MS (Supplementary Figure S46), completing the analogue library (Supplementary Table S3). Much to our satisfaction, ^1^H NMR spectroscopy established that **10** occurred as a single product, as evidenced by the presence of the two vinylic protons (Supplementary Figure S31). Accordingly, we propose the Alloc group as a superior route for the incorporation of orthogonally protected Dap residues, especially during microwave synthesis or fast-flow Fmoc-SPPS.

### 2.7. Biological Investigation of Analogues **7**–**11**

To investigate the effect of the replacement of the disulphide bond with a vinyl sulphide mimetic, analogues **7**–**11** were tested, alongside capitellacin (**1**) and the ‘bullet’ analogue (**5**), for their activity towards *P. aeruginosa*, *E. coli*, *S. aureus*, and *C. albicans*. Capitellacin (**1**) and the ‘bullet’ analogue (**5**) were employed as reference compounds with which to derive SAR data. PMB, AMX, and AMB were once again employed as the antimicrobial controls (Table 2).

Similar to the native (**1**) and ‘bullet’ (**5**) analogues, no activity was observed in any of the vinyl sulphide analogues (**7**–**11**) towards Gram-positive *S. aureus* or the fungus *C. albicans* (Table 2). This finding is in accordance with the previous literature, where there is little evidence for disulphide bridge replacements substantially broadening the activity spectrum of β-hairpin AMPs [24,52]. When examining antimicrobial activity towards the Gram-negative pathogens, all the vinyl sulphide analogues (**7**–**11**) showed diminished activity when compared to the native (**1**) peptide and ‘bullet’ (**5**) analogue. Compound **9** retained the greatest potency among the vinyl sulphide analogues, with an MIC of 4 µM and 32 µM towards *E. coli* and *P. aeruginosa*, respectively, and a 2-fold reduction in potency compared to the ‘bullet’ analogue (**5**) (Table 2). All the vinyl sulphide analogues demonstrated an MIC of 32 µM towards *P. aeruginosa*, which is 2-fold less active than the equivalent disulphide analogue (**5**). As capitellacin (**1**) and analogues **5** and **7**–**11** showed the greatest potency and variations in activity towards *E. coli*, we determined it was best to examine the *E. coli* MIC results for trends with respect to the importance of the vinyl sulphide length, direction, and Cys stereochemistry for activity.

From the MIC results for the vinyl sulphide analogues (**7**–**11**), we were able to reach several conclusions. For the vinyl sulphide analogues (**7** and **8**) where Cys^5^ was replaced with amine-functionalised side-chain residues (Dap/Lys), the bridge length did not affect antimicrobial activity. Comparatively, for the vinyl sulphide analogues (**9** and **10**) where Cys^18^ was replaced with an amine-functionalised side-chain residue (Dap/Lys), a longer bridge length exhibited a better maintenance of activity. Finally, we observed the importance of stereochemistry, where modifying the stereochemistry from l- to d- for the Cys residue (**11**) completely abolished activity, and suggests it may have more dramatically influenced the peptide backbone conformation. Similar results and decreases in activity have been observed in other previous works, where disulphide surrogates, including lactam bridges, mixed thioether/disulphide analogues, and 1,4-triazoles, were implemented in related β-hairpin scaffolds. Similar reductions in potency have been observed for the lactam-cyclised analogues of gomesin. The greater structural flexibility imparted by the lactam bridge over the disulphide bridge reduced the activity of gomesin analogues over that of the native one [24]. Liu and co-workers [52] investigated disulphide replacement in tachyplesin I with dicarba and thioether/disulphide surrogates in a synthetic strategy inspired by the synthesis of lantibiotics. They found most of the analogues possessed moderately reduced antimicrobial activity and shifts in their secondary structures, despite maintaining the same atom count in their cyclic bridges. The replacement of the disulphides in tachyplesin I with 1,4-triazoles also maintained similar potency to that of the native peptide [25]. It is noteworthy that these prior studies investigated bicyclic analogues that are inherently more similar to the native peptide than the simplified, monocyclic vinyl sulphide analogues of capitellacin presented herein. While introducing greater structural flexibility with bridges chemically distinct from the native disulphide often leads to a reduction in activity, akin to our own findings with capitellacin, the degree of this reduction appears heavily dependent on the specific β-hairpin scaffold and the position of the replaced bridge [24,25,52].

Analogous to the disulphide analogues of capitellacin (**3**–**6**), examining the retention time of the vinyl sulphide analogues (**7**–**11**) revealed a relationship between the hydrophobicity of a peptide and its activity (Supplementary Table S4). Once again, increased hydrophobicity (as judged by RP-HPLC retention time) correlated with increased potency towards *E. coli*. Although it is more subtle than for the disulphide analogues (**3**–**6**), it supports the possibility that the RP-HPLC retention time may be used as a tool to rapidly predict potential analogue activity when developing capitellacin (**1**) or similar β-hairpin AMPs as drug candidates [53,54].

### 2.8. Circular Dichroism Investigation of Analogues **7**–**11**

**Figure 7:**
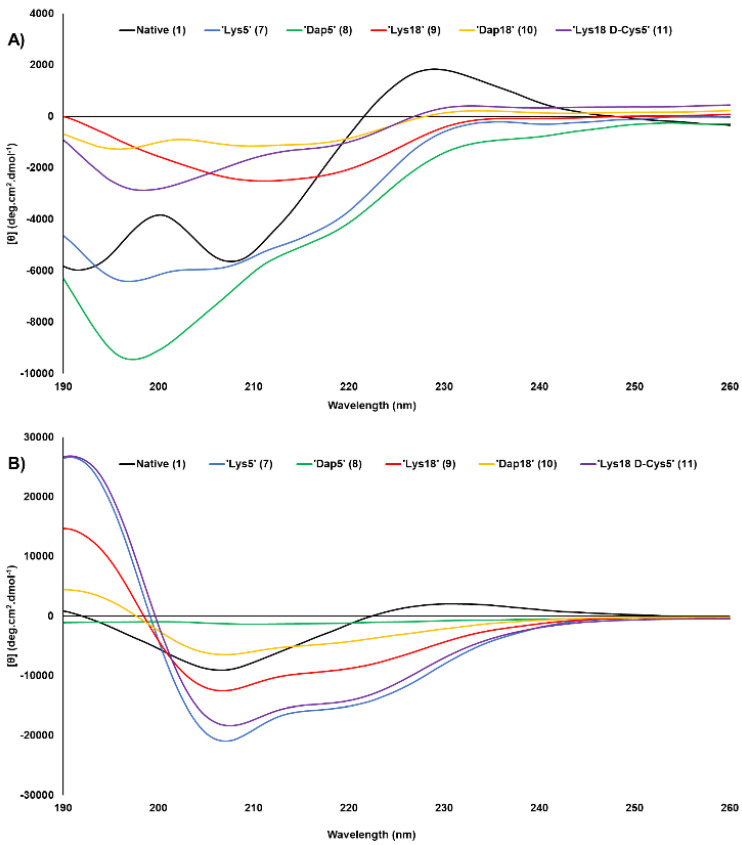
(**A**) CD spectra overlay of capitellacin (**1**) and analogues **7**–**11** at 50 µM in 200 µM sodium phosphate buffer (pH ~7.4, 20 °C). (**B**) CD spectra overlay of capitellacin (**1**) and analogues **7**–**11** at 50 µM in TFE:200 µM sodium phosphate buffer 1:1 (pH ~7.4, 20 °C).

To determine the effect of the vinyl sulphide bridge on the secondary structure of capitellacin (**1**), we examined analogues **7**–**11** by CD. The spectra were again collected in two solvent systems, sodium phosphate buffer (pH ~7.4, 200 µM) and sodium phosphate buffer/TFE (1:1, *v*/*v*, pH ~7.4, 200 µM) (Figure 7). Capitellacin (**1**) was used as a ’fingerprint’ to provide a qualitative measure of the change in the secondary structure upon modification of the disulphide bridge with vinyl sulphide moieties (Figure 7).

In the buffer alone (Figure 7A), all the analogues (**7**–**11**) exhibited substantial deviation from the native secondary structure, likely existing predominantly as random coil structures, with no minima or maxima observed to provide a definitive means of identifying the common secondary structure elements. For the buffer, this result was not unexpected, as it was hypothesised that the vinyl sulphide bridge would provide more flexibility over the disulphide, resulting in a less defined secondary structure. However, when examined in the secondary structure-favouring environment (Figure 7B), the vinyl sulphide bridge analogues (**7**–**11**) exhibited more defined spectral characteristics. The spectra exhibited similar negative maxima to capitellacin (**1**) at ~208 nm, suggesting that the analogues (**7**–**11**) can adopt a secondary structure similar to that of capitellacin (**1**). This result suggests the vinyl sulphide analogues (**7**–**11**) may adopt a similar active conformation to capitellacin (**1**) upon membrane binding, as TFE is known to mimic this interaction [39]. Noteworthy, however, a second negative maxima near 222 nm were observed for analogues **7**–**10**, suggesting they may also possess helical secondary structure elements. A further point of interest is the high degree of similarity between analogues **7** and **11**, for which the spectra are almost identical. This was surprising given the variation in bridge length and direction, as well as the inclusion of the d-Cys residue in **11**, which impacted antimicrobial activity to a more varying degree. Liu and co-workers also observed variation in the CD spectra upon the disulphide substitution of tachyplesin I, even when the total atom count of the bridge was maintained. Although many of the hydrogen bonds in the β-hairpin structures were lost upon bridge substitution, a similar overall hairpin-like shape was evidenced by NMR, and the bioactivity was only moderately reduced [52].

## 3. Material and Methods

### 3.1. General Information

For general procedures and materials, see the Supplementary Materials. Common chemical abbreviations used throughout include the following: (4-hydroxymethyl)phenylacetic acid (HMPA); 4-dimeyhylaminopyridine (DMAP); dimethylformamide (DMF); dichloromethane (CH_2_Cl_2_); 1,4-dioxane (dioxane); diethyl ether (Et_2_O); 3-butynoic acid (3-BA); solid-phase peptide synthesis (SPPS); iodine (I_2_); 2,4,6-trimethylpyridine (*sym*-collidine); trifluoroacetic acid (TFA); acetamidomethyl (Acm); Milli-Q water (MQ H_2_O); *N*,*N*′-diisopropylethylamine (DIPEA); (7-azabenzotriazol-1-yloxy)trispyrrolidinophosphonium (PyAOP); diemthylsulfoxide (DMSO); triisopropylsilane (TIPS); 1-[bis(dimethylamino)methylene]-1*H*-1,2,3-triazole [4,5-b]pyridinium 3-oxide hexafluorophosphate (HATU); *tert*-butyloxycabonyl (Boc); *tert*-butyl (*t*Bu); fluorenyl-methoxycarbonyl protecting group (Fmoc); pentamethyl-2,3-dihydrobenzofuran-5-sulfonyl (Pbf); triphenylmethyl (Trt); phenylsilane (PhSiH_3_); *N*-ethoxycarbonyl-2-ethoxy-1,2-dihydroquinoline (EEDQ); di-*tert*-butyl decarbonate (Boc_2_O); trifluoroethanol (TFE); 5,5-dithio-bis-(2-nitrobenzoic acid) (DTNB, Ellman’s Reagents); 4-[(R,S)-α-[1-9*H*-flouren-9-yl)]methoylcarbonylamino]-2,4-dimethoxyphenoxyacetic acid (Fmoc–Rink-amide linker); 1-(4,4′-dimethyl-2,6-dioxocyclohexylidene)-3-ethyl (Dde); tetrakis(triphenylphosphine)palladium(0) (Pd(PPh_3_)_4_); and 2,3-diaminoproprionic acid (Dap). For chemical structure of common abbreviations employed, see Supplementary Figure S47 and for fast-flow peptide synthesis setup see Supplementary Figure S48.

### 3.2. Peptide Synthesis

All linear peptides were prepared by fluorenyl-methoxycarbonyl protecting group (Fmoc)–SPPS using a manually operated flow-chemistry apparatus, employing TentaGel^®^-S-NH_2_ resin at ca. 0.15 mmol scale. Disulphide cyclisation was performed using a directed method with iodine in varying solvents [15]. Vinyl sulphide cyclisation was performed at a physiological pH (~7.4) in MeCN:MQ H_2_O (3:7, *v*/*v*) at 4 °C. See the Supplementary Materials for further details.

### 3.3. MIC (Minimum Inhibitory Concentration) Assay—Bacteria

*Staphylococcus aureus* ATCC 29213, *Pseudomonas aeruginosa* (SVB-B9) (type strain), and *Escherichia coli* ATCC 25922 were grown in cation-adjusted Mueller Hinton (CA-MHB) broth, at 37 °C with shaking (200 rpm). MIC assays were performed in accordance with the CLSI recommended protocols [55]. See the Supplementary Materials for further details.

### 3.4. MIC (Minimum Inhibitory Concentration) Assay—Fungus

*Candida albicans* SC5314 (type strain) was grown using RPMI 1640 media (with glutamine and phenol red, without bicarbonate). MIC assays were performed in accordance with the CLSI recommended protocol [55]. See the Supplementary Materials for further details.

### 3.5. Circular Dichroism (CD)

All CD spectra were recorded in either phosphate buffer (pH ~7.4, 200 µM) or TFE/phosphate buffer (pH ~7.4, 200 µM, 1:1, *v*/*v*) at a peptide concentration of 50 µM. All spectra were recorded at 20 °C with a cuvette of 1 mm path length in a range from 180 nm to 260 nm at 0.5 nm intervals with a time-to-point of 0.5 s. Each spectrum was prepared from an average of five scans obtained with a 1 nm optical bandwidth. The baseline scans were collected with solvent alone, averaged, and then subtracted from the sample scans.

### 3.6. NMR Spectroscopy

NMR spectroscopy was performed using peptide samples of 0.5 mM in DMSO-_d6_ at 298 K. Chemical shifts were reported in parts per million (ppm) and referenced to DMSO-_d6_, at 2.50 ppm.

## 4. Conclusions

Building on our previous total synthesis of the β-hairpin AMP capitellacin (**1**), we herein explored the chemical space surrounding this disulphide-cyclised peptide by preparing two focused analogue libraries, where we varied the number of intramolecular cyclisations, as well as their replacement with a vinyl sulphide. The most terminal disulphide, evident in the ‘bullet’ analogue (**5**), was determined to be the most critical to the activity of capitellacin (**1**). Notably, this analogue exhibited a more specific spectrum of activity towards *E. coli*, favourable for many clinical applications. Furthermore, our work highlights the enantiomeric specificity of capitellacin’s activity, whereby stereo-inversion to yield the d-enantiomer substantially reduced activity. Combining this result with the determination of capitellacin’s mechanism supports the idea of the inherent chirality of the bacterial membrane playing an important role in activity. We note that the diminished activity may also be due to a more enantio-specific secondary mechanism, which remains unknown.

Furthermore, we report the first example of bioactive peptides, in particular, antimicrobial peptides, cyclised by a vinyl sulphide bridge. In preparing the vinyl sulphide analogues of capitellacin (**7**–**11**), the methodology for cyclisation was refined by the intramolecular Michael addition of a Cys thiol to an allenamide, enabling efficient peptide cyclisation with a minimally explored disulphide surrogate. Additionally, methods were developed for orthogonal side-chain modifications during fast-flow Fmoc-SPPS, enabling the removal of Dde by hydrazine in DMF, as well as Fmoc to Boc *N*′-terminal exchange. Furthermore, we observed substantial protecting group migration of *N^β^*-Dde- protected Dap during fast-flow Fmoc-SPPS, which we proposed occurs rapidly due to a favourable 5-exo-trig pathway. We reported that the use of the Alloc group successfully avoids this undesired migration, while still offering the required protecting group orthogonality for short-chain diamino acids, such as Dap. The vinyl sulphide mimetics maintained varying levels of antimicrobial potency, specifically toward *E. coli*, again suggesting a strategy for developing valuable narrow-spectrum antibiotics. We additionally used these analogues to explore the unique asymmetric nature of the vinyl sulphide bond. Varying the bridge length, direction, and stereochemistry impacted activity, highlighting the potential of vinyl sulphide as a method for the fine-tuning of bioactive peptides.

For both our analogue libraries, the bioactivity results were supported by trends in the secondary structures (observed by circular dichroism) and hydrophobicity, suggesting the potential of predictive analysis for future analogues of capitellacin (**1**).

## Figures and Tables

**Table 1:** Minimum inhibitory concentrations of capitellacin (**1**) and analogues **3**–**6** towards selected bacterial and fungal pathogens.

Minimum Inhibitory Concentrations (µM)									
Compounds									
	1 (Recombinant) ^a^	1 (Synthetic)	3	4	5	6	PMB *	AMX *	AMB *
Gram positive									
*S. aureus* ATCC 25913	-	>64	>64	>64	>64	>64	-	2	-
*S. aureus* ATCC 25923	8	-	-	-	-	-	-	-	-
Gram negative									
*E. coli* ATCC 25922	0.5	1	4	>64	1	4	0.125	-	-
*P. aeruginosa* SVB-B9 ^T^	-	4	8	>64	16	64	0.125	-	-
*P. aeruginosa* PAO1	2	-	-	-	-	-	-	-	-
Fungi									
*C. albicans* SC5314 ^R^	-	64	>64	>64	>64	>64	-	-	1

^a^ Values cited from Panteleev et al. (2020) [12]. ^T^ Type strain. ^R^ Reference Strain. * polymyxin B (PMB), amoxicillin (AMX), and amphotericin B (AMB).

**Table 2:** Minimum inhibitory concentrations (MIC) of capitellacin (**1**) and analogues **5**, **7**–**11** towards selected bacterial and fungal pathogens.

	Minimum Inhibitory Concentrations (µM)									
	Compounds									
	1 (Synthetic)	5 (‘bullet’)	7 (‘Lys^5^’)	8 (‘Dap^5^’)	9 (‘Lys^18^’)	10 (‘Dap^18^’)	11 (‘Lys^18^-d-Cys^5^’)	PMB *	AMX *	AMB *
	Gram positive									
*S. aureus* ATCC 25913	>64	>64	>64	>64	>64	>64		-	2	-
*S. aureus* ATCC 25923	8	-	-	-	-	-		-	-	-
	Gram negative									
*E. coli* ATCC 25922	1	1	8	8	4	8	>64	0.125	-	-
*P. aeruginosa* SVB-B9 ^T^	4	16	32	32	32	32	>64	0.125	-	-
*P. aeruginosa* PAO1	2	-	-	-	-	-		-	-	-
	Fungi									
*C. albicans* SC5314 ^R^	64	>64	>64	>64	>64	>64	>64	-	-	1

^T^ Type strain. ^R^ Reference Strain. * polymyxin B (PMB), amoxicillin (AMX), and amphotericin B (AMB).

## Data Availability

The original contributions presented in the study are included in the article/Supplementary Material, further inquiries can be directed to the corresponding authors.

## Supplementary Materials

The following supporting information can be downloaded at: https://www.mdpi.com/article/10.3390/antibiotics13070615/s1, File S1: Supplementary Figures S1–S48 and Supplementary Tables S1–S4. References [12,27,34,46,51,55,56] are cited in the Supplementary Materials.
