# Supplementary material for: The Antimicrobial Peptide Capitellacin: Chemical Synthesis of Analogues to Probe the Role of Disulphide Bridges and Their Replacement with Vinyl Sulphides
Source: Antibiotics (Basel). 2024 Jul 2;13(7):615. doi: 10.3390/antibiotics13070615 (PMC11273936; doi:10.3390/antibiotics13070615)
Supplement: Supplementary file 1 [file antibiotics-13-00615-s001.zip › antibiotics-3039473-supplementary.pdf]

Antimicrobial Peptide Capitellacin: Chemical Synthesis of Analogues to Probe the Role of Disulphide Bridges and Their Replacement with Vinyl Sulphides.

Oscar Shepperson[1,2,3], Paul Harris[1,2,3], Margaret Brimble[1,2,3*], and Alan Cameron[1,2,3,*]

[1]The University of Auckland, School of Chemical Sciences, 23 Symonds St., Auckland 1010, New Zealand.

[2]The University of Auckland, School of Biological Sciences, 3A Symonds St., Auckland 1010, New Zealand.

[3]Maurice Wilkins Centre for Molecular Bio-Discovery. The University of Auckland, 3A Symonds St. Auckland 1010, New Zealand.

*** Correspondence:**
Alan Cameron
alan.cameron@auckland.ac.nz

Alternative correspondence

Margaret Brimble

m.brimble@auckland.ac.nz

Contents

1 General Information 4
2 General Methods 4
2.1 Method 1: Resin Functionalisation for *C*-terminal acid peptides 5
2.2 Method 2: Resin Functionalisation for *C*-terminal carboxamide peptides 5
2.3 Method 3: Manual Flow Synthesis of Linear Peptides 5
Sub-Method 3.1 – Cys coupling 6
2.4 Method 4: On-resin Disulphide Bond Formation between Cys(Trt) Residues 6
2.5 Method 5: On-resin Disulphide Bond Formation between Cys(Acm) Residues 6
2.6 Method 6: In-flow N-terminal Boc Protection 6
2.7 Method 7: In-flow Orthogonal Removal of Dde from Dap/Lys by Hydrazine 6
2.8 Method 8: Orthogonal Removal of N^{β}-Alloc from Dap by Palladium (0) 6
2.9 Method 9: 3-Butynoic Acid Coupling 6
2.10 Method 10: Resin Cleavage and Global Deprotection 6
2.11 Method 11: Solution Phase Vinyl Sulphide Cyclisation 6
2.12 Method 12: Quenching of Unreacted Thiols by DTNB 7
3 Protocol for Synthesis of Peptides 1, 3 – 11 8
3.1 Native Capitellacin (1) 8
3.2 D-enantiomeric Capitellacin (3) 9
3.3 'Linear' Analogue (4) 10
3.4 'Bullet' Analogue (5) 11
3.5 'Kite' Analogue (6) 12
3.6 Vinyl Sulphide Analogue, 'Lys^{5}' (7) 13
3.7 Vinyl Sulphide Analogue, 'Dap^{5}' (8) 14
3.8 Vinyl Sulphide Analogue, 'Lys^{18}' (9) 15
3.9 Vinyl Sulphide Analogue, 'Dap^{18}' (10) 16
Initial Protocol Using Fmoc-Dap(N^{β}-Dde)-OH 16
Revised Protocol Using Fmoc-Dap(N^{β}-Dde)-OH 17
3.10 Vinyl Sulphide Analogue, 'Lys^{18}-D-Cys^{5}' (11) 18
4 Spectral Data 19
4.1 RP-HPLC Data for Peptides 1, 3 – 11 19
4.2 ESI-MS Data for Peptides 1, 3 – 11 25
4.3 HRMS of Analogues 1, 3 – 6 35

4.4 ^{1}H NMR Data for Analogues 5, 7 – 11 40
5 Antimicrobial Testing of Capitellacin (1) and Analogues 3 – 11 46
5.1 Antibacterial Testing 46
5.2 Antifungal Testing 46
6 Circular Dichroism of Capitellacin (1) and Analogues 3 - 11 47
6.1 CD Spectra of peptides 1, 3 – 6 47
7 Further Supplementary Material 49
7.1 Additional ^{1}H NMR, RP-HPLC Traces and ESI-MS 50
7.2 Data Summary of Synthetic Peptides 57
7.3 Abbreviation Structures 62
8 Flow Chemistry Setup 63
9 References 64

Supplementary Material

1 General Information

All the reagents purchased from commercial sources were reagent grade and were used without further purification. Solvents for peptide synthesis and RP-HPLC were purchased as synthesis grade and HPLC grade, respectively.

4-[(*R,S*)-α-[1-(9*H*-Fluoren-9-yl)]methoxycarbonylamino]-2,4-dimethoxy]phenoxyacetic acid (Fmoc-Rink amide linker), polymyxin B sulphate (PMB), tetrakis(triphenylphosphine)palladium(0) ($Pd(PPh_3)_4$), 4-dimethylaminopyridine (DMAP), (4-Hydroxymethyl)phenylacetic acid (HMPA linker) and *N*-ethoxycarbonyl-2-ethoxy-1,2-dihydroquinoline (EEDQ) were purchased from AK Scientific (Union City, CA, USA). (7-Azabenzotriazol-1-yloxy)trispyrrolidinophosphonium hexafluorophosphate (PyAOP), 1-hydroxy-6-chloro-benzotriazole (6-Cl-HOBt) and di-*tert*-butyl dicarbonate (Boc_2O) were purchased from Aapptec (Louisville, KY, USA). *N,N*-diisopropylethylamine (DIPEA), 2,4,6-trimethylpyridine (*sym*-collidine), triisopropylsilane (TIPS), piperidine, 3-butynoic acid (3-BA), hydrazine, 5,5-dithio-bis-(2-nitrobenzoic acid) (DTNB, Ellman's Reagent), amphotericin B (AMB), phosphate buffer saline (PBS), phenylsilane ($PhSiH_3$), sodium diethyldithiocarbamate, trifluoroethanol (TFE), amoxicillin (AMX), *N,N'*-diisopropylcarbodiimide (DIC) and formic acid (FA) were purchased from Sigma–Aldrich (St Louis, MO, USA). Diethyl ether (Et_2O) was purchased from Avantor Performance Materials (Center Valley, USA). Trifluoroacetic acid (TFA) was purchased from Oakwood Chemicals (Estill, SC, USA). Oxoid Mueller Hinton Broth (MHB), *N,N*-dimethylformamide (DMF; AR grade) and acetonitrile (MeCN, HPLC grade) were purchased from Thermo Scientific (Hampshire, NH, USA). Deuterochloroform ($CDCl_3$), dimethylsulfoxide-$_{d6}$ (DMSO-$_{d6}$) and deuterium oxide (D_2O) were purchased from Cambridge Isotopes (Tewksbury, MA, USA). Dimethylsulfoxide (DMSO), iodine (I_2) and dichloromethane (CH_2Cl_2) were purchased from ECP Limited (Auckland, New Zealand). TentaGel®-S-NH_2 resin was purchased from RAPP Polymere (Tubingen, Germany). Polypropylene 96-well flat bottom plates were purchased from Greiner Bio-One (Kremsmünster, Austria). Milli-Q high purity deionised water (MQ H_2O) was available from a Sartorius Arium® Pro Ultrapure Water System from Sartorius Stedim Biotech (Gottingen, Germany). 1-[Bis(dimethylamino)methylene]-1*H*-1,2,3-triazolo[4,5-*b*]pyridinium 3-oxide hexafluorophosphate (HATU), Boc-Gly-OH (Boc = *tert*-butyloxycabonyl), and all Fmoc-amino acids were purchased from CS Bio Ltd (Shanghai, China). The supplied amino-acids were side chain protected as follows: Fmoc-Arg(Pdf)-OH (Pbf = 2,2,4,6,7-pentamethyldihydrobenzofuran-5-sulfonyl), Fmoc-Asn(Trt)-OH, (Trt = triphenylmethyl), Fmoc-Cys(Trt)-OH, Fmoc-Cys(Acm)-OH (Acm = acetamidomethyl), Fmoc-Ser(*t*Bu)-OH (*t*Bu = *tert*-butyl), Fmoc-Trp(Boc)-OH, Fmoc-Lys(Boc)-OH, Fmoc-Lys(Dde)-OH (Dde = 1-(4,4'-dimethyl-2,6-dioxocyclohexylidene)-3-ethyl), Fmoc-Gln(Trt)-OH, Fmoc-Dap(N^{β}-Alloc)-OH (N^{β}-Alloc = *N*-Allyloxycarbonyl) and Fmoc-Tyr(*t*Bu)-OH).

Analytical reverse phase high-performance liquid chromatography (RP-HPLC) was performed on a Waters (Waltham, MA, USA) Alliance analytical HPLC equipped with a Phenomenex (Torrance, CA, USA) Luna C18 column (100 A, 5 µm, 4.6 mm x 250 mm) operated at room temperature, with chromatograms recorded at 214 nm and 254 nm. Semi-preparative RP-HPLC was performed on a Waters 1525 Binary HPLC pump equipped with a Waters 2489 UV/visible detector (214 nm) using a Phenomenex Luna C18 semi-prep column (100 Å, 5 µm, 250 mm x 10 mm). For both analytical and semi-preparative RP-HPLC, solvents used were as follows: solvent A = 0.1% TFA in water (MQ H_2O) and solvent B = 0.1% TFA in MeCN. For analytical RP-HPLC the gradient employed was 5-45% of solvent B over 40 minutes at flow rate of 1 mL/min, unless specified otherwise. For semi-preparative RP-HPLC gradients were adjusted as indicated in the experimental procedures, according to elution times and peak profiles obtained during analytical analysis. Flow setup specifications were as follow; VICI M series pump (Model M50, P/N CP3A-8182-F2), VICI 10 port switching valve (Model C25, P/N C25-6180EUHB), 1/8” OD tubing between switching valve and pump and 1/16” OD tubing for all other setup areas. The reaction vessel was manufactured according to the specifications as outlined by Simon *et al.* (2014).

High-resolution mass spectrometry (HRMS) was performed with a Bruker (Billerica, MA, USA) micrOTOFQ mass spectrometer by using electrospray ionisation (ESI) in the positive mode at a nominal accelerating voltage of 70 eV. Low-resolution mass spectrometry was performed on a Waters Quattro micro–API Mass Spectrometer in ESI positive mode.

Nuclear magnetic resonance (NMR) spectroscopy was performed using a *ca.* 0.5 mM sample of peptides (**4.1 – 4.5**) in DMSO-$_{d6}$. Nuclear magnetic resonance (NMR) experiments were recorded at 298 K using a Bruker (Billerica, MA, USA) ASCEND 500 spectrometer operating at 500 MHz for ^{1}H nuclei. Chemical shifts were reported in parts per million (ppm) and referenced to DMSO, at 2.50 ppm.

2 General Methods

2.1 Method 1: Resin Functionalisation for *C*-terminal acid peptides

Manual flow synthesis was performed on a *ca.* 0.15 mmol scale. Loading of HMPA linker (110.0 mg, 0.60 mmol, 4 equiv.) to TentaGel®-S-NH_2 resin (600 mg, 0.15 mmol, 0.25 mmol/g) was undertaken prior to flow-SPPS using 6-Cl-HOBt (101.7 mg, 0.60 mmol, 4 equiv.) and DIC (92.9 µL, 0.60 mmol, 4 equiv.) in DMF for 2 h at r.t. Following linker coupling, the resin was washed with DMF (3 x 5 mL) and CH_2Cl_2 (3 x 5 mL). The symmetrical anhydride of the N^{α}-Fmoc protected *C*-terminal amino acid was prepared from Fmoc-AA-OH (1.5 mmol, 10 equiv.) by reacting with DIC (116.1 µL, 0.75 mmol, 5 equiv.) in CH_2Cl_2 for 20 min at 0 °C. The resulting symmetric anhydride (0.75 mmol, 5 equiv.) was then manually esterified with the resin bound HMPA linker via acyl transfer, using a catalytic amount of DMAP (9.2 mg, 0.075 mmol, 0.5 equiv.) in DMF for 2 h at r.t. The peptidyl resin was washed with DMF (3 x 5 mL) and CH_2Cl_2 (3 x 5 mL). Peptide elongation was performed by manual flow synthesis (**Method 3**).

2.2 Method 2: Resin Functionalisation for *C*-terminal carboxamide peptides

Manual flow synthesis was performed on a *ca.* 0.15 mmol scale. Loading of the Fmoc-Rink amide linker (324 mg, 0.60 mmol, 4 equiv.) to TentaGel®-S-NH_2 resin (600 mg, 0.15 mmol, 0.25 mmol/g) was undertaken prior to flow-SPPS using HATU (216 mg, 0.57 mmol, 3.8 equiv.) and DIPEA (261 µL, mmol, 10 equiv.) in DMF for 3 h at r.t. Following linker coupling the resin was washed with DMF (3 x 5 mL) and CH_2Cl_2 (3 x 5 mL). Peptide elongation was performed by manual flow synthesis (**Method 3**).

2.3 Method 3: Manual Flow Synthesis of Linear Peptides

During manual flow synthesis, the deprotection solution for removal of the N^{α}-Fmoc protecting group consisted of 30% piperidine (*v/v)* in DMF. Prior to beginning the synthesis, amino acid coupling solutions were freshly prepared in DMF at a concentration of 0.30 M of amino acid (1.5 mmol, 10 equiv.) and 0.28 M of coupling reagent (HATU, 1.43 mmol, 9.5 equiv.). Prior to each amino acid coupling, DIPEA (522 µL, 3.00 mmol, 20 equiv.) was added to pre-activate the coupling solution 10 seconds prior to delivery to the on-resin peptide bearing a free N^{α}-amino group.

Reagents were delivered via a 5 ml stainless steel heating loop submerged in a water bath (65 °C), to the stainless-steel reaction vessel, also submerged in a water bath (65 °C), containing the functionalised peptidyl resin. All reagents were delivered at a constant flow rate of 15 mL/min unless specified otherwise. N.B; *Sub-Method 3.1***.**

The manual flow synthesis began with pre-washing the resin with DMF (300 s, 75 mL) and performing the initial deprotection of the Fmoc protected amino acid or Rink amide linker, as appropriate (40 s, 10 mL). Following Fmoc-Rink amide deprotection, the resin was washed again with DMF (40 s, 10 mL).

Amino acids were coupled in order from the *C*- to *N*-terminus via four repeating steps of; (**1**) coupling, (**2a/b**) washing, (**3**) deprotection and (**4**) washing; outlined as follows:

(1) The activated amino acid solution is coupled to the free *N*-terminus of the previous residue/linker of the peptidyl resin (25-30 s, 6.6 mL).

(2)

- **a.** The delivery line for the activated amino acid solution is washed by drawing DMF (15 s, 3.8 mL).
- **b.** The peptidyl resin is further washed with DMF, removing any residual activated amino acid coupling solution (40 s, 10 mL).

(3) The N^{α}-Fmoc protecting group is removed from the newly coupled amino acid with the deprotection solution of 30% piperidine in DMF (*v/v*) (40 s, 10 mL).

(4) Peptidyl resin is washed, removing any residual deprotection solution (40 s, 10 mL).

Following the completion of peptide sequence the N^{α}-Fmoc protected resin bound peptide was washed with DMF (300 s, 75 mL, 65 °C), to remove any possible remaining residual reagents.

Sub-Method 3.1 – Cys coupling

The coupling of the Fmoc-Cys(Trt)-OH/Fmoc-Cys(Acm)-OH residues (0.30 M, 1.50 mmol, 10 equiv.) was performed with PyAOP (0.28 M, 1.43 mmol 9.5 equiv.) and *sym*-collidine (396.4 µL, 3.00 mmol, 20 equiv.) at a flow rate of 15 mL/min at 65 °C.

2.4 Method 4: On-resin Disulphide Bond Formation between Cys(Trt) Residues

The on-resin peptide was thoroughly washed with DMF (2 x 5 ml) and swelled in dioxane before being twice treated (2 x 5 min) with a fine powder of iodine (114.2 mg, 0.45 mmol, 3 equiv.) dissolved in dioxane (5 mL). Following iodine treatments, the resin was thoroughly washed with dioxane (3 x 5 mL), 30% piperidine in DMF (*v/v*) (2 x 5 mL) and finally the resin was washed with DMF (3 x 5 mL), CH_2Cl_2 (3 x 5 mL) and dried under vacuum.

2.5 Method 5: On-resin Disulphide Bond Formation between Cys(Acm) Residues

The on-resin peptide was thoroughly washed with DMF (2 x 5 ml) and swelled in DMF before being twice treated (2 x 5 min) with a fine powder of iodine (114.2 mg, 0.45 mmol, 3 equiv.) dissolved in DMF (5 mL). Following iodine treatments, the resin was thoroughly washed with DMF (3 x 5 mL), 30% piperidine in DMF (*v/v*) (2 x 5 mL) and finally the resin was washed with DMF (3 x 5 mL), CH_2Cl_2 (3 x 5 mL) and dried under vacuum.

2.6 Method 6: In-flow N-terminal Boc Protection

The unprotected N^{α}-resin was treated with a solution of Boc_2O (327 mg, 3 mmol, 20 equiv.) in DMF (120 s, 30 mL, 65 °C), activated with DIPEA (522 µL, 1.5 mmol, 10 equiv.) at a flow rate of 15 mL/min. Following treatment, the resin bound peptide was washed with DMF (300 s, 75 mL, 65 °C) to remove any residual reagents.

2.7 Method 7: In-flow Orthogonal Removal of Dde from Dap/Lys by Hydrazine

The resin was treated with a solution of 2% hydrazine (*v/v*) in DMF (3 x 20 s, 65 °C) at a flow rate of 15 mL/min. Following the final treatment, the resin bound peptide was washed with DMF (300 s, 75 mL, 65 °C) to remove any residual reagents.

2.8 Method 8: Orthogonal Removal of N^{β}-Alloc from Dap by Palladium (0)

The protected peptidyl resin bearing the N^{β}-Alloc protected Dap residue was swollen in dry CH_2Cl_2 (dried over molecular sieves). To the swollen resin was added a mixture of $Pd(PPh_3)_4$ (231.1 mg, 0.60 mmol, 4 equiv.) and $PhSiH_3$ (185 µL, 1.50 mmol, 10 equiv.) in dry CH_2Cl_2 and the reaction mixture agitated under an inert atmosphere, in the dark for 2 h at r.t. Following reaction completion, $Pd(PPh_3)_4$ was quenched with treatments of 0.5% sodium diethyldithiocarbamate (*w/v*) in DMF (3 x 3 min) washed with DMF (3 x 5 mL), CH_2Cl_2 (3 x 5 mL) and dried under vacuum (Abdel Monaim et al., 2017).

2.9 Method 9: 3-Butynoic Acid Coupling

The protected peptidyl resin containing a free side chain amino group was swollen in dry CH_2Cl_2 (dried over molecular sieves). To the swollen peptidyl resin was added a mixture of 3-butynoic acid (126 mg, 1.5 mmol, 10 equiv.), EEDQ (352 mg, 1.43 mmol, 9.5 equiv.) and *sym*-collidine (178 µL, 1.35 mmol, 9 equiv.) in dry CH_2Cl_2 and the reaction mixture agitated at r.t. for 3 – 18 h as outlined by Cameron *et al.* (2020). Following coupling, the peptidyl resin was thoroughly washed with CH_2Cl_2 (3 x 5 mL), DMF (3 x 5 mL) and again with CH_2Cl_2 (3 x 5 mL) and dried under vacuum.

2.10 Method 10: Resin Cleavage and Global Deprotection

The resin-bound peptide was treated with a cleavage cocktail of TFA/H_2O/TIPS (10 mL, 9.5/2.5/2.5, *v/v/v*) and agitated (3 h, r.t.). The cleavage solution was separated from the resin and its volume reduced under a flow of N_2. Cold Et_2O (45 mL) was used to precipitate the peptide. The pellet was isolated by centrifugation, washed once more with cold Et_2O (45 mL), dissolved in MeCN/MQ H_2O (3:7, *v/v*) and lyophilised.

2.11 Method 11: Solution Phase Vinyl Sulphide Cyclisation

The crude allenamide modified peptide was dissolved in a solution of MeCN/MQ H_2O (3:7, *v/v*) at 4 °C. To the solution was added 200 mM phosphate buffer (1/20 total vol., pH ~7.4, 4 °C) to maintain the final solution at a concentration of 1 mM peptide and 10 mM buffer. The solution was maintained at 4 °C for 60 min, reaction progression monitored by RP-HPLC, and quenched upon completion with DTNB according to **Method 12.**

2.12 Method 12: Quenching of Unreacted Thiols by DTNB

To the vinyl sulphide cyclisation solution was added a concentrated solution of DTNB (5 mg/mL, 4 °C, *ca.* 2 equiv.) in MQ H_2O. The solution was mixed for 30 – 60 mins at 4 °C, monitored by RP-HPLC, and upon observed reaction completion lyophilised.

3 Protocol for Synthesis of Peptides 1, 3 – 11

3.1 Native Capitellacin (1)

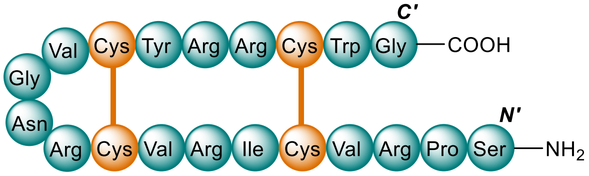

Capitellacin (1)

Following the functionalisation of TentaGel®-S-NH_2 resin with HMPA linker and the *C*-terminal Gly residue by **Method 1**, capitellacin (**1**), was synthesised by manual flow synthesis according to **Method 3** employing **Sub-Method 3.1** for the introduction of Cys(Trt) at positions 9 and 14 and Cys(Acm) at positions 5 and 18. Following flow synthesis, the peptidyl resin was split into three portions. Taking 0.05 mmol of the resin, the disulphide bonds were introduced asynchronously by **Method 4** followed by **Method 5** in the order of $Cys^{9/14}$ followed by $Cys^{5/18}$. The final peptide was liberated from the resin and protecting groups simultaneously removed following **Method 6**, to afford the crude capitellacin (**3.1**) as an off-white powder (45 mg, 38% yield [based on initial resin loading], *ca.* 60% purity).

Crude capitellacin, **1**, (45 mg) was dissolved in 1:9 MeCN/H_2O at a concentration of 4 mg/ml and purified (6 x ~2 mL injections) with a Waters Xterra Prep MS C18 OBD column (125 Å, 10 µm, 10 mm x 250 mm), employing a gradient of 5%B to 47%B over 14 minutes (*ca*. 3%B per min) at a flow rate of 5 mL/min. Fractions were analysed by ESI-MS for compound identification. Fractions from multiple runs were combined and lyophilised to afford the parent compound, capitellacin (**1**), as a white amorphous powder (11.7 mg, 26% recovery [based on crude yield], > 97% purity, 10% overall yield accounting for resin splitting).

ESI-MS: *m/z* calculated for [$C_{99}H_{159}N_{37}O_{24}S_4$] 2379.1; deconvoluted observed: 2379.1 ± 0.2. Charge states; 595.8 $[M+4H]^{4+}$, 794.1 $[M+3H]^{3+}$.

HRMS (ESI/Q-TOF): *m/z* calculated for $[C_{99}H_{159}N_{37}O_{24}S_4 + 4H]^{4+}$ 595.5383; observed: 595.5395.

RP-HPLC: t_R = 12.7 min. Phenomenex Luna C18 (100Å, 5 µm, 4.6 mm x 250 mm), linear gradient 5%B to 65%B over 20 min (*ca.* 3%B/min) at 1 mL/min.

3.2 D-enantiomeric Capitellacin (3)

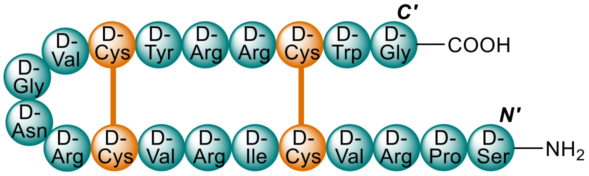

D-Capitellacin (3)

Following the functionalisation of TentaGel®-S-NH_2 resin with HMPA linker and the *C*-terminal Gly residue by **Method 1**, peptide **3** was synthesised by manual flow synthesis according to **Method 3** employing **Sub-Method 3.1** for the introduction of Cys(Trt) at positions 9 and 14 and Cys(Acm) at positions 5 and 18. Disulphide bonds were formed asynchronously by **Method 4** followed by **Method 5** in the order of $Cys^{9/14}$ followed by $Cys^{5/18}$. The final peptide was liberated from the resin and protecting groups simultaneously removed following **Method 5**, to afford crude peptide (**3**) as an off-white powder (90 mg, 25% yield [based on initial resin loading], *ca.* 55% purity).

Crude **3** (90 mg) was dissolved in 1:9 MeCN/H_2O at a concentration of ~5 mg/ml and purified (9 x ~2 mL injections) with a with a Phenomenex Luna C18 column (100 Å, 5 µm, 250 mm x 10 mm), employing a gradient with an isocratic phase of 5%B for 5 min, followed by a linear gradient to 65%B/min (*ca.* 1%B/min) at a flow rate of 5 mL/min. Fractions were analysed by ESI-MS for compound identification. Fractions from multiple runs were combined and lyophilised to afford the desired compound, **3**, as a white amorphous powder (3.4 mg, 3.8% recovery [based on crude yield], > 98% purity, 0.9% overall yield).

ESI-MS: *m/z* calculated for [$C_{99}H_{159}N_{37}O_{24}S_4$] 2379.1; deconvoluted observed: 2379.1 ± 0.2. Charge states; 476.9 $[M+5H]^{5+}$, 595.9 $[M+4H]^{4+}$, 794.1 $[M+3H]^{3+}$.

HRMS (ESI/Q-TOF): *m/z* calculated for $[C_{99}H_{159}N_{37}O_{24}S_4 + 4H]^{4+}$ 595.5400; observed: 595.5383.

RP-HPLC: t_R = 12.6 min. Phenomenex Luna C18 (100Å, 5 µm, 4.6 mm x 250 mm), linear gradient 5%B to 65%B over 20 min (*ca.* 3%B/min) at 1 mL/min.

3.3 'Linear' Analogue (4)

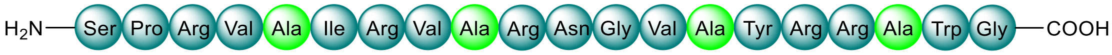

'Linear' Capitellacin (4)

Following the functionalisation of TentaGel®-S-NH_2 resin with HMPA linker and the *C*-terminal Gly residue by **Method 1**, peptide **4** was synthesised by manual flow synthesis according to **Method 3**. The final linear peptide was liberated from the resin and protecting groups simultaneously removed following **Method 5**, to afford the crude peptide (**4**) as an off-white powder (140 mg, 41% yield [based on initial resin loading], *ca.* 85% purity).

Crude **4** (35 mg) was dissolved in a solution of 1:9 MeCN/MQ H_2O at a concentration of ~5 mg/mL and purified (4 x ~2 mL injections) with a Phenomenex Luna C18 column (100 Å, 5 µm, 250 mm x 10 mm), employing a gradient of 5%B/min to 47%B/min (*ca.* 3%B/min) at a flow rate of 5 mL/min. Fractions were analysed by RP-HPLC and ESI-MS for compound identification. Fractions from multiple runs were combined and lyophilised to afford the desired compound, **4**, as a white amorphous powder (18.0 mg, 13% recovery [based on crude yield], > 97% purity, 5.3% overall yield).

ESI-MS: *m/z* calculated for [$C_{99}H_{163}N_{37}O_{24}$] 2255.2; deconvoluted observed: 2255.7 ± 0.3. Charge states; 452.2 $[M+5H]^{5+}$, 564.9 $[M+4H]^{4+}$, 752.8 $[M+3H]^{3+}$.

HRMS (ESI/Q-TOF): *m/z* calculated for $[C_{99}H_{163}N_{37}O_{24} + 4H]^{4+}$ 564.5755; observed: 564.5741.

RP-HPLC: t_R = 11.7 min. Phenomenex Luna C18 (100Å, 5 µm, 4.6 mm x 250 mm), linear gradient 5%B to 65%B over 20 min (*ca.* 3%B/min) at 1 mL/min.

3.4 'Bullet' Analogue (5)

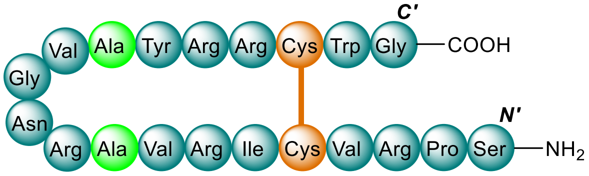

'Bullet' Capitellacin (5)

Following the functionalisation of TentaGel®-S-NH_2 resin with HMPA linker and the *C*-terminal Gly residue by **Method 1**, peptide **5** was synthesised by manual flow synthesis according to **Method 3** employing **Sub-Method 3.1** for the introduction of Cys(Trt) at positions 5 and 18. The disulphide bond was formed by **Method 4**. The final peptide was liberated from the resin and protecting groups simultaneously removed following **Method 5**, to afford the crude peptide (**5**) as an off-white powder (204 mg, 59% yield [based on initial resin loading], *ca.* 85% purity).

Crude **5** (73 mg) was dissolved in 1:9 MeCN/H_2O at a concentration of ~5 mg/ml and purified (8 x ~2 mL injections) with Phenomenex Luna C18 column (100 Å, 5 µm, 250 mm x 10 mm), employing a gradient with an isocratic phase of 5%B for 5 min, followed by a linear gradient to 50%B/min (*ca.* 1%B/min) at a flow rate of 5 mL/min. Fractions were analysed by ESI-MS for compound identification. Fractions from multiple runs were combined and lyophilised to afford the desired compound, **5**, as a white amorphous powder (25.1 mg, 12% recovery [based on crude yield], > 98% purity, 7.2% overall yield).

ESI-MS: *m/z* calculated for [$C_{99}H_{161}N_{37}O_{24}S_2$] 2317.2; deconvoluted observed: 2317.1 ± 0.1. Charge states; 773.6 $[M+3H]^{3+}$,1159.9 $[M+2H]^{2+}$.

HRMS (ESI/Q-TOF): *m/z* calculated for $[C_{99}H_{161}N_{37}O_{24}S_2 + 4H]^{4+}$ 580.0551; observed: 580.0562.

RP-HPLC: t_R = 25.6 min. Phenomenex Luna C18 (100Å, 5 µm, 4.6 mm x 250 mm), linear gradient 5%B to 45%B over 40 min (*ca.* 1%B/min) at 1 mL/min.

3.5 'Kite' Analogue (6)

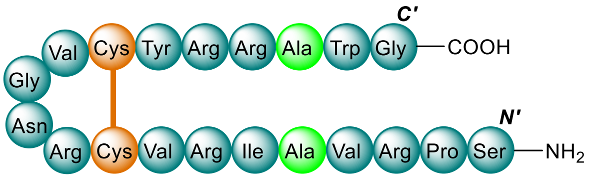

'Kite' Capitellacin (6)

Following the functionalisation of TentaGel®-S-NH_2 resin with HMPA linker and the *C*-terminal Gly residue by **Method 1**, peptide **6** was synthesised by manual flow synthesis according to **Method 3** employing **Sub-Method 3.1** for the introduction of Cys(Trt) at positions 9 and 14. The disulphide bond was formed by **Method 3**. The final peptide was liberated from the resin and protecting groups simultaneously removed following **Method 5**, to afford the crude peptide (**6**) as an off-white powder (102 mg, 29% yield [based on initial resin loading], *ca.* 85% purity).

Crude **6** (102 mg) was dissolved in 1:9 MeCN/H_2O at a concentration of ~5 mg/ml and purified (10 x ~2 mL injections) with a Phenomenex Luna C18 column (100 Å, 5 µm, 250 mm x 10 mm), employing a gradient of 5%B/min to 47%B/min (*ca.* 3%B/min) at a flow rate of 5 mL/min. Fractions were analysed by ESI-MS for compound identification. Fractions from multiple runs were combined and lyophilised to afford the desired compound, **6**, as a white amorphous powder (13.0 mg, 13% recovery [based on crude yield], > 98% purity, 3.7% overall yield).

ESI-MS: *m/z* calculated for [$C_{99}H_{161}N_{37}O_{24}S_2$] 2317.2; deconvoluted observed: 2317.2 ± 0.1. Charge states; 464.7 $[M+5H]^{5+}$, 580.6 $[M+4H]^{4+}$, 773.7 $[M+3H]^{3+}$.

HRMS (ESI/Q-TOF): *m/z* calculated for $[C_{99}H_{161}N_{37}O_{24}S_2 + 4H]^{4+}$ 580.0558; observed: 580.0562.

RP-HPLC: t_R = 14.8 min. Phenomenex Luna C18 (100Å, 5 µm, 4.6 mm x 250 mm), linear gradient 5%B to 65%B over 20 min (*ca.* 3%B/min) at 1 mL/min.

3.6 Vinyl Sulphide Analogue, 'Lys^{5}' (7)

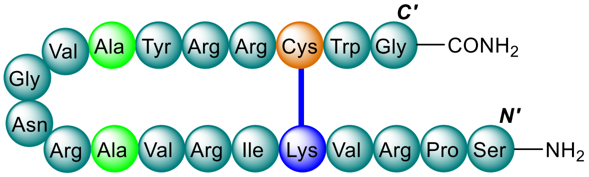

Vinyl Sulphide 'Lys^{5}' (7)

Following the functionalisation of the TentaGel®-S-NH_2 resin with Fmoc-Rink amide linker via **Method 2**, peptide **7** was synthesised by manual flow synthesis according to **Method 3** employing **Sub-Method 3.1** for the introduction of Cys(Trt) at position 18. The *N*-terminus was Boc protected via **Method 6**. N^{ε}-Dde was orthogonally removed from Lys^{5} via **Method 7** and the allenamide handle was coupled via **Method 9** to the side chain of residue 5. The final linear peptide was liberated from the resin and protecting groups simultaneously removed following **Method 10**, to afford the crude linear peptide as a slightly pink powder (232 mg, 74% yield [based on initial resin loading]). The crude linear peptide was cyclised via **Method 11** and quenched via **Method 12** to yield the crude desired peptide (**7**) as a slightly yellow powder at approximately 70% purity).

The crude peptide (270 mg), **7**, was dissolved in a solution of 1:9 MeCN/MQ H_2O at a concentration of ~4 mg/mL and purified (16 x ~4 mL injections) with a Phenomenex Luna C18 column (100 Å, 5 µm, 250 mm x 10 mm), employing a gradient with an isocratic phase of 5%B for 5 min, followed by a linear gradient to 50%B/min (*ca.* 1%B/min) at a flow rate of 5 mL/min. Fractions were analysed by RP-HPLC and ESI-MS for compound identification. Fractions from multiple runs were combined and lyophilised to afford the desired compound, **7**, as a white amorphous powder (14.8 mg, 5.5% recovery [based on crude yield], > 98% purity, 4.1% overall yield).

ESI-MS: Mass calculated for [$C_{106}H_{173}N_{39}O_{24}S$] 2409.3; deconvoluted observed: 2410.1 ± 0.1. Charge states; 603.5 $[M+4H]^{4+}$, 804.4 $[M+3H]^{3+}$.

RP-HPLC: t_R = 23.8 min. Phenomenex Luna C18 (100Å, 5 µm, 4.6 mm x 250 mm), linear gradient 5%B to 45%B over 40 min (*ca.* 1%B/min) at 1 mL/min.

3.7 Vinyl Sulphide Analogue, 'Dap^{5}' (8)

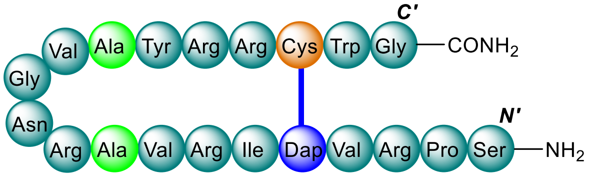

Vinyl Sulphide 'Dap^{5}' (8)

Following the functionalisation of the TentaGel®-S-NH_2 resin with Fmoc-Rink amide linker via **Method 2**, peptide **8** was synthesised by manual flow synthesis according to **Method 3** employing **Sub-Method 3.1** for the introduction of Cys(Trt) at position 18. The *N*-terminus was Boc protected via **Method 6**. N^{β}-Dde was orthogonally removed from Dap^{5} via **Method 7** and the allenamide handle was coupled via **Method 9** to the side chain of residue 5. The final linear peptide was liberated from the resin and protecting groups simultaneously removed following **Method 10**, to afford the crude linear peptide as a slightly pink powder (203 mg, 86% yield [based on initial resin loading]). The crude linear peptide was cyclised via **Method 11** and quenched via **Method 12** to yield the crude desired peptide (**8**) as a slightly yellow powder at approximately 50% purity.

The crude peptide (203 mg), **8**, was dissolved in a solution of 1:9 MeCN/MQ H_2O at a concentration of ~6 mg/mL and purified (17 x ~2 mL injections) with a Phenomenex Luna C18 column (100 Å, 5 µm, 250 mm x 10 mm), employing a gradient with an isocratic phase of 5%B for 5 min, followed by a linear gradient to 50%B/min (*ca.* 1%B/min) at a flow rate of 5 mL/min. Fractions were analysed by RP-HPLC and ESI-MS for compound identification. Fractions from multiple runs were combined and lyophilised to afford the desired compound, **8**, as a white amorphous powder (7.2 mg, 3.5% recovery [based on crude yield], > 98% purity, 2.0% overall yield).

ESI-MS: Mass calculated for [$C_{103}H_{167}N_{39}O_{24}S$] 2367.3; deconvoluted observed: 2368.1 ± 0.1. Charge states; 474.6 $[M+5H]^{5+}$, 593.0 $[M+4H]^{4+}$, 790.3 $[M+3H]^{3+}$.

RP-HPLC: t_R = 24.4 min. Phenomenex Luna C18 (100Å, 5 µm, 4.6 mm x 250 mm), linear gradient 5%B to 45%B over 40 min (*ca.* 1%B/min) at 1 mL/min.

3.8 Vinyl Sulphide Analogue, ‘Lys[18]’ (9)

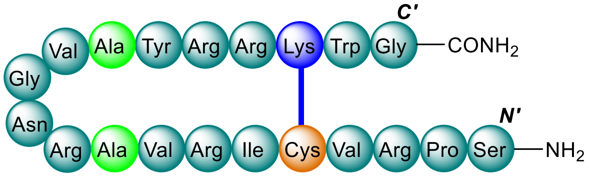

Vinyl Sulphide 'Lys[18]' (9)

Following the functionalisation of the TentaGel®-S-NH_2 resin with Fmoc-Rink amide linker via **Method 2**, peptide **9** was synthesised by manual flow synthesis according to **Method 3** employing **Sub-Method 3.1** for the introduction of Cys(Trt) at position 5. The *N*-terminus was Boc protected via **Method 6**. N^{ε}-Dde was orthogonally removed from Lys^{18} via **Method 7** and the allenamide handle was coupled via **Method 9** to the side chain of residue 18. The final linear peptide was liberated from the resin and protecting groups simultaneously removed following **Method 10**, to afford the crude linear peptide as a slightly pink powder (304 mg, 84% yield [based on initial resin loading]). The crude linear peptide was cyclised via **Method 11** and quenched via **Method 12** to yield the crude desired peptide (**9**) as a slightly yellow powder at approximately 65% purity.

The crude peptide (114 mg), **9**, was dissolved in a solution of 1:9 MeCN/MQ H_2O at a concentration of ~6 mg/mL and purified (10 x ~2 mL injections) with a Phenomenex Luna C18 column (100 Å, 5 µm, 250 mm x 10 mm), employing a gradient with an isocratic phase of 5%B for 5 min, followed by a linear gradient to 50%B/min (*ca.* 1%B/min) at a flow rate of 5 mL/min. Fractions were analysed by RP-HPLC and ESI-MS for compound identification. Fractions from multiple runs were combined and lyophilised to afford the desired compound, **9**, as a white amorphous powder (6.7 mg, 2.2% recovery [based on crude yield], > 95% purity, 1.9% overall yield).

ESI-MS: Mass calculated for [$C_{106}H_{173}N_{39}O_{24}S$] 2409.3; deconvoluted observed: 2410.7 ± 0.3. Charge states; 483.2 $[M+5H]^{5+}$, 603.7 $[M+4H]^{4+}$, 804.5 $[M+3H]^{3+}$.

RP-HPLC: t_R = 24.1 min. Phenomenex Luna C18 (100Å, 5 µm, 4.6 mm x 250 mm), linear gradient 5%B to 45%B over 40 min (*ca.* 1%B/min) at 1 mL/min.

3.9 Vinyl Sulphide Analogue, 'Dap[18]' (10)

Initial Protocol Using Fmoc-Dap(N^{β}-Dde)-OH

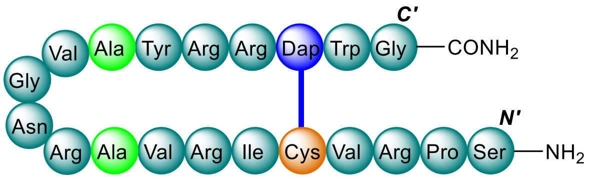

Vinyl Sulphide 'Dap[18]' (10)

Following the functionalisation of the TentaGel®-S-NH_2 resin with Fmoc-Rink amide linker via **Method 2**, peptide **10** was synthesised by manual flow synthesis according to **Method 3** employing **Sub-Method 3.1** for the introduction of Cys(Trt) at position 5. The *N*-terminus was Boc protected via **Method 6**. N^{β}-Dde was orthogonally removed from Dap^{18} via **Method 7** and the allenamide handle was coupled via **Method 9** to the side chain of residue 18. The final linear peptide was liberated from the resin and protecting groups simultaneously removed following **Method 10**, to afford the crude linear peptide was a slightly pink powder (210 mg, 59% yield [based on initial resin loading]). The crude linear peptide was cyclised via **Method 11** and quenched via **Method 12** to yield the crude desired peptide (**10**) as a slightly yellow powder at approximately 40% purity.

The crude peptide (91 mg), **10**, was dissolved in a solution of 1:9 MeCN/MQ H_2O at a concentration of ~6 mg/mL and purified (8 x ~2 mL injections) with a Phenomenex Luna C18 column (100 Å, 5 µm, 250 mm x 10 mm), employing a gradient with an isocratic phase of 5%B for 5 min, followed by a linear gradient to 50%B/min (*ca.* 1%B/min) at a flow rate of 5 mL/min. Fractions were analysed by RP-HPLC and ESI-MS for compound identification. Fractions from multiple runs were combined and lyophilised to afford the desired compound, **10**, as a white amorphous powder (0.6 mg, 0.7% recovery [based on crude yield], > 95% purity, 0.2% overall yield).

ESI-MS: Mass calculated for [$C_{103}H_{167}N_{39}O_{24}S$] 2367.3; deconvoluted observed: 2368.1 ± 0.3. Charge states; 474.7 $[M+5H]^{5+}$, 593.0 $[M+4H]^{4+}$, 790.3 $[M+3H]^{3+}$.

RP-HPLC: t_R = 17.4 min. Phenomenex Luna C18 (100Å, 5 µm, 4.6 mm x 250 mm), linear gradient 5%B to 45%B over 40 min (*ca.* 1%B/min) at 1 mL/min.

Revised Protocol Using Fmoc-Dap(N^{β}-Dde)-OH

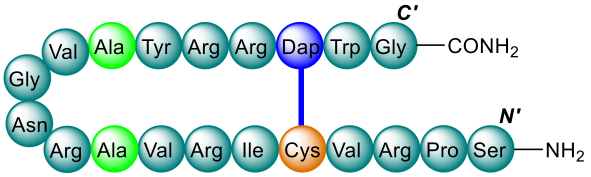

Vinyl Sulphide 'Dap^{18}' (10)

Following the functionalisation of the TentaGel®-S-NH_2 resin with Fmoc-Rink amide linker via **Method 2**, peptide **10** was synthesised by manual flow synthesis according to **Method 3** employing **sub-method 3.1** for the introduction of Cys(Trt) at position 18. The *N*-terminus was Boc protected via **Method 6**. N^{β}-Alloc was orthogonally removed Dap^{18} via **Method 8** and the allenamide handle was coupled via **Method 9** to the side chain of residue 5. The final linear peptide was liberated from the resin and protecting groups simultaneously removed following **Method 10**, to afford the crude linear peptide as a slightly pink powder (198 mg, 56% yield [based on initial resin loading]). The crude linear peptide was cyclised via **Method 11** and quenched via **Method 12** to yield the crude desired peptide (**10**) as a slightly yellow powder at approximately 70% purity.

The crude peptide (73 mg), **10**, was dissolved in a solution of 1:9 MeCN/MQ H_2O at a concentration of ~7 mg/mL and purified (5 x ~2 mL injections) with a Phenomenex Luna C18 column (100 Å, 5 µm, 250 mm x 10 mm), employing a gradient with an isocratic phase of 5%B for 5 min, followed by a linear gradient to 50%B/min (*ca.* 1%B/min) at a flow rate of 5 mL/min. Fractions were analysed by RP-HPLC and ESI-MS for compound identification. Fractions from multiple runs were combined and lyophilised to afford the desired compound, **10**, as a white amorphous powder (6.5 mg, 8.9% recovery [based on crude yield], > 95% purity, 3.5% overall yield).

ESI-MS: Mass calculated for [$C_{103}H_{167}N_{39}O_{24}S$] 2367.3; deconvoluted observed: 2368.1 ± 0.3. Charge states; 474.7 $[M+5H]^{5+}$, 593.0 $[M+4H]^{4+}$, 790.3 $[M+3H]^{3+}$.

RP-HPLC: t_R = 24.4 min. Phenomenex Luna C18 (100Å, 5 µm, 4.6 mm x 250 mm), linear gradient 5%B to 45%B over 40 min (*ca.* 1%B/min) at 1 mL/min.

3.10 Vinyl Sulphide Analogue, ‘Lys[18]-D-Cys[5]’ (11)

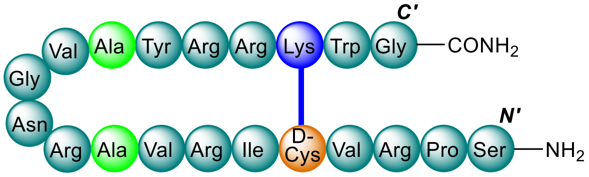

Vinyl Sulphide 'Lys[18]-D-Cys[5]' (11)

Following the functionalisation of the TentaGel®-S-NH_2 resin with Fmoc-Rink amide linker via **Method 2**, peptide **11** was synthesised by manual flow synthesis according to **Method 3** employing **sub-method 3.1** for the introduction of Cys(Trt) at position 5. The *N*-terminus was Boc protected via **Method 6**. N^{ε}-Dde was orthogonally removed from Lys^{18} via **Method 7** and the allenamide handle was coupled via **Method 9** to the side chain of residue 18. The final linear peptide was liberated from the resin and protecting groups simultaneously removed following **Method 10**, to afford the crude linear peptide as a slightly pink powder (300 mg, 83% yield [based on initial resin loading]). The crude linear peptide was cyclised via **Method 11** and quenched via **Method 12** to yield the crude desired peptide (**11**) as a pale-yellow powder at approximately 60% purity.

The crude peptide (69 mg), **11**, was dissolved in a solution of 1:9 MeCN/MQ H_2O at a concentration of ~7 mg/mL and purified (5 x ~2 mL injections) with a Phenomenex Luna C18 column (100 Å, 5 µm, 250 mm x 10 mm), employing a gradient with an isocratic phase of 5%B for 5 min, followed by a linear gradient to 50%B/min (*ca.* 1%B/min) at a flow rate of 5 mL/min. Fractions were analysed by RP-HPLC and ESI-MS for compound identification. Fractions from multiple runs were combined and lyophilised to afford the desired compound, **11**, as a white amorphous powder (8.0 mg, 11.6% recovery [based on crude yield], > 96% purity, 2.2% overall yield).

ESI-MS: Mass calculated for [$C_{106}H_{173}N_{39}O_{24}S$] 2409.3; deconvoluted observed: 2409.7 ± 0.2. Charge states; 483.0 $[M+5H]^{5+}$, 603.4 $[M+4H]^{4+}$, 804.2 $[M+3H]^{3+}$.

RP-HPLC: t_R = 24.5 min. Phenomenex Luna C18 (100Å, 5 µm, 4.6 mm x 250 mm), linear gradient 5%B to 45%B over 40 min (*ca.* 1%B/min) at 1 mL/min.

4 Spectral Data

4.1 RP-HPLC Data for Peptides 1, 3 – 11

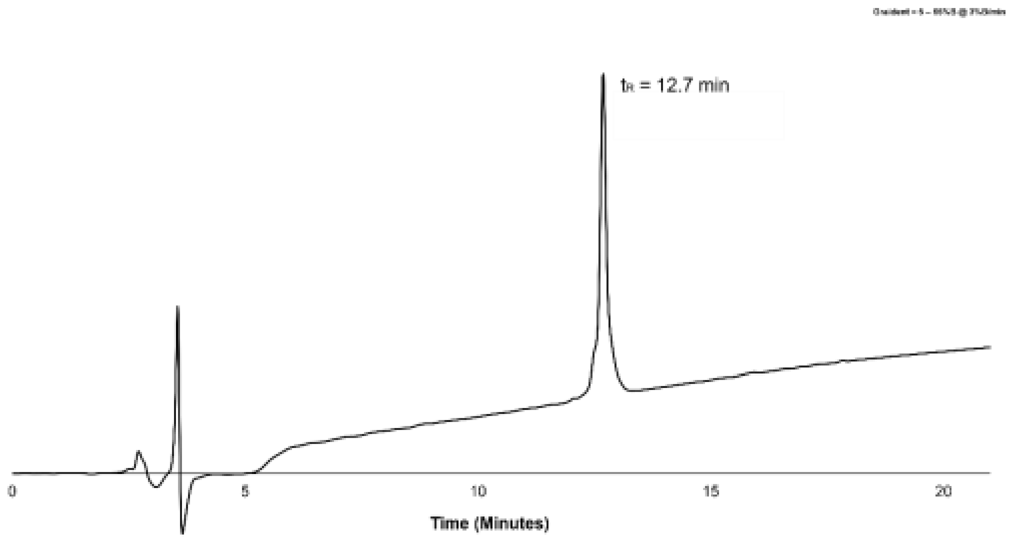

Supplementary Figure S1. **Analytical RP-HPLC** chromatogram (214 nm) of purified peptide, **1**, (ca. < 97% as analysed by peak area). Phenomenex Luna C18 (100Å, 5 µM, 4.6 mm x 250 mm), linear gradient 5%B to 65%B over 20 min (*ca.* 3%B/min) at 1 mL/min. t_R = 12.7 min.

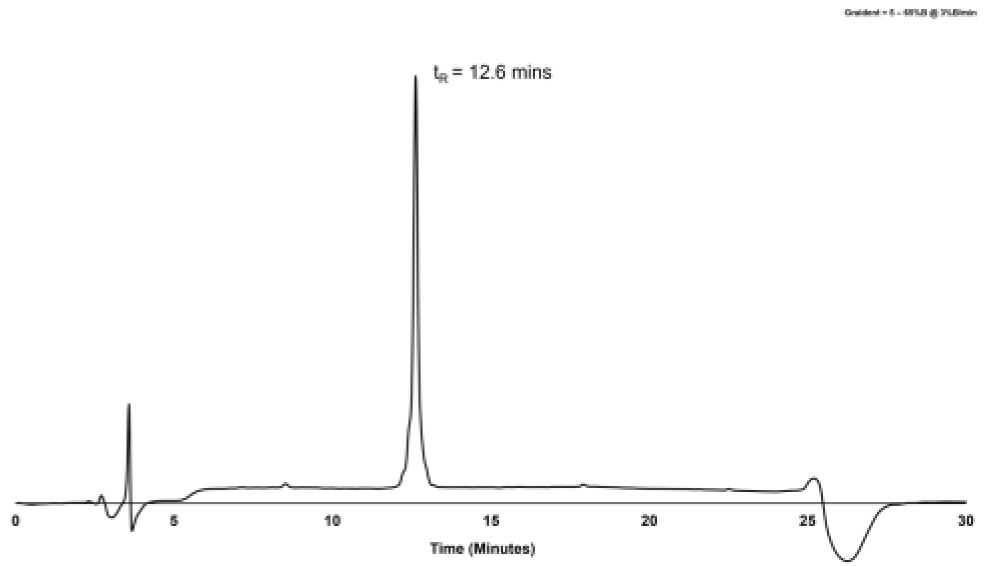

Supplementary Figure S2. **Analytical RP-HPLC** chromatogram (214 nm) of purified peptide, **3**, (ca. < 98% as analysed by peak area). Phenomenex Luna C18 (100Å, 5 µM, 4.6 mm x 250 mm), linear gradient 5%B to 65%B over 20 min (*ca.* 3%B/min) at 1 mL/min. t_R = 12.6 min.

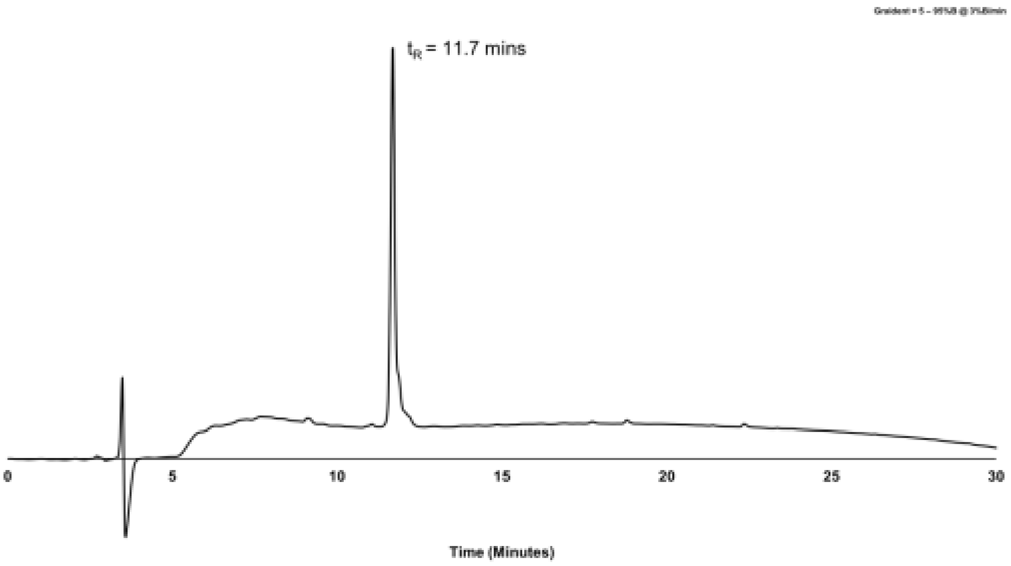

Supplementary Figure S3. **Analytical RP-HPLC** chromatogram (214 nm) of purified peptide, **4**, (ca. < 97% as analysed by peak area). Phenomenex Luna C18 (100Å, 5 µM, 4.6 mm x 250 mm), linear gradient 5%B to 65%B over 20 min (*ca.* 3%B/min) at 1 mL/min. t_R = 11.7 min.

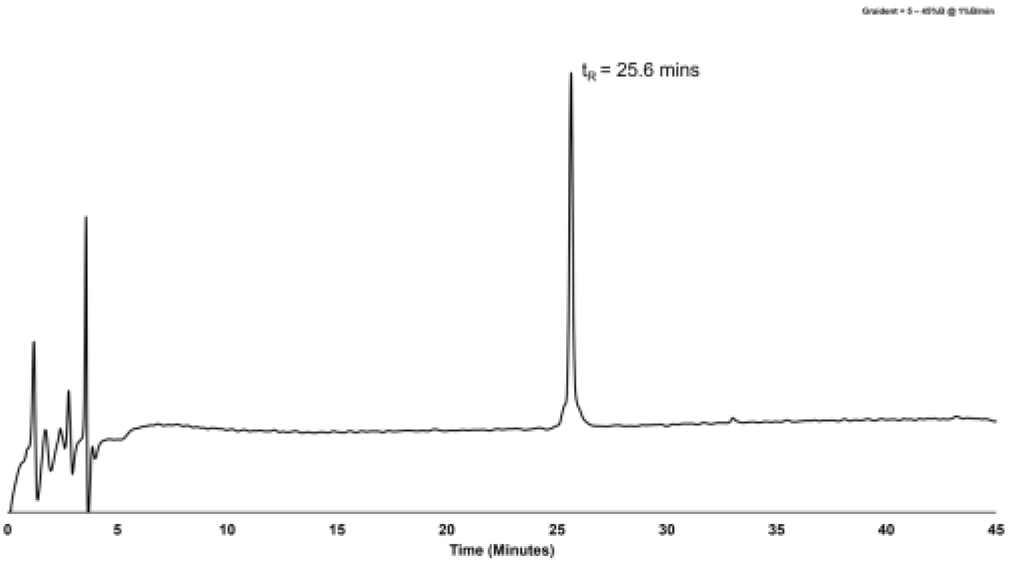

Supplementary Figure S4. **Analytical RP-HPLC** chromatogram (214 nm) of purified peptide, **5**, (ca. < 98% as analysed by peak area). Phenomenex Luna C18 (100Å, 5 µM, 4.6 mm x 250 mm), linear gradient 5%B to 45%B over 40 min (*ca.* 1%B/min) at 1 mL/min. t_R = 25.6 min.

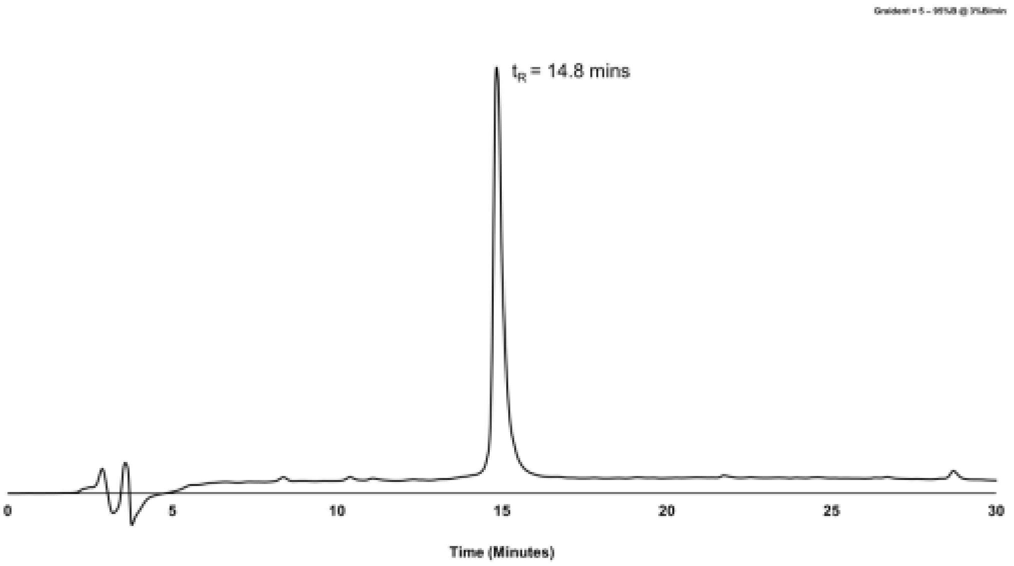

Supplementary Figure S5. **Analytical RP-HPLC** chromatogram (214 nm) of purified peptide, **6**, (ca. < 98% as analysed by peak area). Phenomenex Luna C18 (100Å, 5 µM, 4.6 mm x 250 mm), linear gradient 5%B to 65%B over 20 min (*ca.* 3%B/min) at 1 mL/min. t_R = 14.8 min.

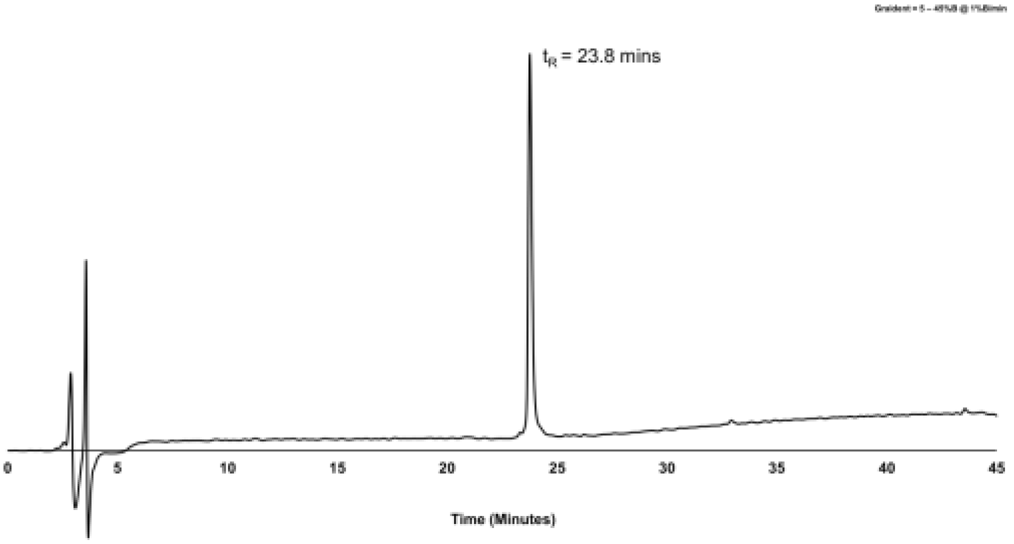

Supplementary Figure S6. **Analytical RP-HPLC** chromatogram (214 nm) of purified peptide, **7**, (ca. < 98% as analysed by peak area). Phenomenex Luna C18 (100Å, 5 µM, 4.6 mm x 250 mm), linear gradient 5%B to 45%B over 40 min (*ca.* 1%B/min) at 1 mL/min. t_R = 23.8 min.

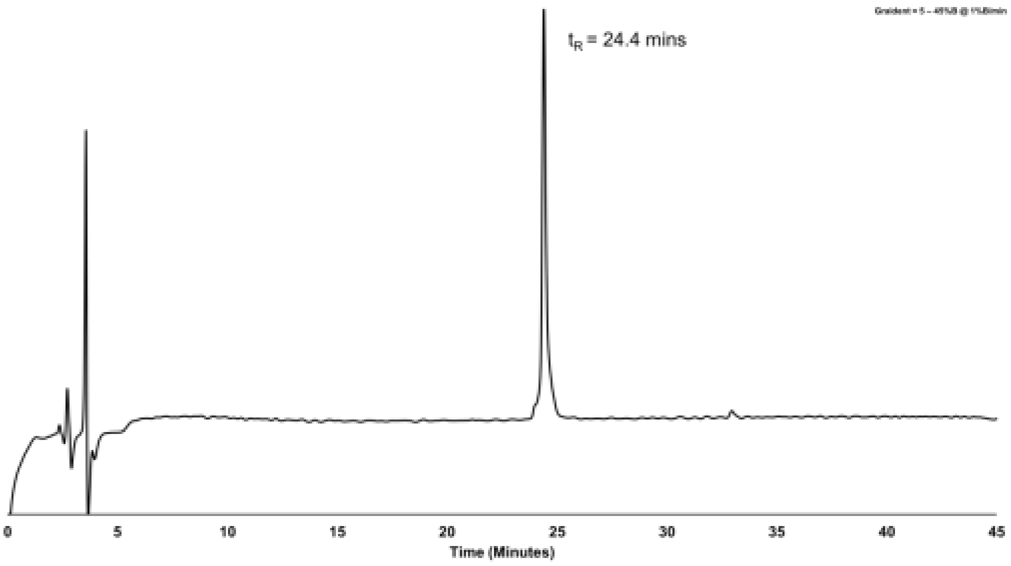

Supplementary Figure S7. **Analytical RP-HPLC** chromatogram (214 nm) of purified peptide, **8**, (ca. < 98% as analysed by peak area). Phenomenex Luna C18 (100Å, 5 µM, 4.6 mm x 250 mm), linear gradient 5%B to 45%B over 40 min (*ca.* 1%B/min) at 1 mL/min. t_R = 24.4 min.

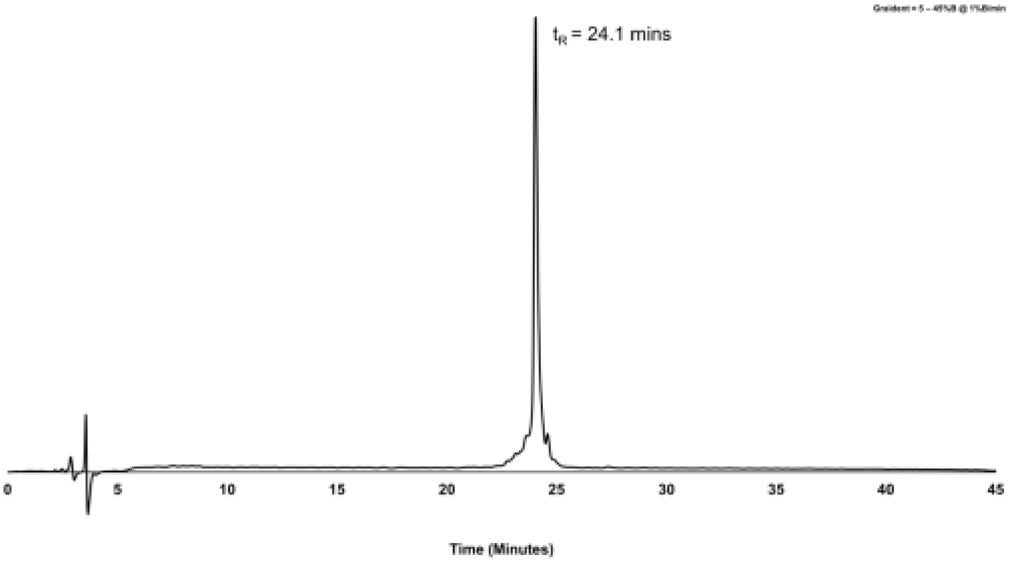

Supplementary Figure S8. **Analytical RP-HPLC** chromatogram (214 nm) of purified peptide, **9**, (ca. < 95% as analysed by peak area). Phenomenex Luna C18 (100Å, 5 µM, 4.6 mm x 250 mm), linear gradient 5%B to 45%B over 40 min (*ca.* 1%B/min) at 1 mL/min. t_R = 24.1 min.

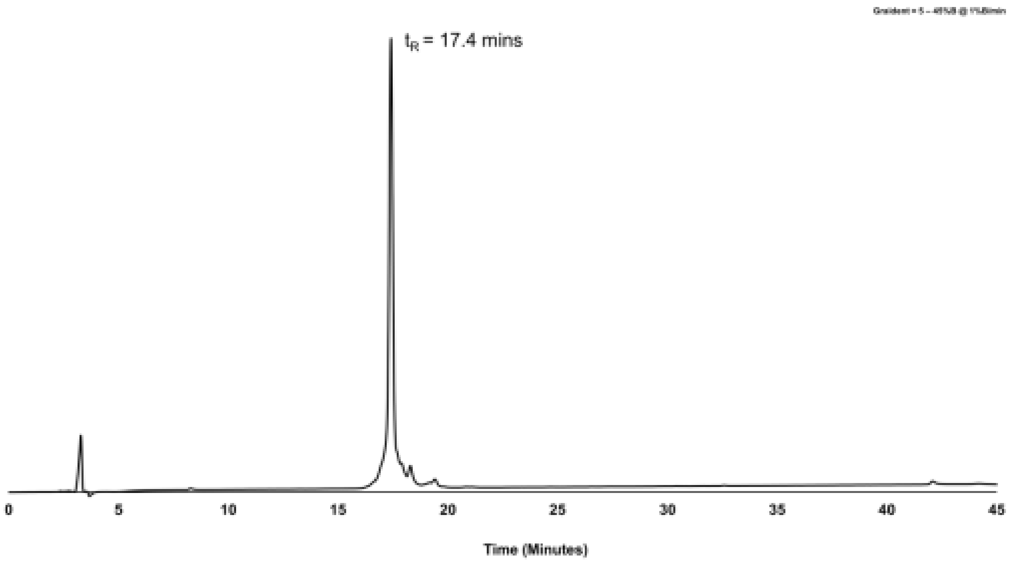

Supplementary Figure S9. **Analytical RP-HPLC** chromatogram (214 nm) of purified peptide, **10 (initial protocol)**, (ca. < 95% as analysed by peak area). Phenomenex Luna C18 (100Å, 5 µM, 4.6 mm x 250 mm), linear gradient 5%B to 45%B over 40 min (*ca.* 1%B/min) at 1 mL/min. t_R = 17.4 min.

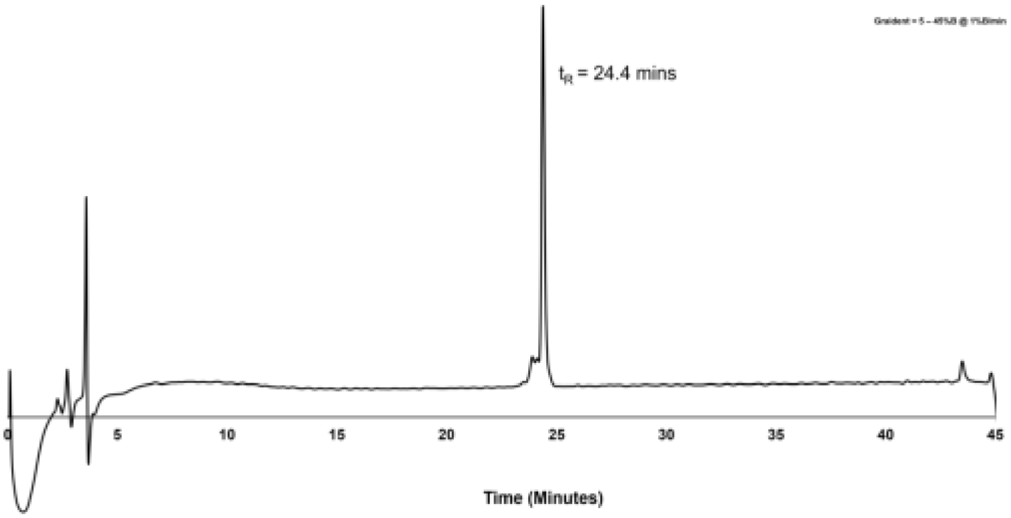

Supplementary Figure S10. **Analytical RP-HPLC** chromatogram (214 nm) of purified peptide, **10 (revised protocol)**, (ca. < 95% as analysed by peak area). Phenomenex Luna C18 (100Å, 5 µM, 4.6 mm x 250 mm), linear gradient 5%B to 45%B over 40 min (*ca.* 1%B/min) at 1 mL/min. t_R = 24.4 min.

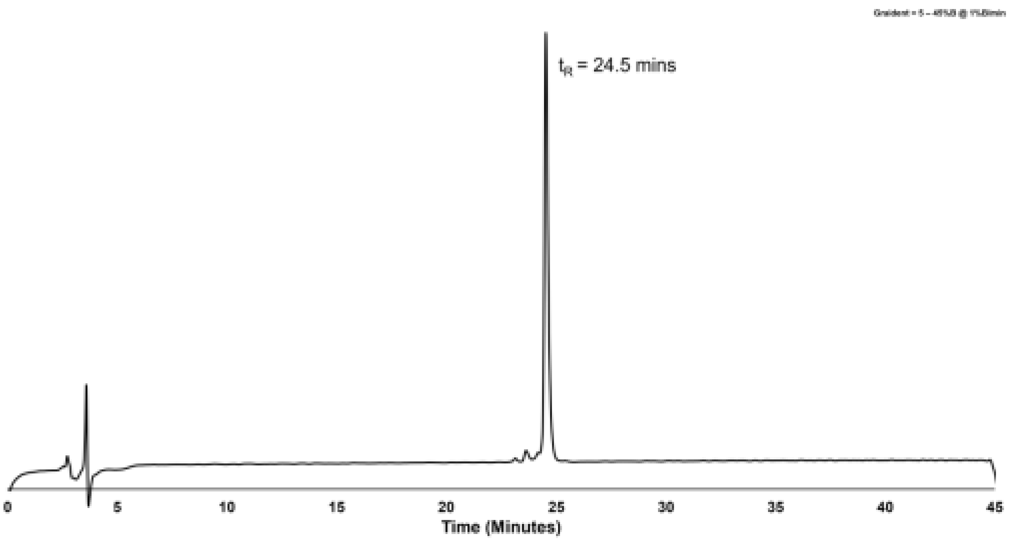

Supplementary Figure S11. **Analytical RP-HPLC** chromatogram (214 nm) of purified peptide, **11**, (ca. < 96% as analysed by peak area). Phenomenex Luna C18 (100Å, 5 µM, 4.6 mm x 250 mm), linear gradient 5%B to 45%B over 40 min (*ca.* 1%B/min) at 1 mL/min. t_R = 24.5 min.

4.2 ESI-MS Data for Peptides 1, 3 – 11

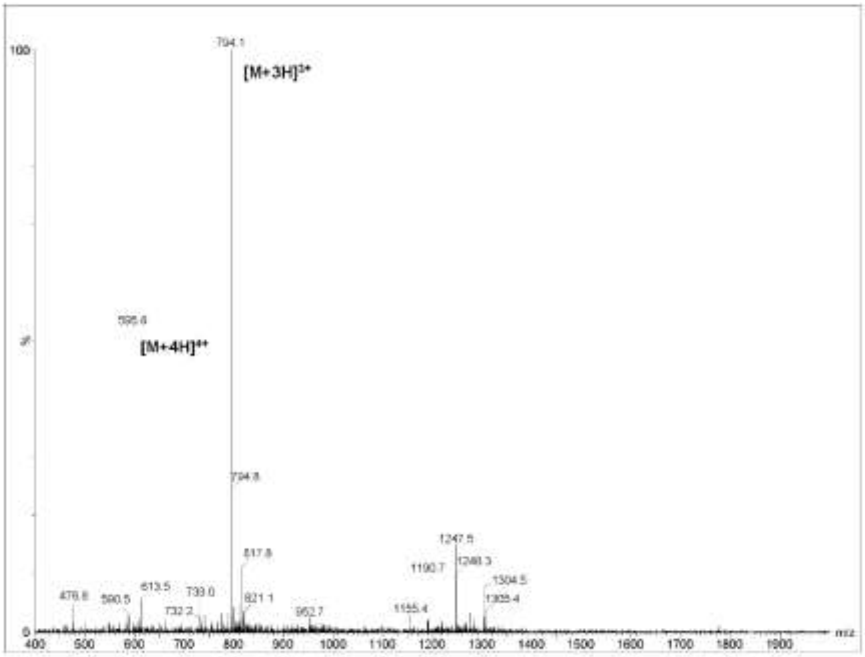

Supplementary Figure S12. **ESI-MS** of purified peptide, capitellacin (**1**), *m/z* calculated for [$C_{99}H_{159}N_{37}O_{24}S_4$] 2379.1; deconvoluted observed: 2379.1 ± 0.2. Charge states; 595.8 $[M+4H]^{4+}$, 794.1 $[M+3H]^{3+}$.

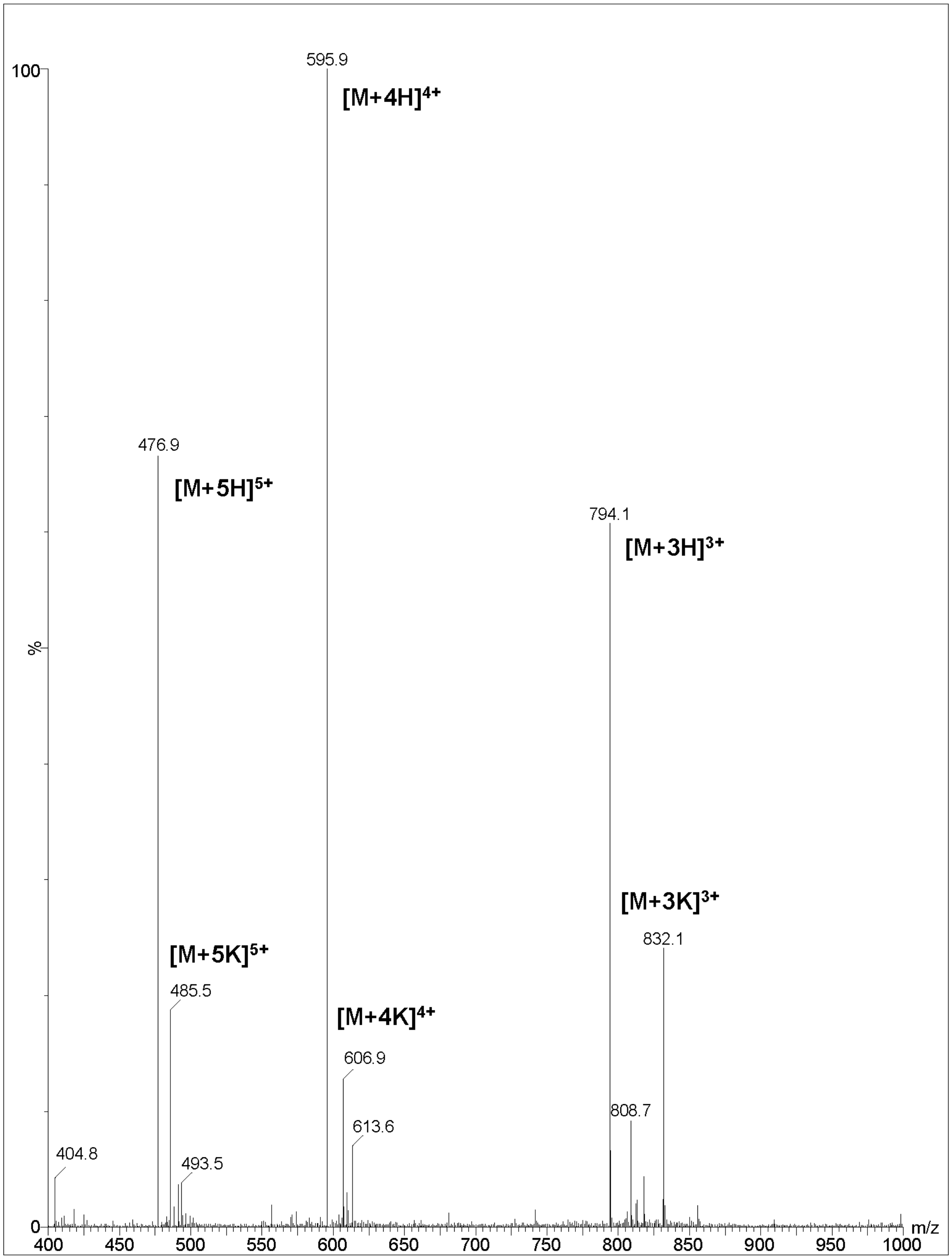

Supplementary Figure S13. **ESI-MS** of purified peptide, **3**, *m/z* calculated for [$C_{99}H_{159}N_{37}O_{24}S_4$] 2379.1; deconvoluted observed: 2379.1 ± 0.2. Charge states; 476.9 $[M+5H]^{5+}$, 595.9 $[M+4H]^{4+}$, 794.1 $[M+3H]^{3+}$.

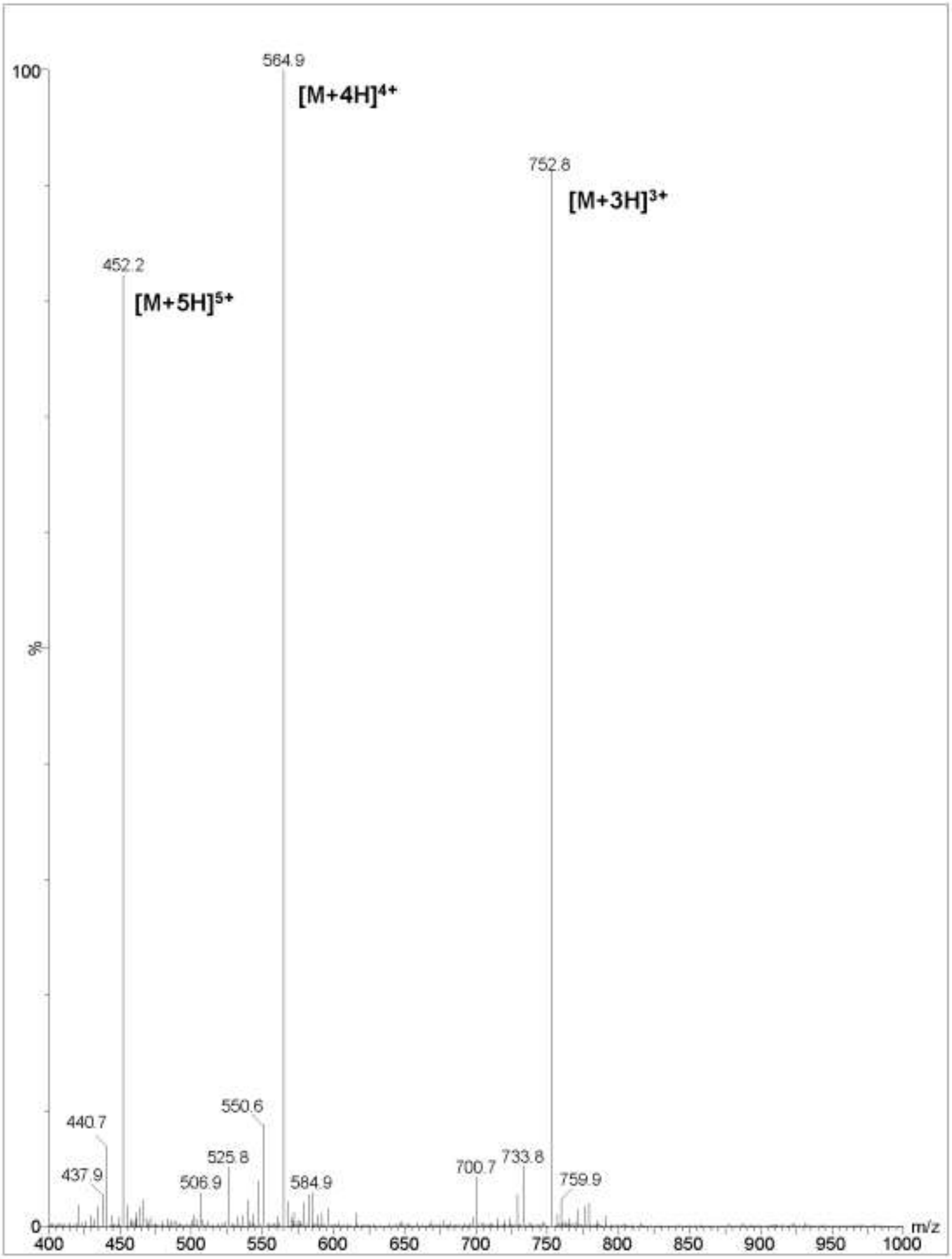

Supplementary Figure S14. **ESI-MS** of purified peptide, **4**, *m/z* calculated for [$C_{99}H_{163}N_{37}O_{24}$] 2255.2; deconvoluted observed: 2255.7 ± 0.3. Charge states; 452.2 [M+5H]$^{5+}$, 564.9 [M+4H]$^{4+}$, 752.8 [M+3H]$^{3+}$.

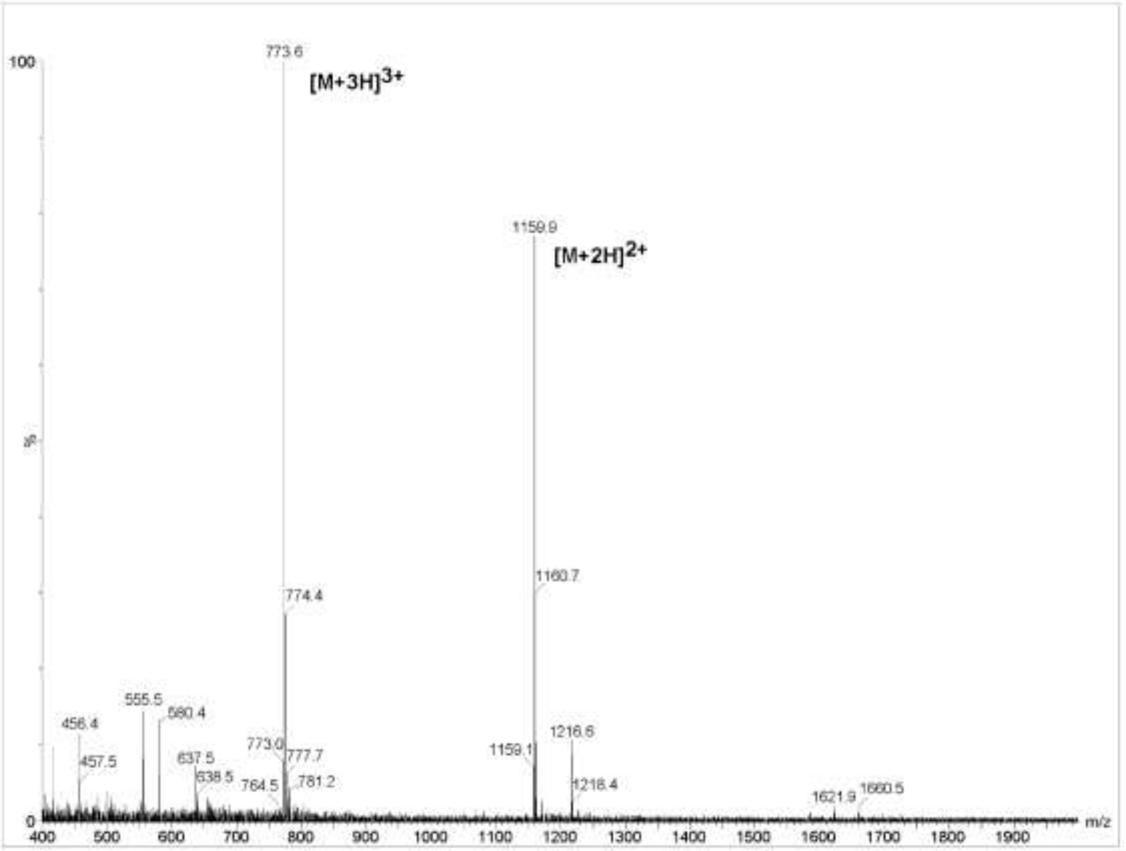

Supplementary Figure S15. **ESI-MS** of purified peptide, **5**, *m/z* calculated for [$C_{99}H_{161}N_{37}O_{24}S_2$] 2317.2; deconvoluted observed: 2317.1 ± 0.1. Charge states; 773.6 $[M+3H]^{3+}$,1159.9 $[M+2H]^{2+}$.

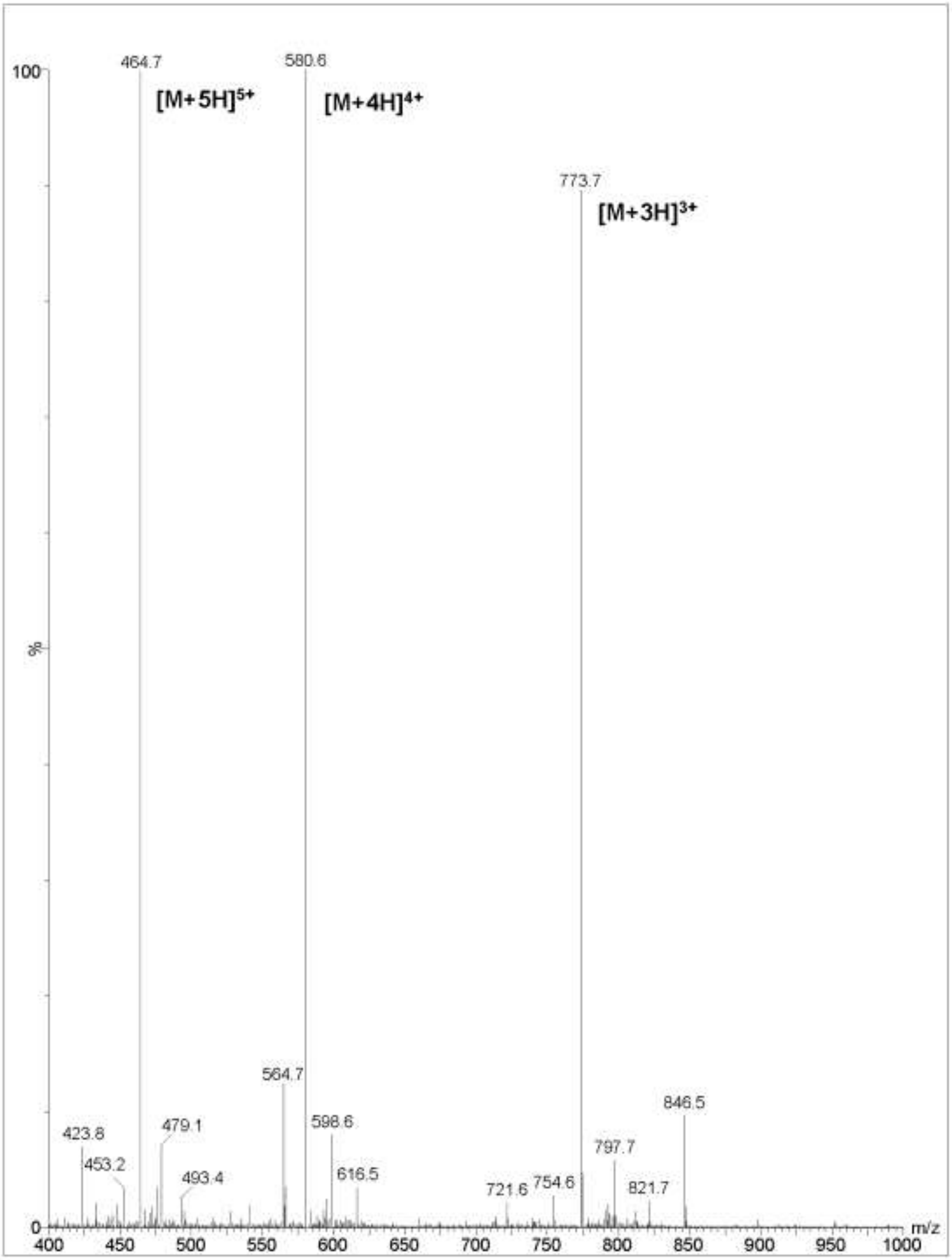

Supplementary Figure S16. **ESI-MS** of purified peptide, **6**, *m/z* calculated for [$C_{99}H_{161}N_{37}O_{24}S_2$] 2317.2; deconvoluted observed: 2317.2 ± 0.1. Charge states; 464.7 $[M+5H]^{5+}$, 580.6 $[M+4H]^{4+}$, 773.7 $[M+3H]^{3+}$.

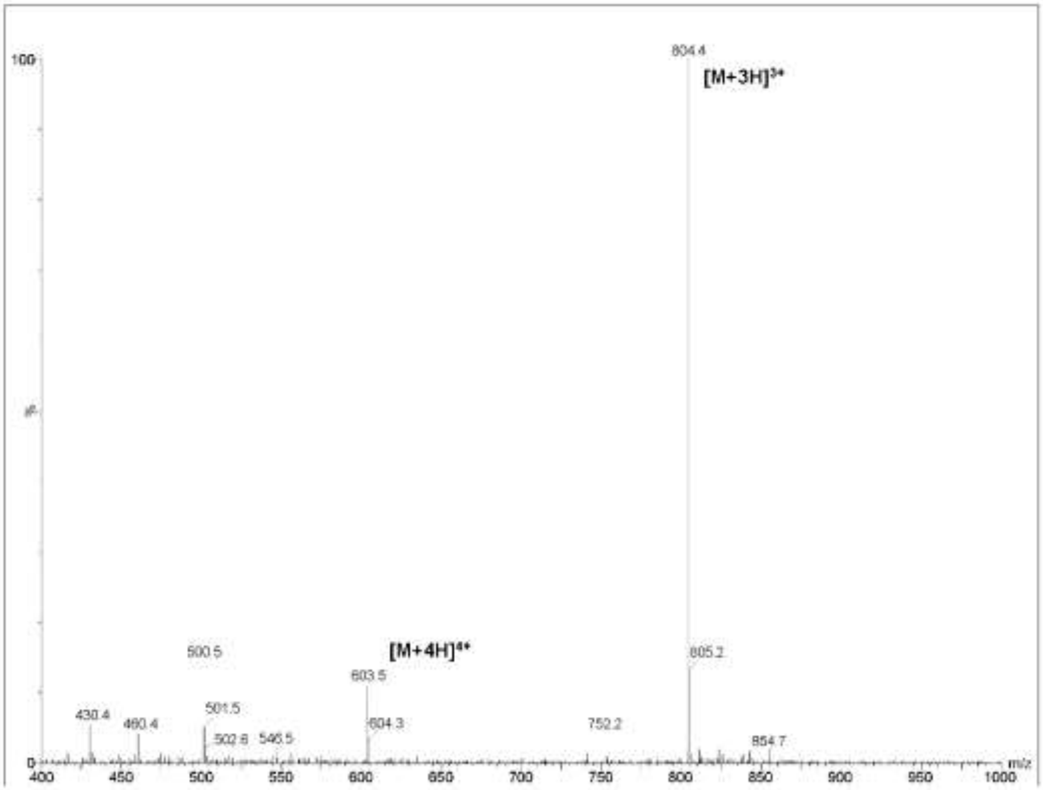

Supplementary Figure S17. **ESI-MS** of purified peptide, **7**, mass calculated for [$C_{106}H_{173}N_{39}O_{24}S$] 2409.3; deconvoluted observed: 2410.1 ± 0.1. Charge states; 603.5 $[M+4H]^{4+}$, 804.4 $[M+3H]^{3+}$.

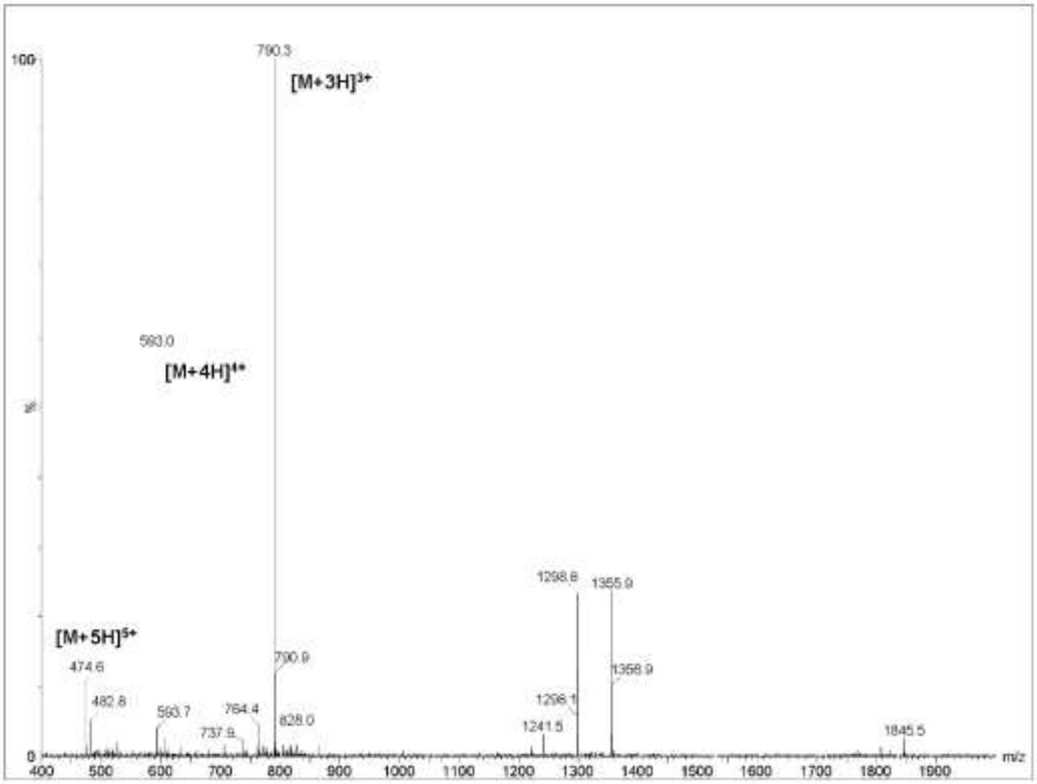

Supplementary Figure S18. **ESI-MS** of purified peptide, **8**, mass calculated for [$C_{103}H_{167}N_{39}O_{24}S$] 2367.3; deconvoluted observed: 2368.1 ± 0.1. Charge states; 474.6 $[M+5H]^{5+}$, 593.0 $[M+4H]^{4+}$, 790.3 $[M+3H]^{3+}$.

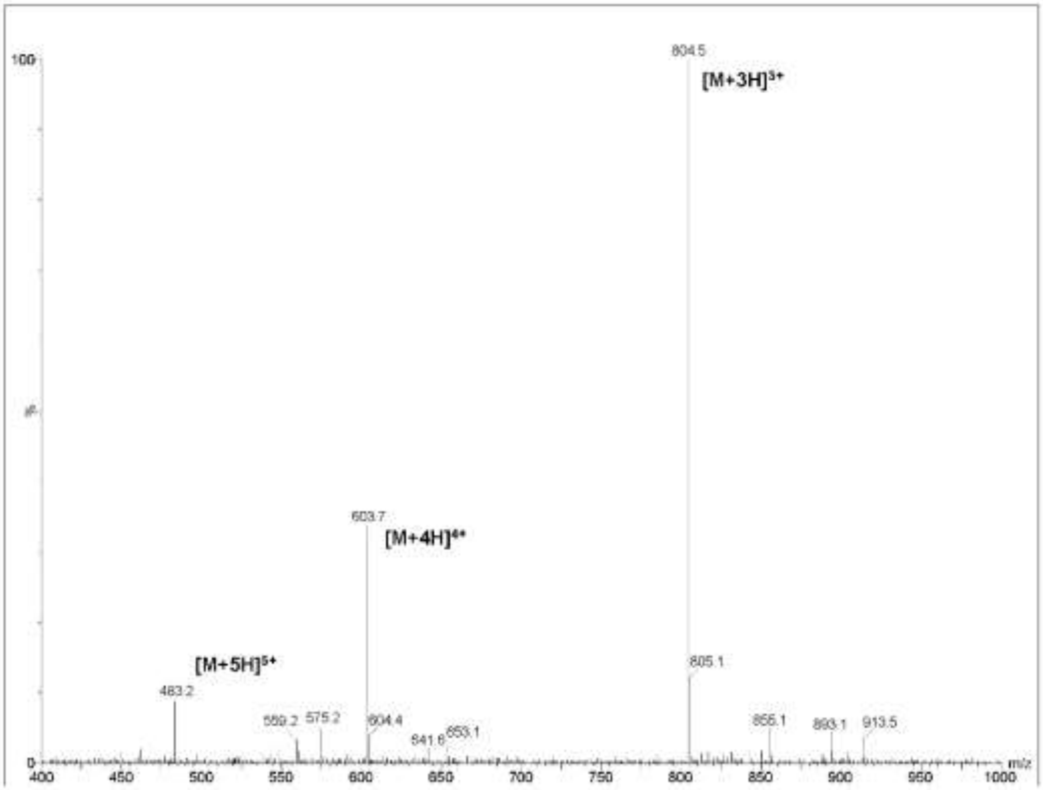

Supplementary Figure S19. **ESI-MS** of purified peptide, **9**, mass calculated for [$C_{106}H_{173}N_{39}O_{24}S$] 2409.3; deconvoluted observed: 2410.7 ± 0.3. Charge states; 483.2 [$M+5H]^{5+}$, 603.7 [$M+4H]^{4+}$, 804.5 [$M+3H]^{3+}$.

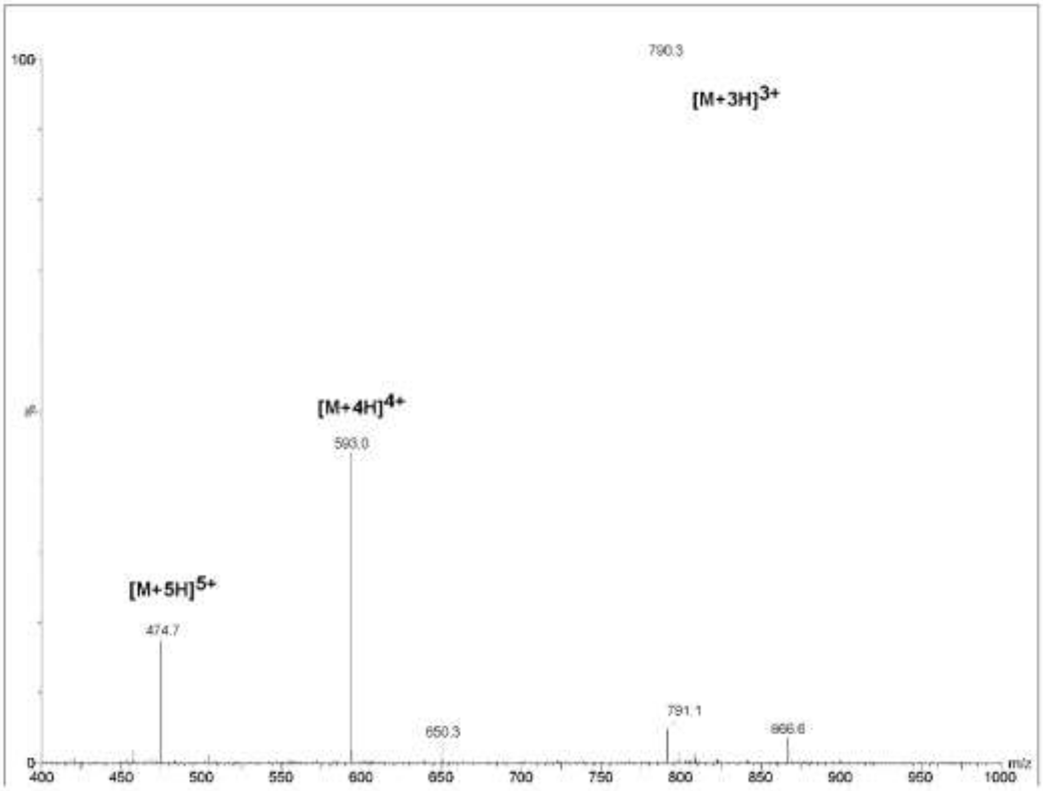

Supplementary Figure S20. **ESI-MS** of purified peptide, **10 (revised protocol)**, mass calculated for [$C_{103}H_{167}N_{39}O_{24}S$] 2367.3; deconvoluted observed: 2368.1 ± 0.3. Charge states; 474.7 $[M+5H]^{5+}$, 593.0 $[M+4H]^{4+}$, 790.3 $[M+3H]^{3+}$.

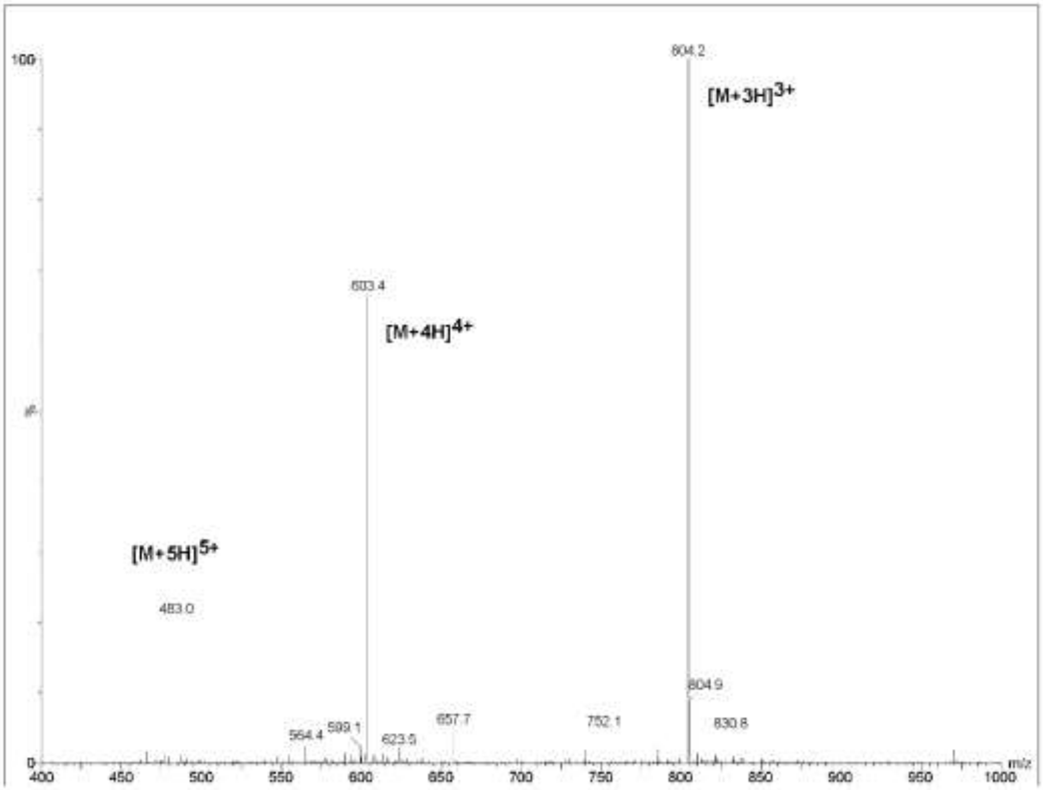

Supplementary Figure S21. **ESI-MS** of purified peptide, **11**, mass calculated for [$C_{106}H_{173}N_{39}O_{24}S$] 2409.3; deconvoluted observed: 2409.7 ± 0.2. Charge states; 483.0 $[M+5H]^{5+}$, 603.4 $[M+4H]^{4+}$, 804.2 $[M+3H]^{3+}$.

4.3 HRMS of Analogues 1, 3 – 6

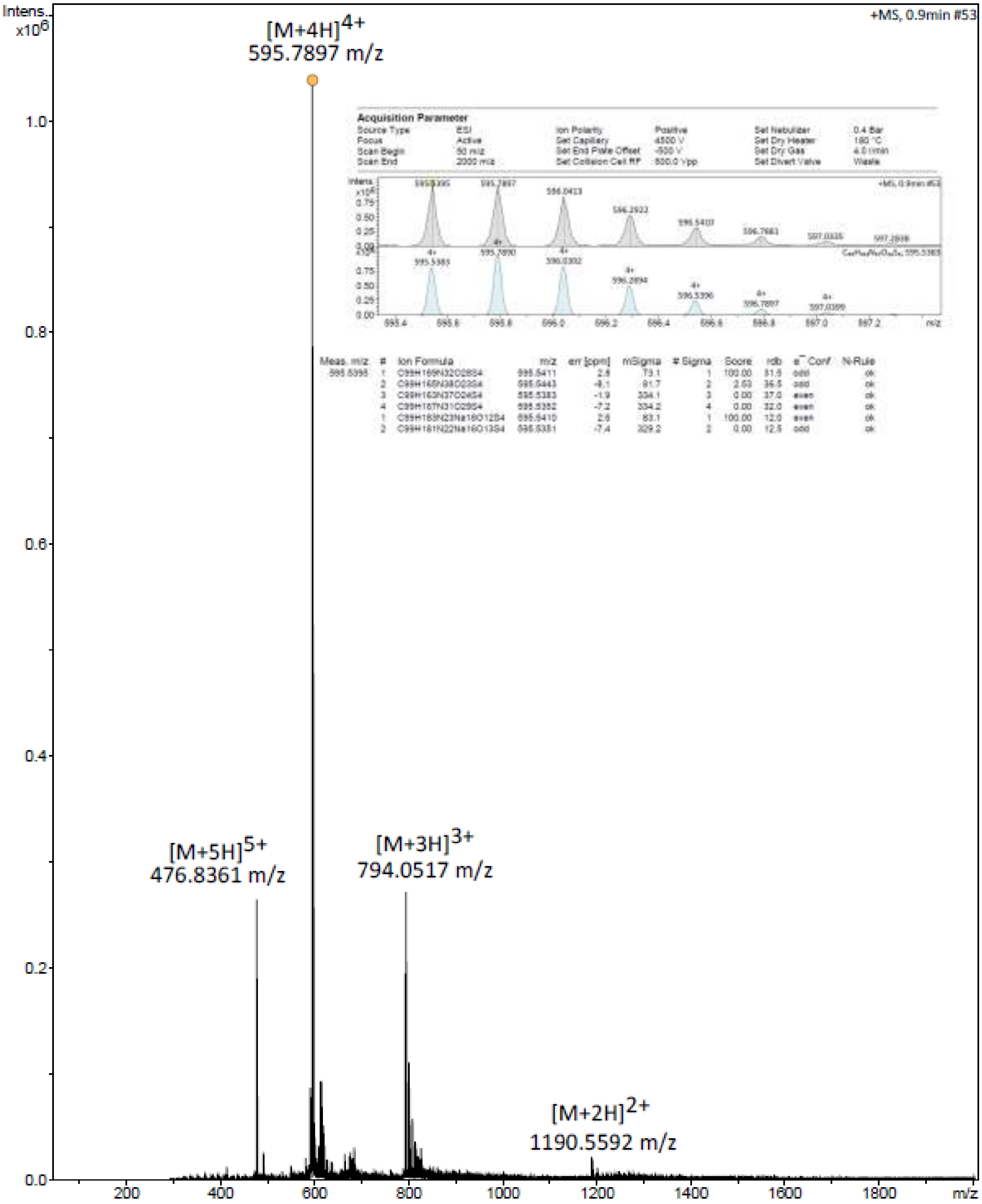

Supplementary Figure S22. **HRMS (ESI/Q-TOF),** deconvoluted *m/z* calculated for purified capitellacin (**1**), [$C_{99}H_{159}N_{37}O_{24}S_4$]: 2379.1275; observed: 2379.1532 ± 0.0258.

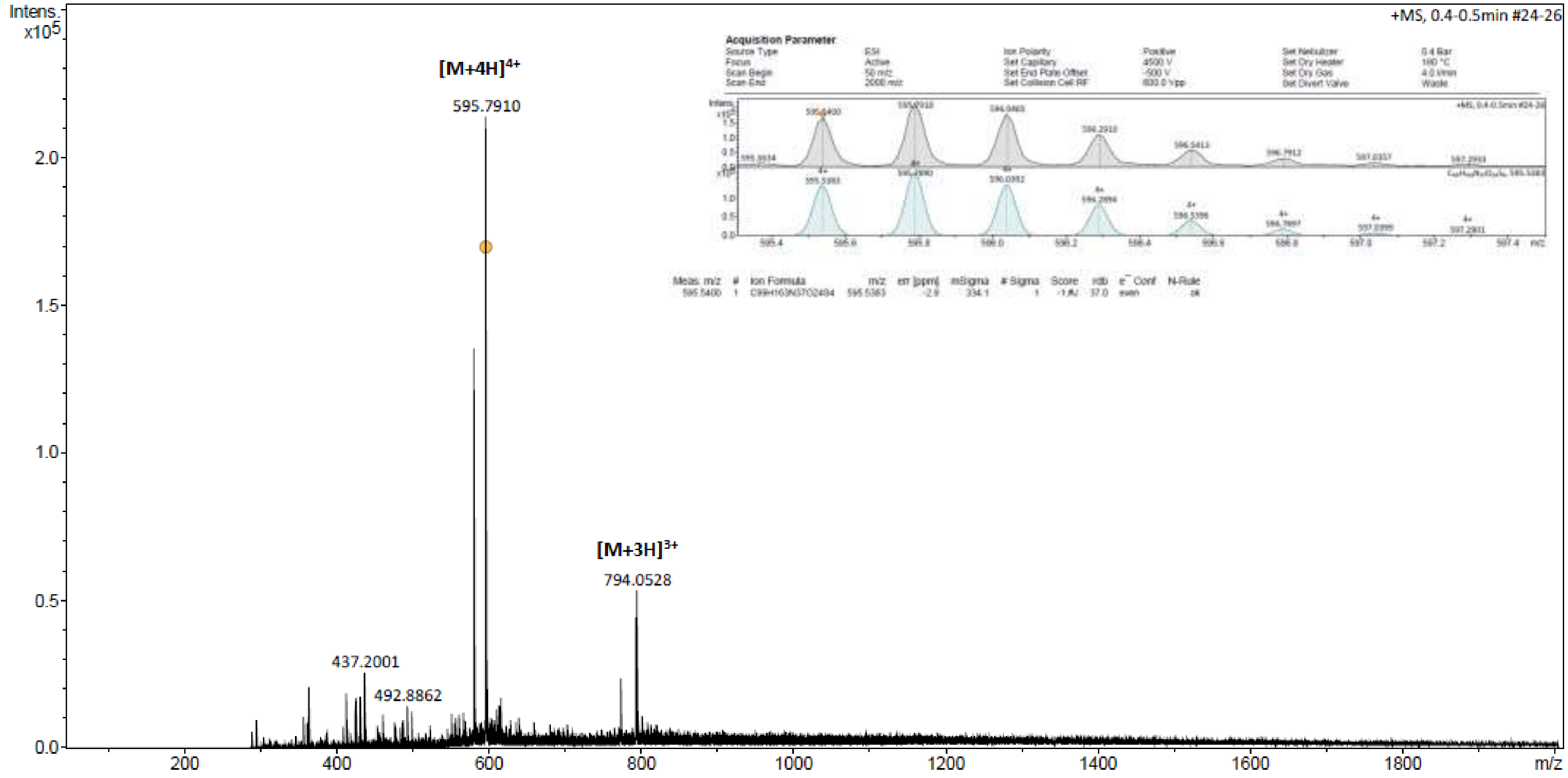

Supplementary Figure S23. **HRMS (ESI/Q-TOF),** deconvoluted mass calculated for purified peptide **3**, [$C_{99}H_{159}N_{37}O_{24}S_4$]: 2379.1275; observed: 2379.1612 ± 0.0040.

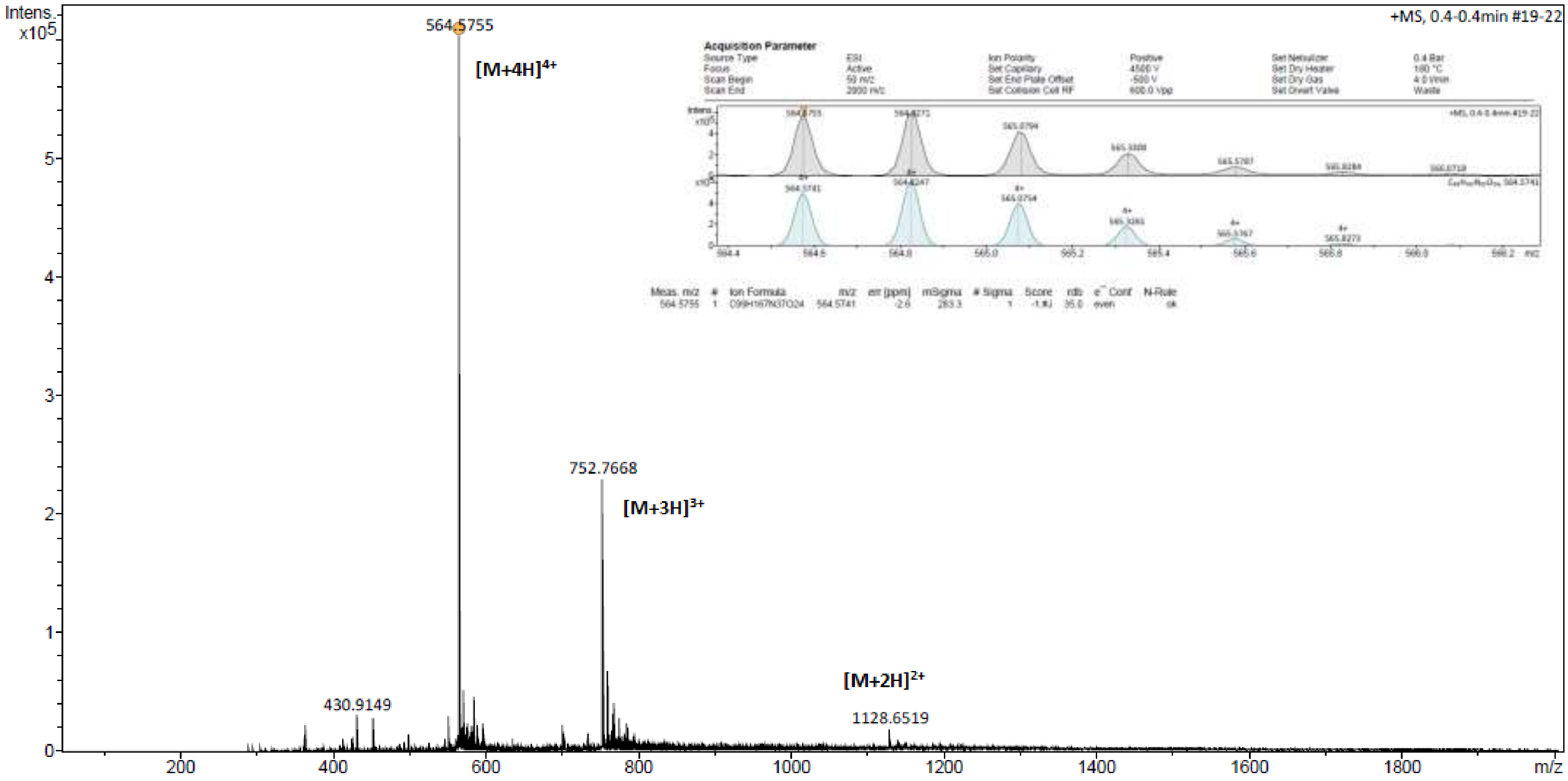

Supplementary Figure S24. **HRMS (ESI/Q-TOF),** deconvoluted mass calculated for purified peptide **4**, [$C_{99}H_{163}N_{37}O_{24}$]: 2255.2705; observed: 2254.9687 ± 0.5774.

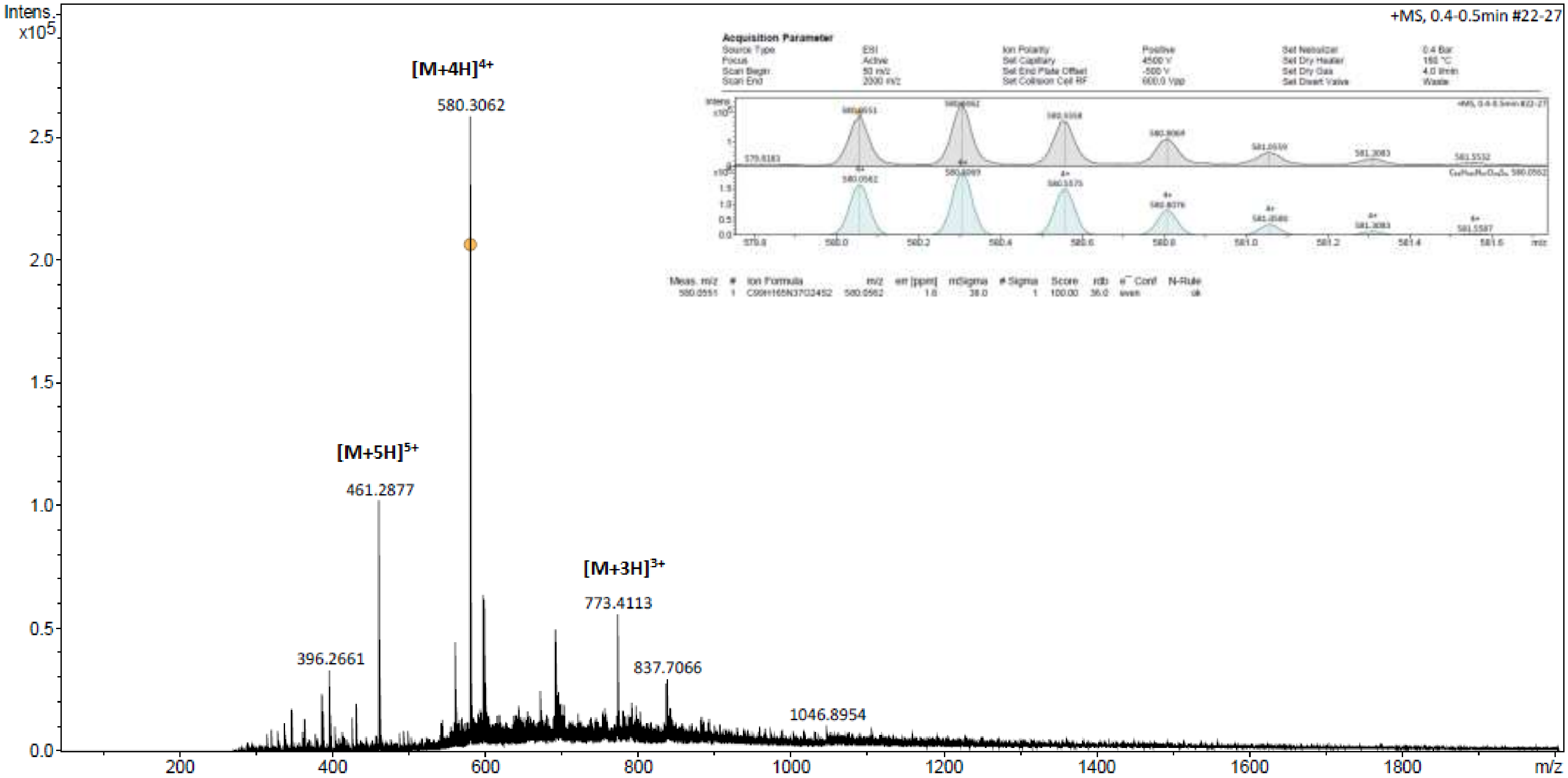

Supplementary Figure S25. **HRMS (ESI/Q-TOF),** deconvoluted mass calculated for purified peptide **5**, [$C_{99}H_{161}N_{37}O_{24}S_2$]: 2317.1990; observed: 2317.2294 ± 0.0064.

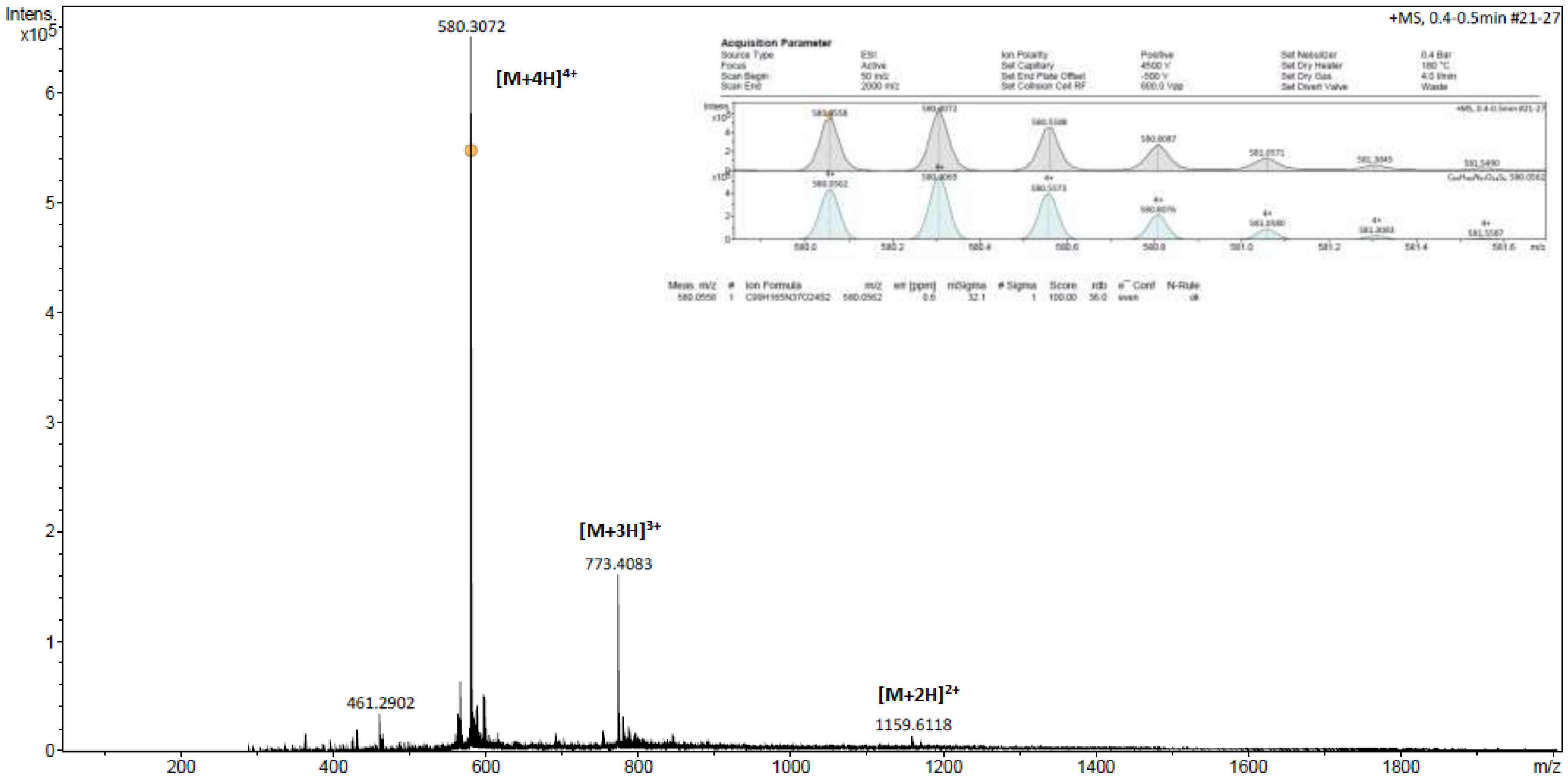

Supplementary Figure S26. **HRMS (ESI/Q-TOF),** deconvoluted mass calculated for purified peptide **6**, [$C_{99}H_{161}N_{37}O_{24}S_2$]: 2317.1990; observed: 2317.2258 ± 0.0027.

4.4 ^{1}H NMR Data for Analogues 5, 7 – 11

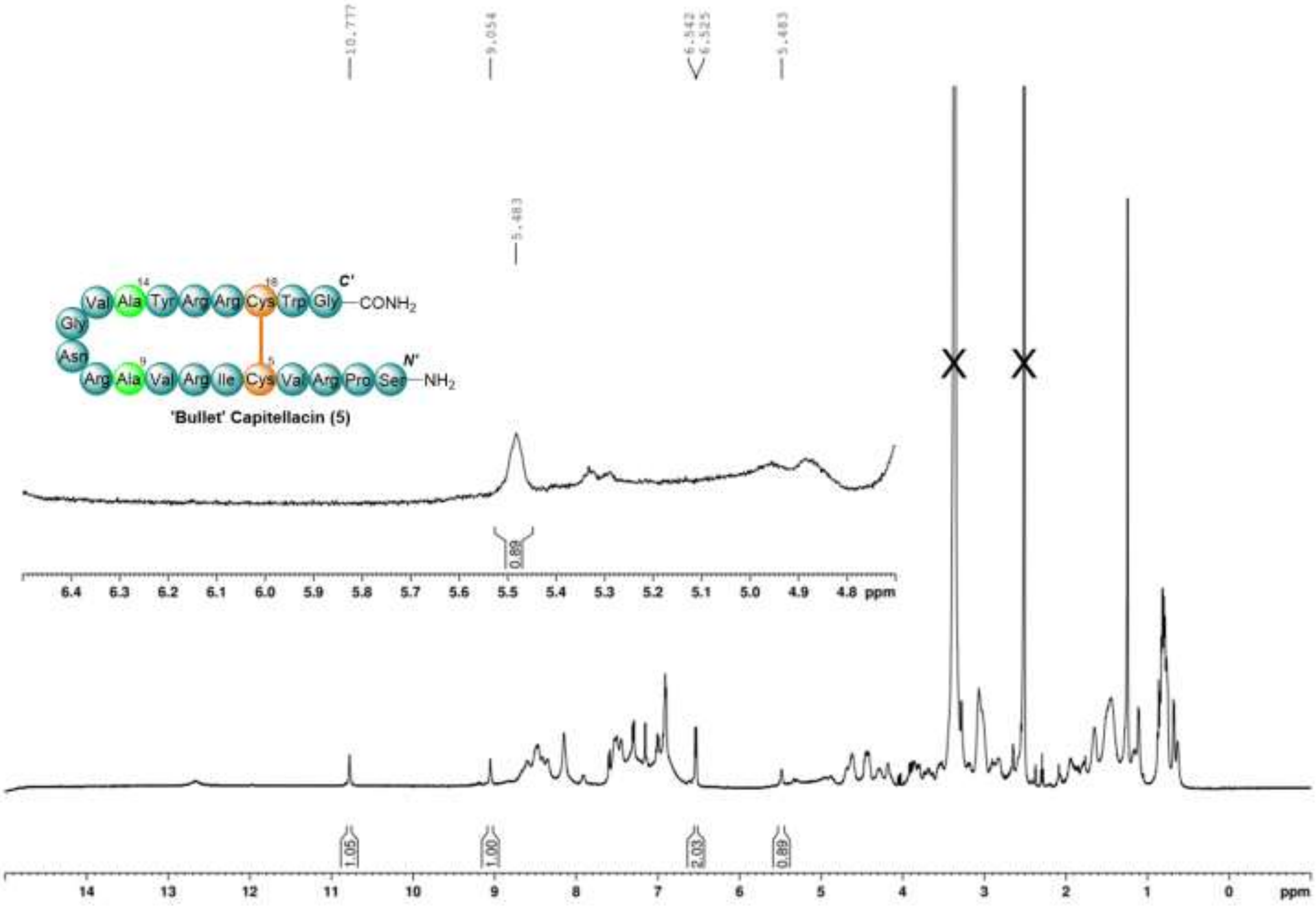

Supplementary Figure S27. **^{1}H NMR** of 'bullet' capitellacin, **5**, in DMSO-$_{d6}$, vinyl proton region inset. X's denote H_2O (δ 3.33 ppm) and DMSO (δ 2.50 ppm) residual peaks. Cys residues and disulphide (orange) and Ala residues (bright green).

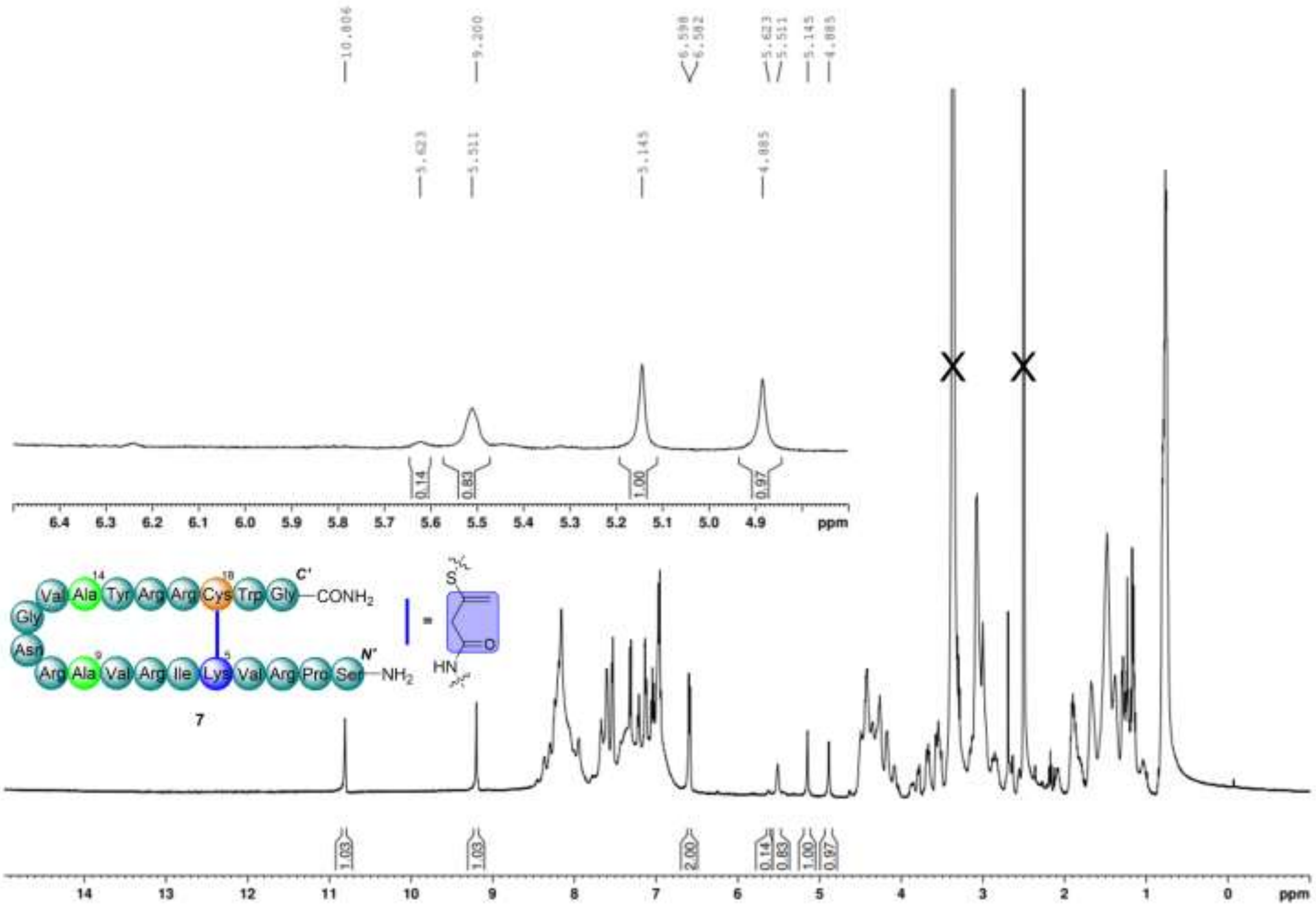

Supplementary Figure S28. ^{1}**H NMR** (500 MHz, 298 K, DMSO-$_{d6}$) spectra of **7** with vinyl region zoom inset. X denotes H_2O (δ 3.33 ppm) and DMSO (δ 2.50 ppm) residual peaks. Spectrum referenced to an internal standard of DMSO at 2.50 ppm. Cys residues (orange), Ala residues (bright green) and substituted Cys residue and vinyl sulphide bridge (blue).

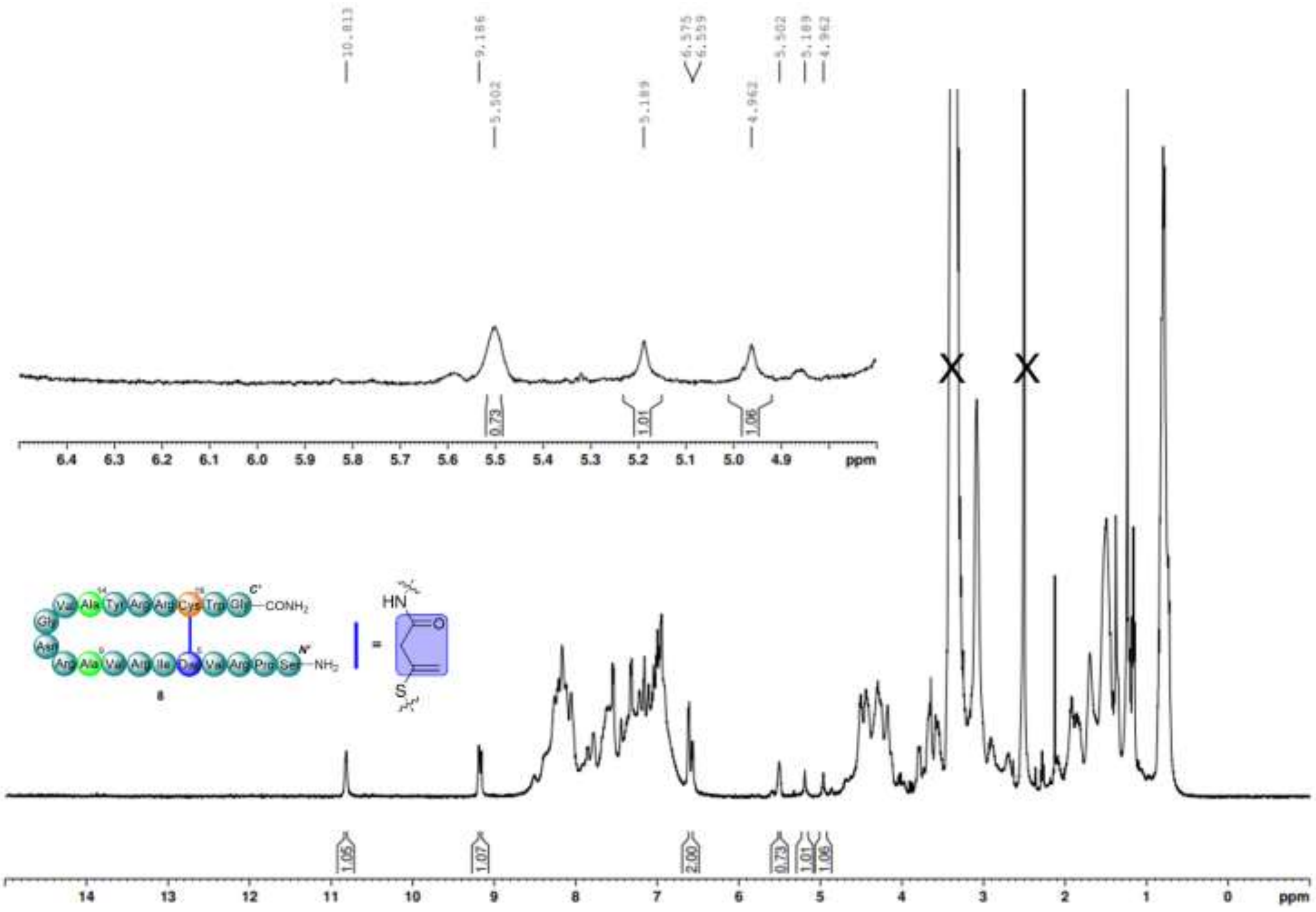

Supplementary Figure S29. **¹H NMR** (500 MHz, 298 K, DMSO-$_{d6}$) spectra of **8** with vinyl region zoom inset. X denotes H_2O (δ 3.33 ppm) and DMSO (δ 2.50 ppm) residual peaks. Spectrum referenced to an internal standard of DMSO at 2.50 ppm. Cys residues (orange), Ala residues (bright green) and substituted Cys residue and vinyl sulphide bridge (blue).

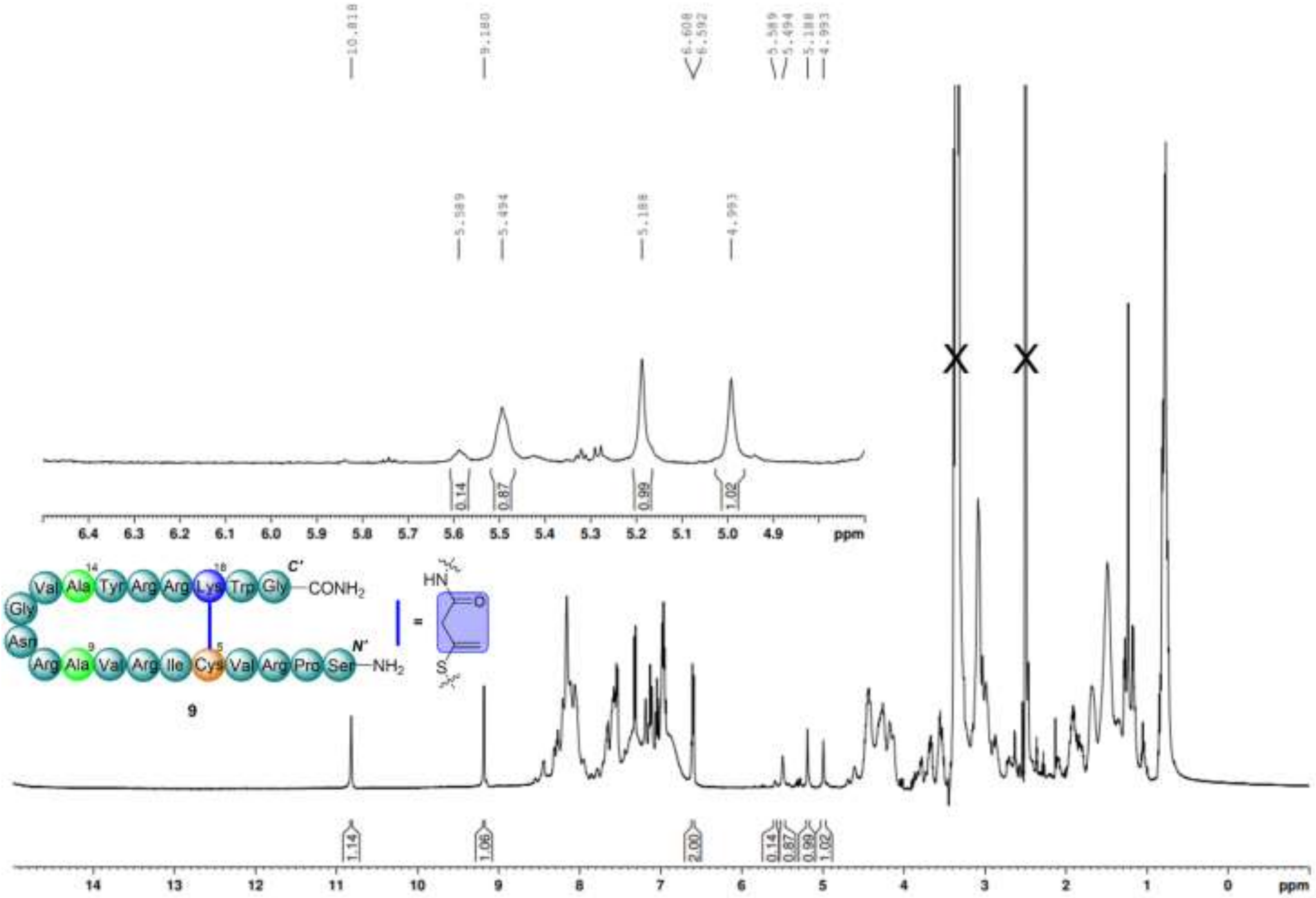

Supplementary Figure S30. **^{1}H NMR** (500 MHz, 298 K, DMSO-$_{d6}$) spectra of **9** with vinyl region zoom inset. X denotes H_2O (δ 3.33 ppm) and DMSO (δ 2.50 ppm) residual peaks. Spectrum referenced to an internal standard of DMSO at 2.50 ppm. Cys residues (orange), Ala residues (bright green) and substituted Cys residue and vinyl sulphide bridge (blue).

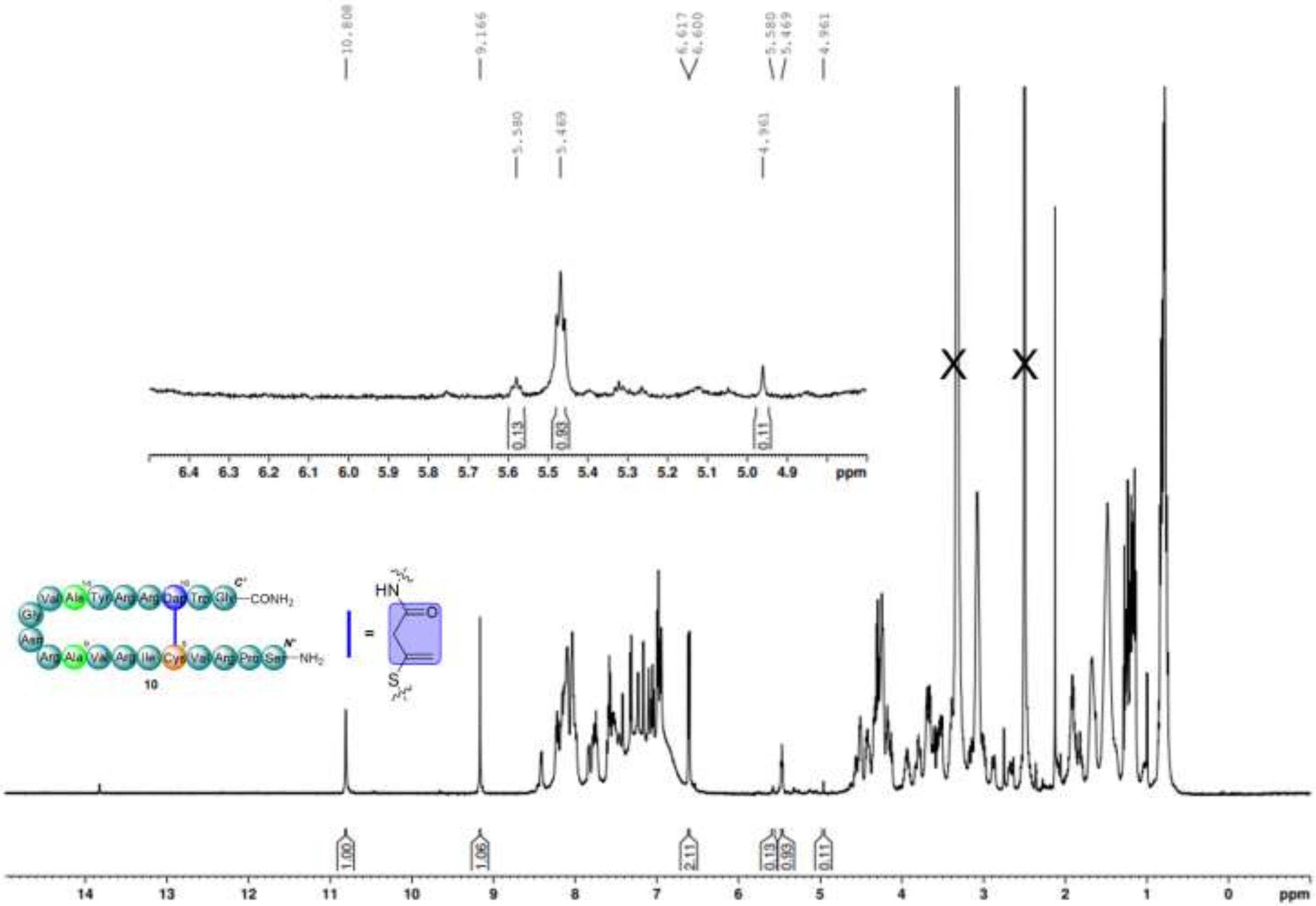

Supplementary Figure S31. **^{1}H NMR** (500 MHz, 298 K, DMSO-$_{d6}$) spectra of **10** with vinyl region zoom inset. X denotes H_2O (δ 3.33 ppm) and DMSO (δ 2.50 ppm) residual peaks. Spectrum referenced to an internal standard of DMSO at 2.50 ppm. Cys residues (orange), Ala residues (bright green) and substituted Cys residue and vinyl sulphide bridge (blue).

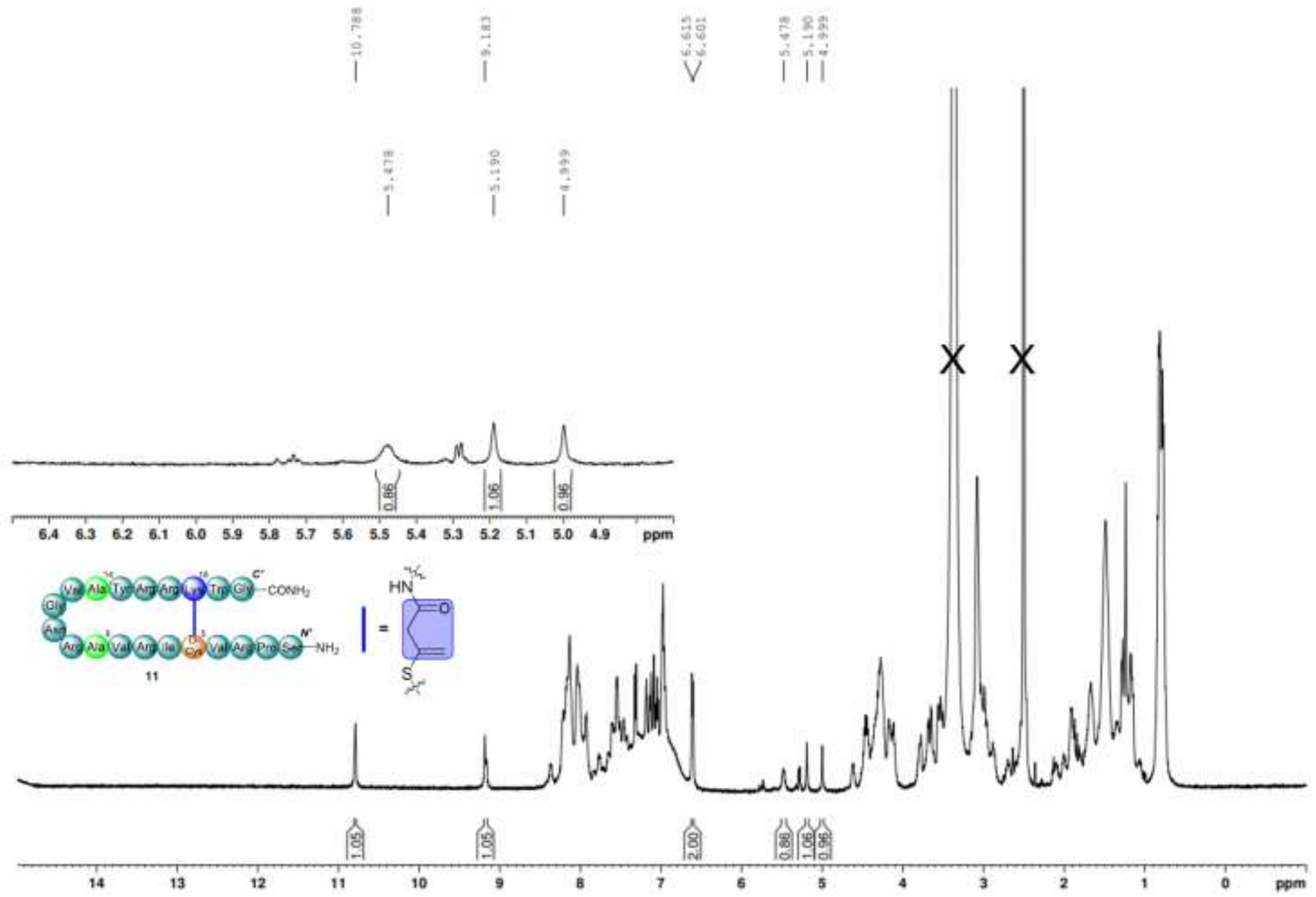

Supplementary Figure S32. ^{1}**H NMR** (500 MHz, 298 K, DMSO-$_{d6}$) spectra of **11** with vinyl region zoom inset. X denotes H_2O (δ 3.33 ppm) and DMSO (δ 2.50 ppm) residual peaks. Spectrum referenced to an internal standard of DMSO at 2.50 ppm. Cys residues (orange), Ala residues (bright green) and substituted Cys residue and vinyl sulphide bridge (blue).

5 Antimicrobial Testing of Capitellacin (1) and Analogues 3 – 11

5.1 Antibacterial Testing

Staphylococcus aureus ATCC 29213, *Pseudomonas aeruginosa* (SVB-B9) (type strain) and *Escherichia coli* ATCC 25922 were grown in cation adjusted Mueller Hinton (CA-MHB) broth at 37 ˚C with shaking (200 rpm). MIC assays were performed in accordance with the CLSI recommended protocol [4]. Briefly**,** a two-fold dilution series of the test compounds (from 64 mM to 0.25 mM, final) was prepared in triplicate in polypropylene 96-well plates in CAMHB. Fresh cultures of bacteria grown for *ca. 6* h were diluted accordingly in fresh media before adding 50 µL of inoculum to each well of the MIC plate, to achieve a final volume of 100 µL with a uniform CFU/ml of ~5 x10^5 in each well. A growth control (untreated) and sterility control (non-inoculated) well was included for each test compound replicate. Plates were incubated at 37 °C with shaking for 18 h before determining the MIC. MIC values were determined as the lowest concentration at which no growth was observed consistently across all three biological replicates of the assay and within triplicates of each test compound.

5.2 Antifungal Testing

Antimicrobial susceptibility of *Candida albicans* SC5314 (type strain) was assessed by broth microdilution in accordance with the CLSI recommended protocols. Briefly, a two-fold dilution series of the test compounds (from 64 µM to 0.25 µM, final) was prepared in triplicate in polypropylene 96-well plates, using RPMI 1640 media (with glutamine and phenol red, without bicarbonate). Inoculum was prepared from a 24 h old culture by picking 5 colonies (~1 mm in diameter) and resuspending these in 0.85% saline. Upon vortex mixing, the resulting suspension was diluted to provide a solution with absorbance equivalent to that of a 0.5 McFarland standard, approx. 0.1 OD at 530 nm (using 1 cm path length cuvette). This suspension was then diluted 1:50 and a further 1:20 with RPMI 1640 media to as to achieve a final inoculum of 0.5 x 10^3 to 2.5 x 10^3 CFU/mL upon addition to the plate. The plates were incubated at 35 °C for 48 h at which time the MIC was determined as the lowest test concentration at which no turbidity was observed across all three replicates. The assay was performed independently on three occasions and the reported MIC defined as the lowest concentration in which agreement was observed for all three biological replicates. [5]

6 Circular Dichroism of Capitellacin (1) and Analogues 3 - 11

All CD spectra were recorded using a Chirascan CD spectrometer (Applied Photophysics Ltd., Leatherhead, UK) at 20 °C with a cuvette of 1 mm path length (106-QS, Hellma Analytics, Mullheim, Germany) in the range from 180 and 260 nm at 0.5 nm intervals with a time-to-point of 0.5 s. Each peptide sample was prepared to a concentration of 50 µM in the respective solvents. Each spectrum was prepared from an average of five scans obtained with a 1 nm optical bandwidth. The baseline scans were collected with the solvent alone, averaged, and then subtracted from the sample scans. Raw data was exported to excel for processing and expressed as mean residue molar ellipticities [θ] in (deg.cm^2/dmol) and calculated as $\theta = S / (10 \times c \times L \times n)$, where S is the raw CD signal in millidegrees, c is the peptide concentration (M), L is the cuvette path length (cm), and n is the number of peptide bonds.

6.1 CD Spectra of peptides 1, 3 – 6

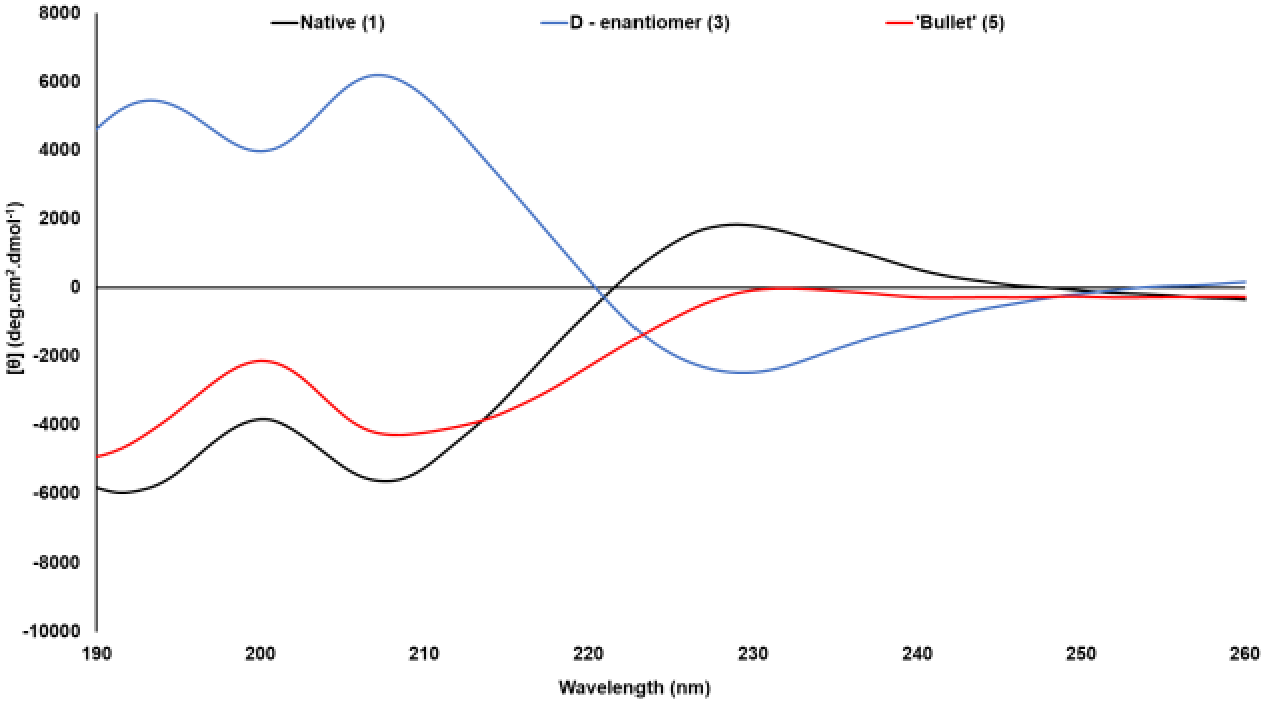

Supplementary Figure S33. CD spectra overlay of capitellacin (**1**) and analogues (**3** and **5**) at 50 µM in 200 µM sodium phosphate buffer (pH ~7.4, 20 °C).

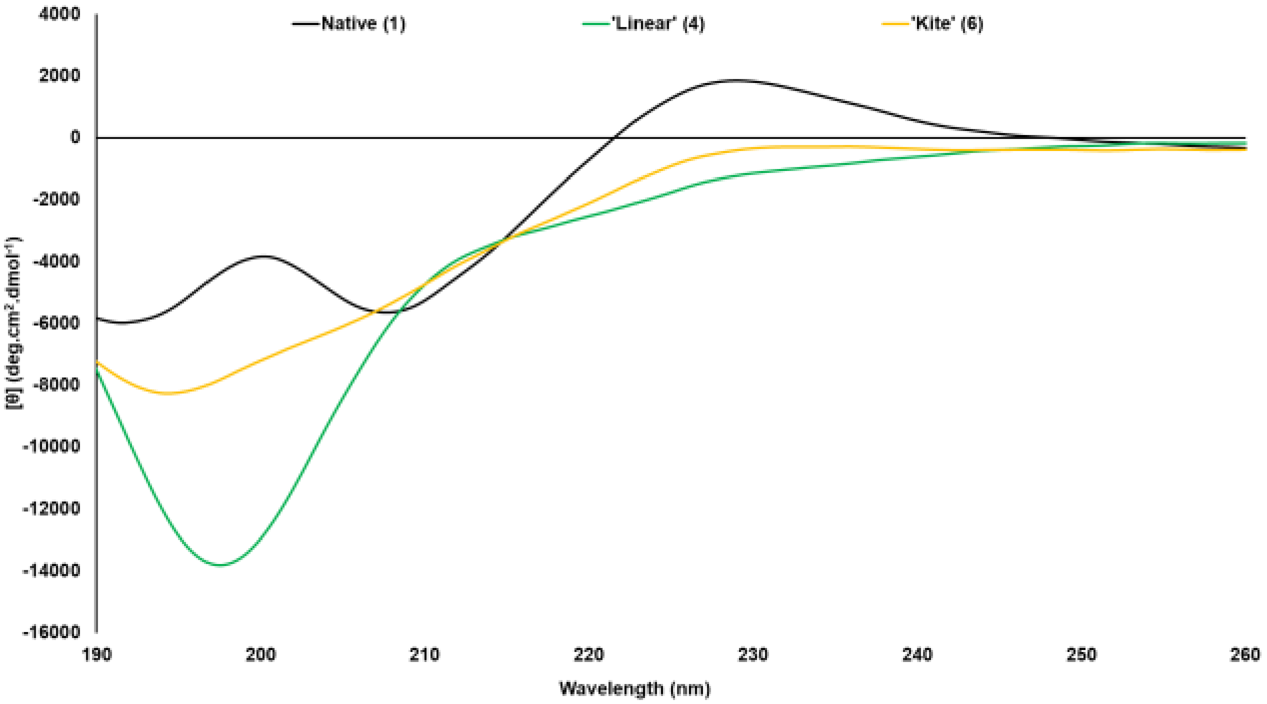

Supplementary Figure S34. CD spectra overlay of capitellacin (**1**) and analogues (**4** and **6**) at 50 µM in 200 µM sodium phosphate buffer (pH ~7.4, 20 °C).

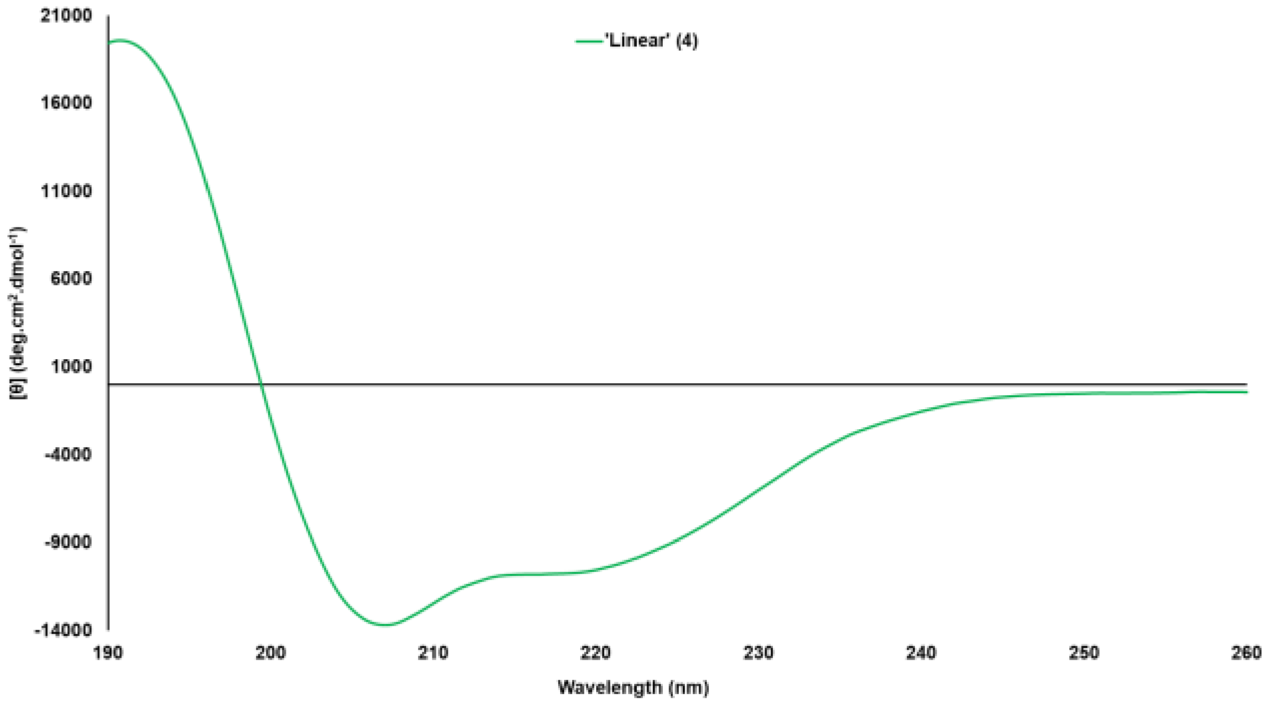

Supplementary Figure S35. CD spectrum of analogue (**6**) at 50 µM in TFE:200 µM sodium phosphate buffer 1:1 (pH ~7.4, 20 °C).

7 Further Supplementary Material

7.1 Additional ¹H NMR, RP-HPLC Traces and ESI-MS

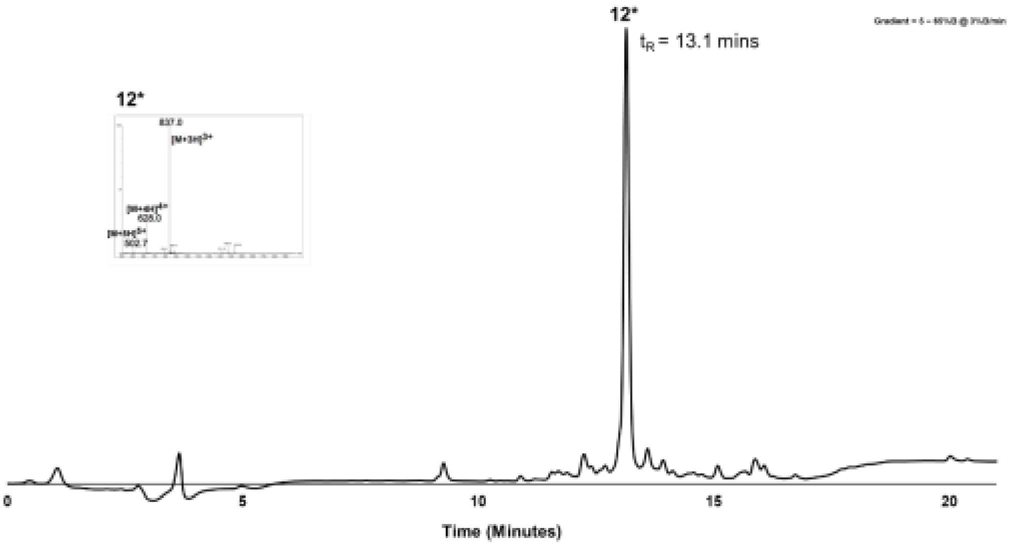

Supplementary Figure S36. **Analytical RP-HPLC** chromatogram (214 nm) of crude product mixture upon mini cleavage of resin **12**. Phenomenex Luna C18 column (100Å, 5 µm, 4.6 mm x 250 mm), linear gradient of 5%B to 65%B over 20 min (ca. 3%/min) at a flow rate of 1 mL/min. ***** denotes the resin cleaved product of the respective peptidyl resin. ESI-MS inset.

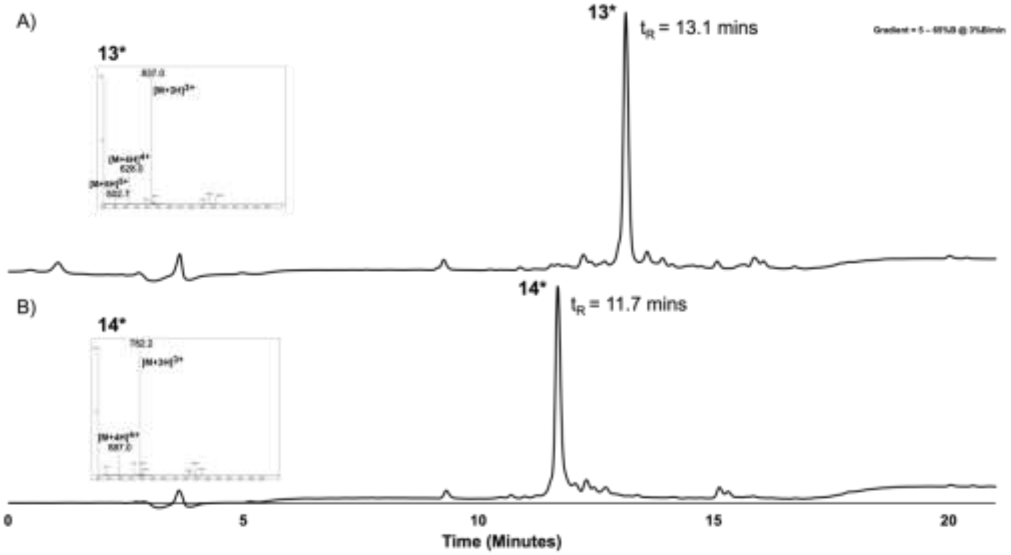

Supplementary Figure S37. **Analytical RP-HPLC** chromatograms (214 nm) of crude product mixture upon mini cleavage of respective peptidyl resins; A) **13** and B) **14**. Phenomenex Luna C18 column (100Å, 5 µm, 4.6 mm x 250 mm), linear gradient of 5%B to 65%B over 20 min (ca. 3%/min) at a flow rate of 1 mL/min. * denotes the resin cleaved product of the respective peptidyl resin. ESI-MS inset.

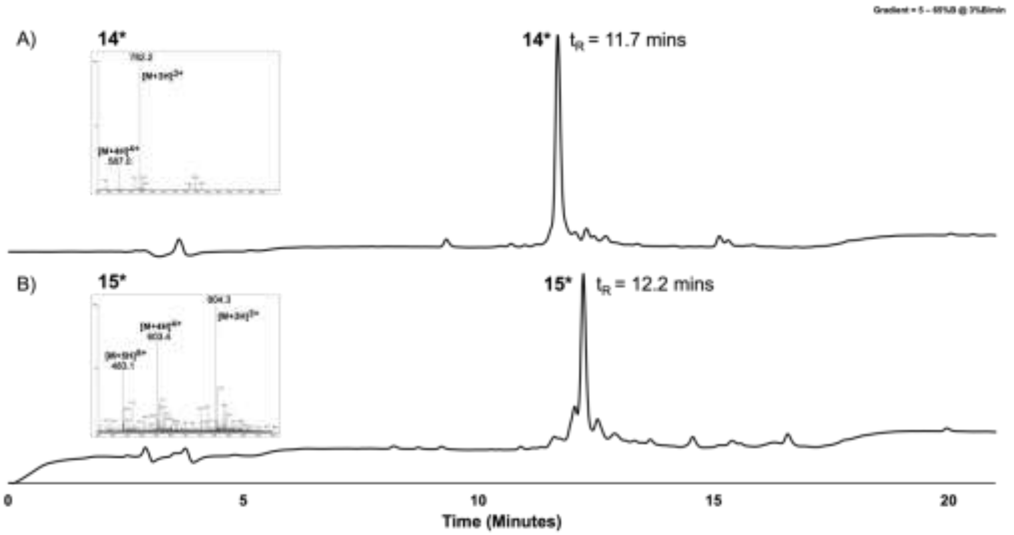

Supplementary Figure S38. **Analytical RP-HPLC** chromatograms (214 nm) of crude product mixture upon mini cleavage of resin; A) **14** and B) **15**. Phenomenex Luna C18 column (100Å, 5 µm, 4.6 mm x 250 mm), linear gradient of 5%B to 65%B over 20 min (ca. 3%/min) at a flow rate of 1 mL/min. * denotes the resin cleaved product of the respective peptidyl resin. ESI-MS inset.

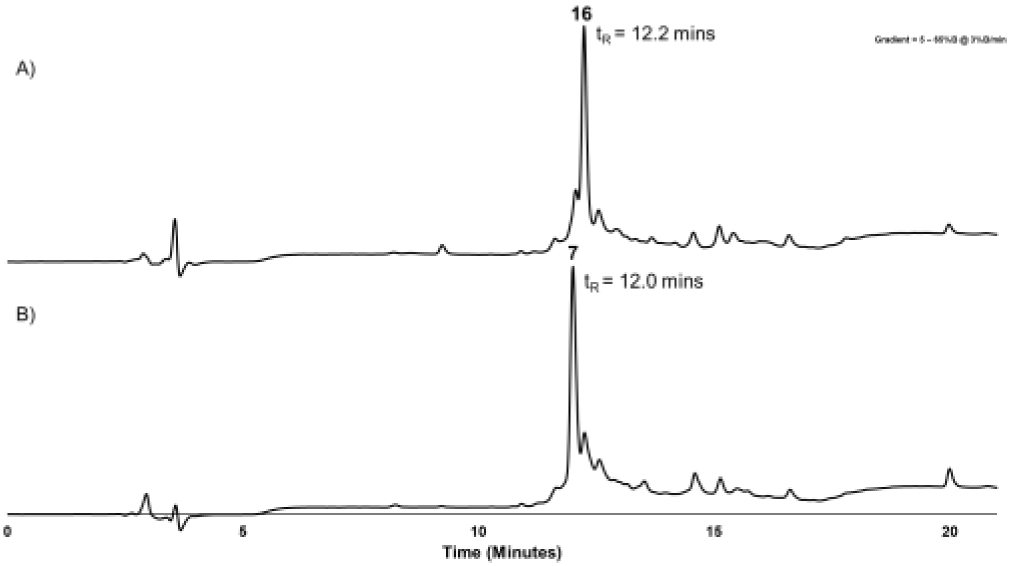

Supplementary Figure S39. **Analytical RP-HPLC** chromatograms (214 nm) during the cyclisation of analogue **7**. A) Peptide **16** prior to treatment with phosphate buffer (10 mM, pH 7.44) and B) Peptide **7** following treatment with phosphate buffer (10 mM, pH 7.44, r.t.). Phenomenex Luna C18 column (100Å, 5 µm, 4.6 mm x 250 mm), linear gradient of 5%B to 65%B over 20 min (ca. 3%/min) at a flow rate of 1 mL/min.

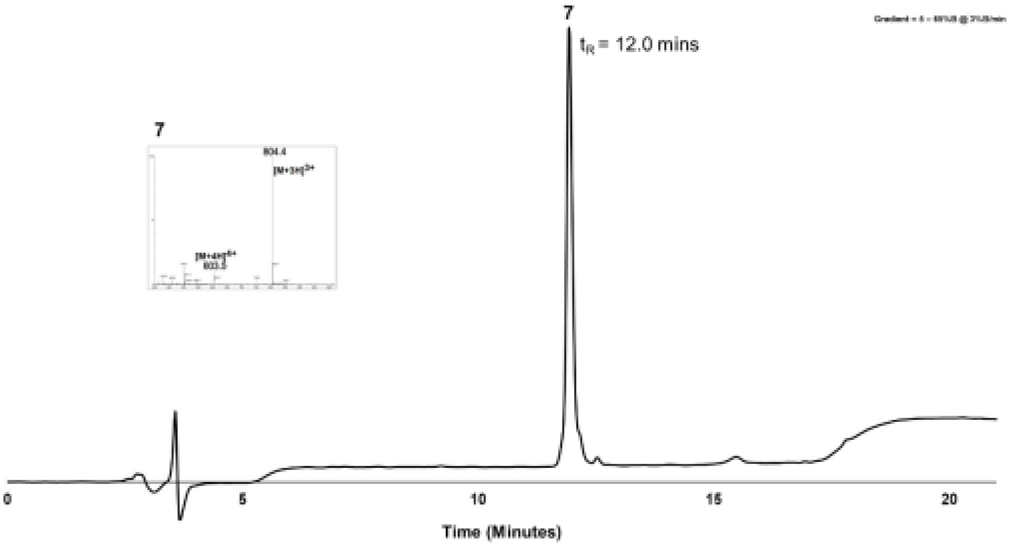

Supplementary Figure S40. Analytical RP-HPLC chromatogram (214 nm) of purified peptide **7** prepared at r.t. Phenomenex Luna C18 column (100Å, 5 µm, 4.6 mm x 250 mm), linear gradient of 5%B to 65%B over 20 min (ca. 3%/min) at a flow rate of 1 mL/min. ESI-MS inset.

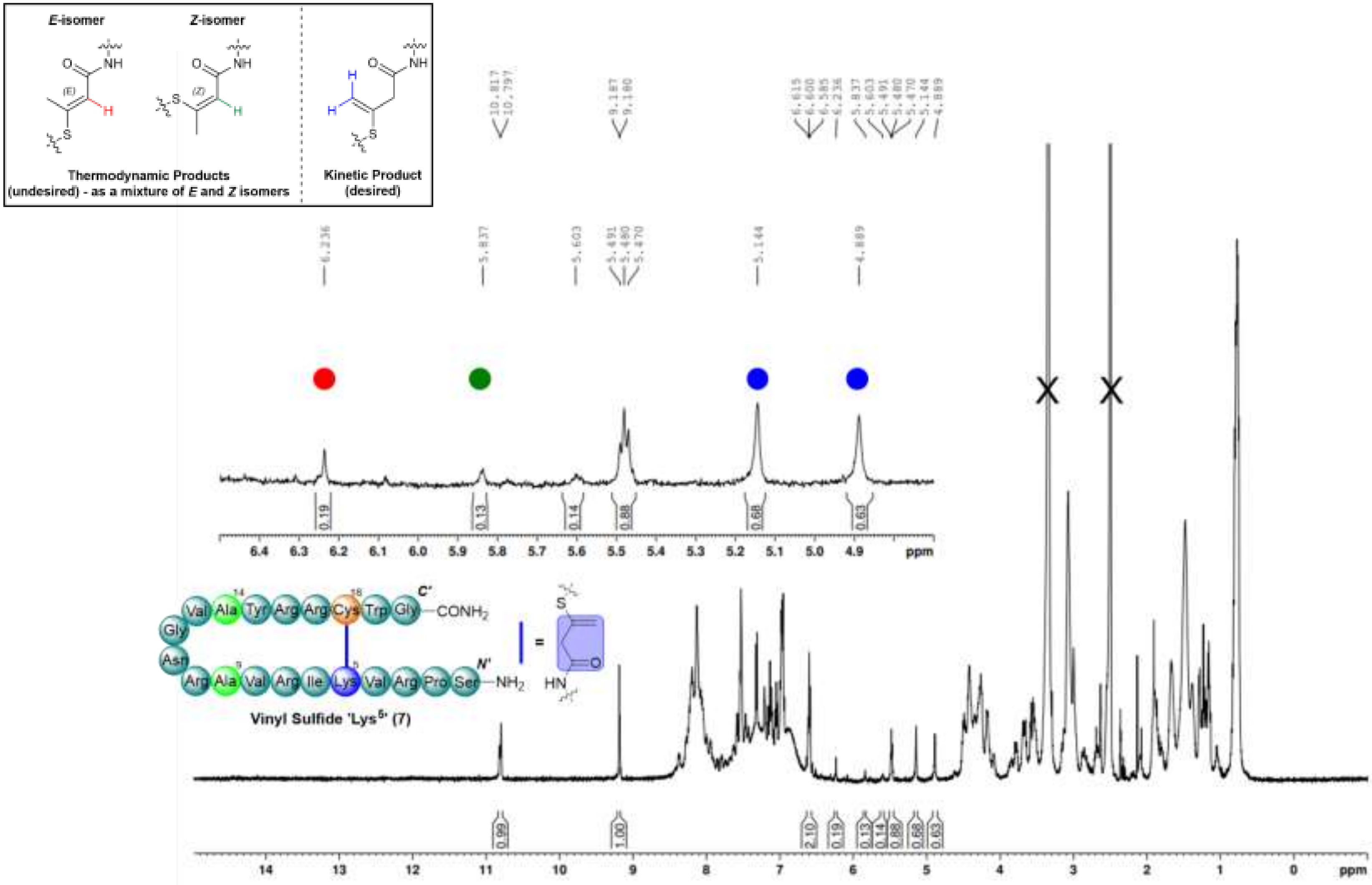

Supplementary Figure S41. ^{1}**H NMR** (DMSO-$_{d6}$) of initial purified analogue **7** prepared at r.t., vinyl proton region inset. X's denote H_2O (δ 3.33 ppm) and DMSO (δ 2.50 ppm) residual peaks The coloured circles denote the proposed *E*- and *Z*-isomers shown in inset box. Cys residues (orange), Ala residues (bright green) and substituted Cys residue and vinyl sulphide bridge (blue).

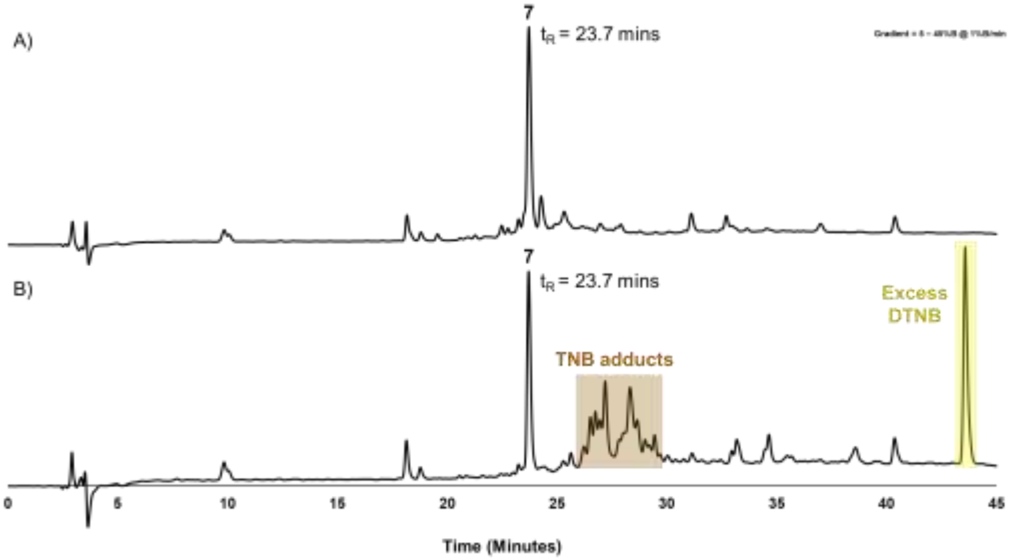

Supplementary Figure S42. **Analytical RP-HPLC** chromatograms (214 nm) of analogue **7**, with quenching of reaction by addition of DTNB. A) Peptide **7** following treatment with phosphate buffer (10 mM, pH 7.44, 4 °C) and B) Peptide **7** treated with DTNB following treatment with phosphate buffer (10 mM, pH 7.44). TNB adducts in gold, and excess DTNB in yellow. Phenomenex Luna C18 column (100Å, 5 µm, 4.6 mm x 250 mm), linear gradient of 5%B to 45%B over 40 min (ca. 1%/min) at a flow rate of 1 mL/min.

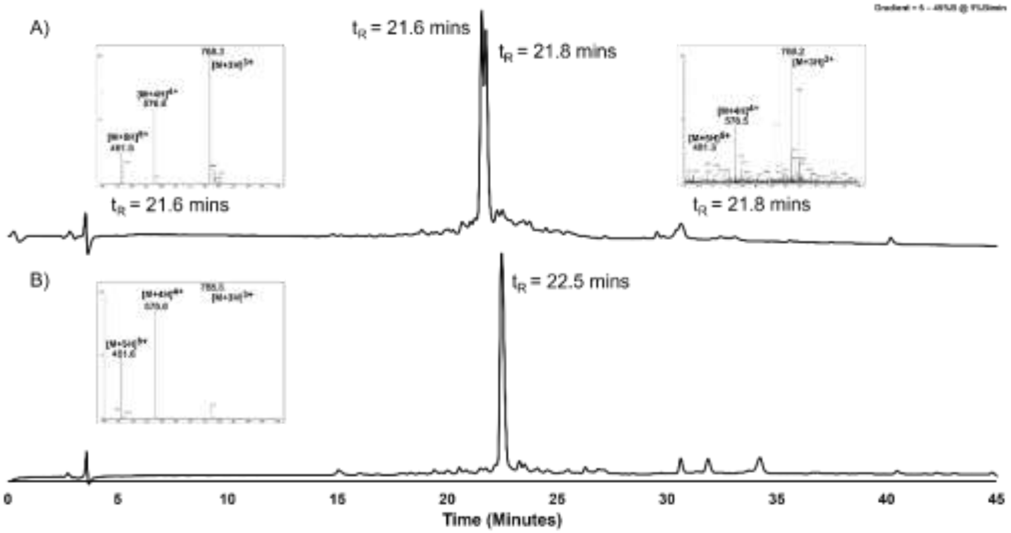

Supplementary Figure S43. **Analytical RP-HPLC** chromatograms (214 nm) of peptides liberated from a small portion of the resin following orthogonal deprotection of respective Dap sidechain (Dde/Alloc) during the syntheses of analogue **10**. A) Under initial protocol employing Dap(N^{β}-Dde) and B) under revised protocol employing Fmoc-Dap(N^{β}-Alloc)-OH. Phenomenex Luna C18 column (100Å, 5 µm, 4.6 mm x 250 mm), linear gradient of 5%B to 45%B over 40 min (ca. 1%/min) at a flow rate of 1 mL/min.. ESI-MS inset of trace A (left) mass calculated for [$C_{99}H_{164}N_{39}O_{24}S_1$] 2303.3; deconvoluted observed: 2302.3 ± 0.3. Charge states; 461.5 $[M+5H]^{5+}$, 576.6 $[M+4H]^{4+}$, 768.3 $[M+3]^{3+}$; (right) mass calculated for [$C_{99}H_{164}N_{39}O_{24}S_1$] 2303.3; deconvoluted observed: 2301.7 ± 0.3. Charge states; 461.3 $[M+5H]^{5+}$, 576.5 $[M+4H]^{4+}$, 768.2 $[M+3]^{3+}$; ESI-MS inset of trace B; mass calculated for [$C_{99}H_{164}N_{39}O_{24}S_1$] 2303.3; deconvoluted observed: 2302.4 ± 0.6. Charge states; 461.6 $[M+5H]^{5+}$, 576.6 $[M+4H]^{4+}$, 768.3 $[M+3]^{3+}$.

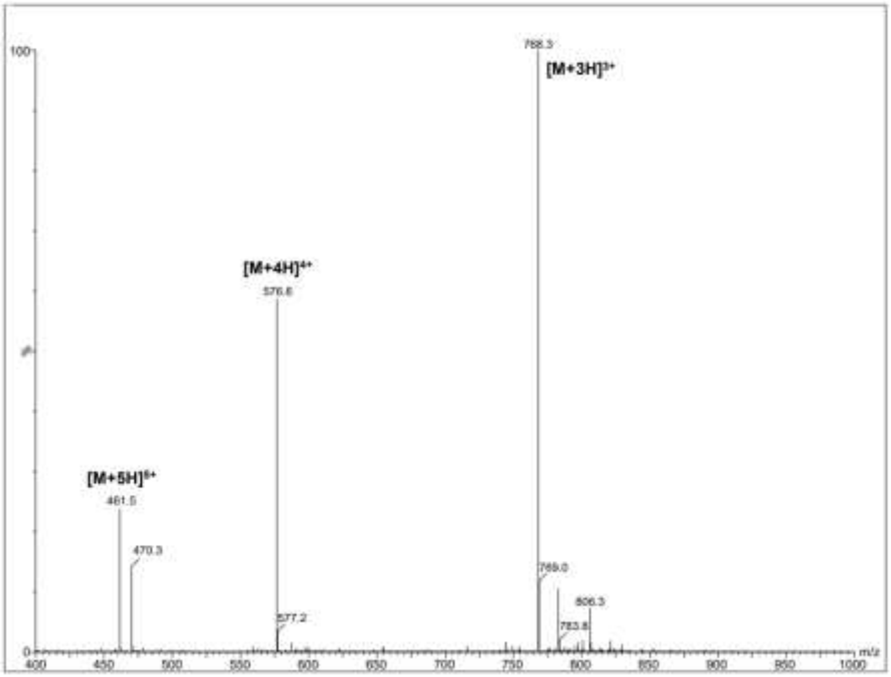

Supplementary Figure S44. **ESI-MS** of peak t_R = 21.6 min in Supplementary Figure 7.8A, mass calculated for [$C_{99}H_{164}N_{39}O_{24}S_1$] 2303.3; deconvoluted observed: 2302.3 ± 0.3. Charge states; 461.5 $[M+5H]^{5+}$, 576.6 $[M+4H]^{4+}$, 768.3 $[M+3]^{3+}$.

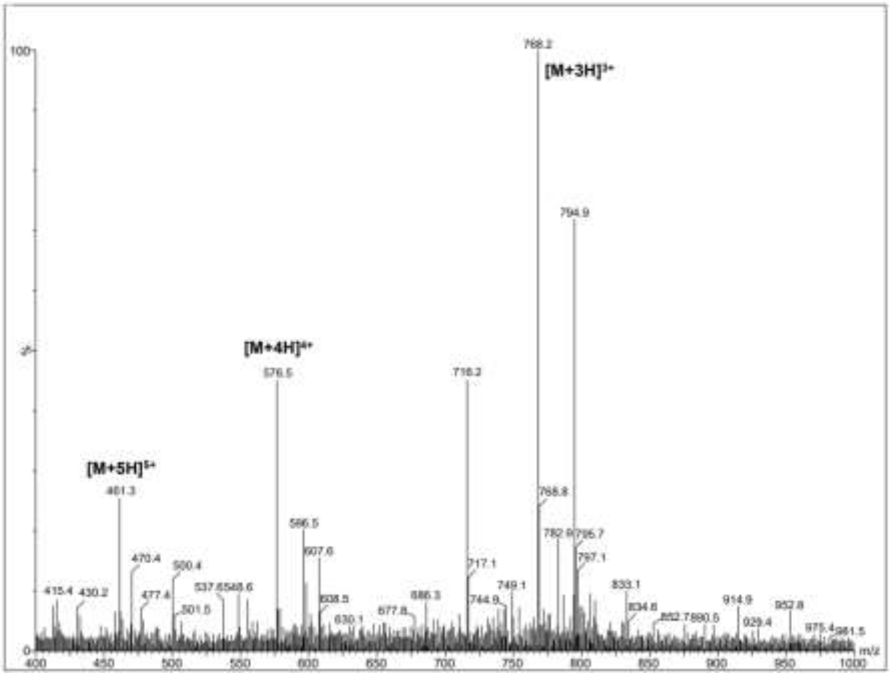

Supplementary Figure S45. **ESI-MS** of peak t_R = 21.8 min in Supplementary Figure 7.8A, mass calculated for [$C_{99}H_{164}N_{39}O_{24}S_1$] 2303.3; deconvoluted observed: 2301.7 ± 0.3. Charge states; 461.3 $[M+5H]^{5+}$, 576.5 $[M+4H]^{4+}$, 768.2 $[M+3]^{3+}$.

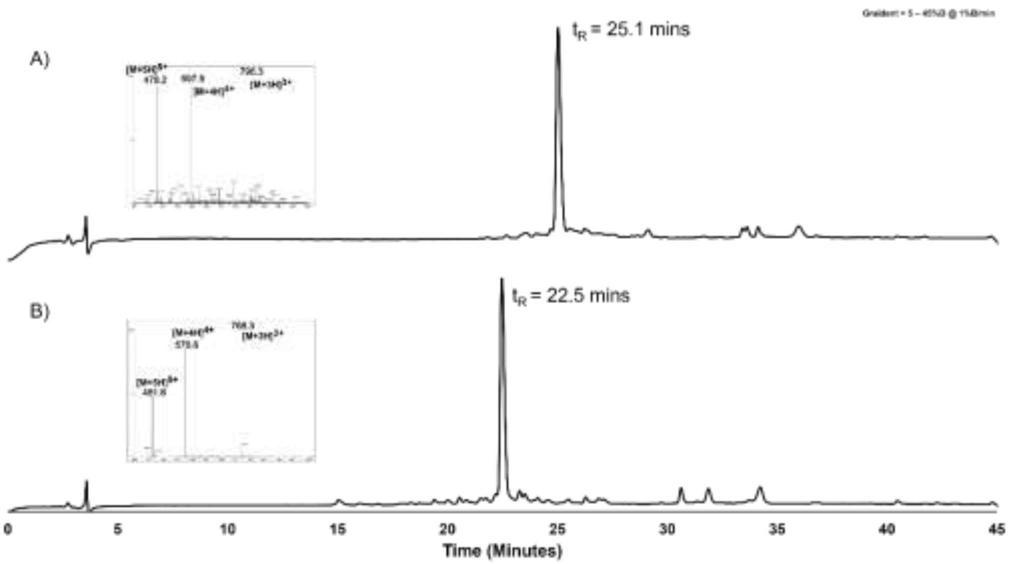

Supplementary Figure S46. **Analytical RP-HPLC** chromatograms (214 nm) during the revised synthesis of analogue **10**. A) Following synthesis by flow-SPPS and B) following Dap(N^{β}-Alloc) deprotection by $Pd(PPh_3)_4$ and $PhSiH_3$. Phenomenex Luna C18 column (100Å, 5 µm, 4.6 mm x 250 mm), linear gradient of 5%B to 45%B over 40 min (ca. 1%/min) at a flow rate of 1 mL/min. ESI-MS inset of trace A; mass calculated for [$C_{103}H_{168}N_{38}O_{26}S_1$] 2386.3; deconvoluted observed: 2386.0 ± 0.1. Charge states; 478.2 $[M+5H]^{5+}$, 597.5 $[M+4H]^{4+}$, 796.3 $[M+3]^{3+}$; ESI-MS inset of trace B; mass calculated for [$C_{99}H_{164}N_{39}O_{24}S_1$] 2303.3; deconvoluted observed: 2302.4 ± 0.6. Charge states; 461.6 $[M+5H]^{5+}$, 576.6 $[M+4H]^{4+}$, 768.3 $[M+3]^{3+}$.

7.2 Data Summary of Synthetic Peptides

Supplementary Table S1. Synthetic data summary of capitellacin disulphide analogues (**1**, **3 – 6**).

Peptide (#)	t_R (min)[a]	*m/z* calculated	*m/z* observed (deconvoluted)	Purity (> %)	% Yield[b]
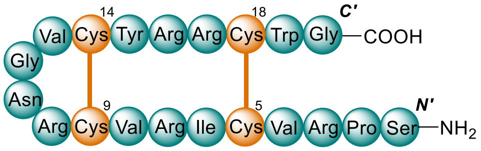 **1**	26.7	2379.1	2379.2 ± 0.2	97	10.0
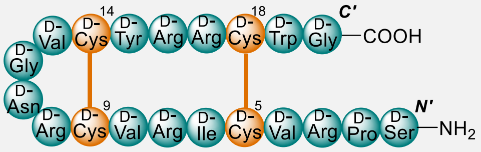 **3**	26.7	2379.1	2379.1 ± 0.2	98	0.9
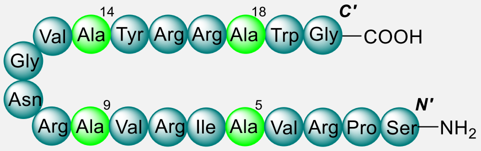 **4**	23.2	2255.2	2255.7 ± 0.3	97	5.3
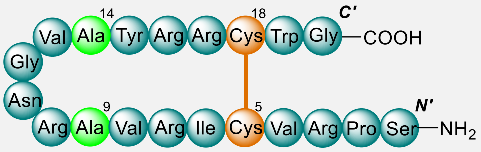 **5**	25.5	2317.2	2317.1 ± 0.2	98	7.2
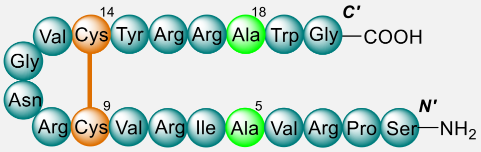 **6**	25.0	2317.2	2317.2 ± 0.1	98	3.7

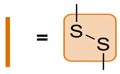

[a]Phenomenex Luna C18 (100Å, 5 µm, 4.6 mm x 250 mm), linear gradient 5%B to 45%B over 40 min (*ca.* 1%B/min) at 1 mL/min.

[b]% Yield as overall yield determined by initial resin loading.

Supplementary Table S2. Comparison of simple peptide structure, retention time and MIC against *E. coli* for capitellacin (**1**) and disulphide analogues (**3 – 6**).

**Peptide (#)**	**t_R (min)[a]**	**MIC against *E. coli* ATCC 25922**
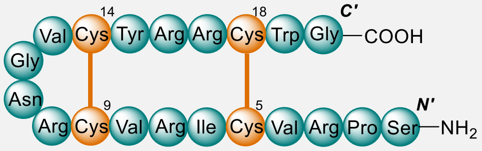 **1**	**26.7**	**1**
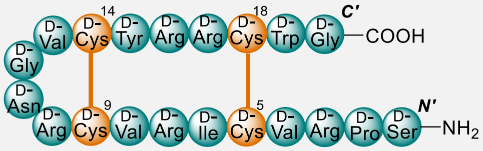 **3**	**26.7**	**4***
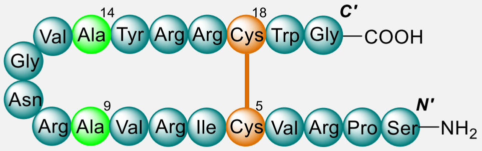 **5**	**25.5**	**1**
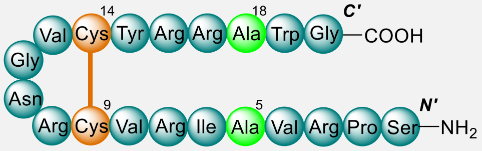 **6**	**25.0**	**4**
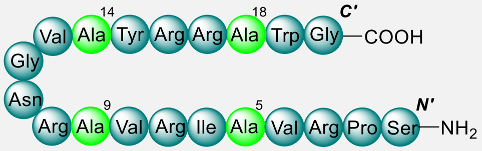 **4**	**23.2**	**> 64**

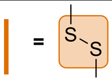

[a]Phenomenex Luna C18 (100Å, 5 µm, 4.6 mm x 250 mm), linear gradient 5%B to 45%B over 40 min (*ca.* 1%B/min) at 1 mL/min.

*** = Outlier of D-enantiomer, potentially as acts via a different mechanism, one with a chiral target [7].**

Supplementary Table S3. Synthetic data summary of vinyl sulphide capitellacin analogues (**7 – 11**).

**Peptide (#)**	**t_R (min)[a]**	**m/z calculated**	**m/z observed (deconvoluted)**	**Purity (> %)**	**% Yield[b]**
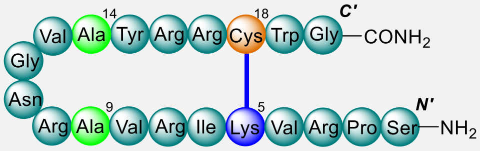 **7**	23.8	2409.3	2410.1 ± 0.1	98	4.1
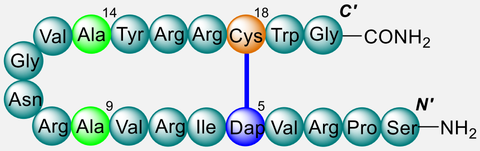 **8**	24.3	2367.3	2368.1 ± 0.1	98	2.0
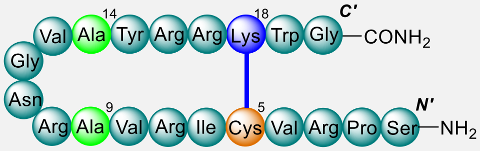 **9**	24.8	2409.3	2410.7 ± 0.3	95	1.9
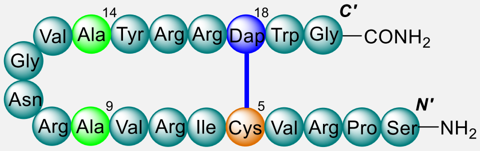 **10**	24.4	2367.3	2368.0 ± 0.3	95	3.5
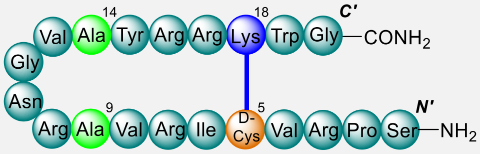 **11**	24.5	2409.3	2409.7 ± 0.2	96	2.2

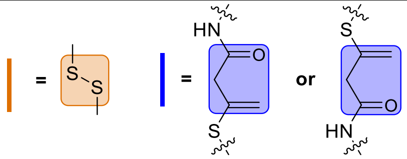

[a]Phenomenex Luna C18 (100Å, 5 µm, 4.6 mm x 250 mm), linear gradient 5%B to 45%B over 40 min (*ca.* 1%B/min) at 1 mL/min.

[b]% Yield as overall yield determined by initial resin loading.

Supplementary Table S4. Comparison of simple peptide structure, retention time and MIC against *E. coli* for 'bullet' capitellacin (**5**) and vinyl sulphide analogues (**7 - 11**).

Peptide (#)	t_R (min)[a]	MIC against *E. coli* ATCC 25922
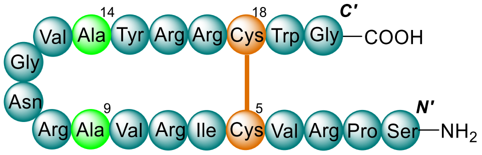 **5**	25.5	1
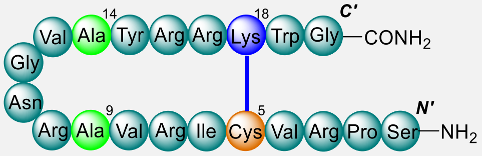 **7**	24.8	4
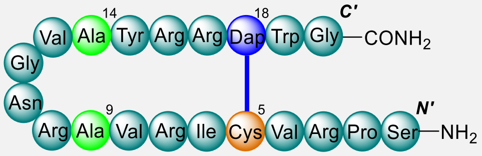 **8**	24.4	8
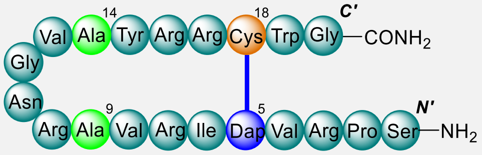 **9**	24.3	8
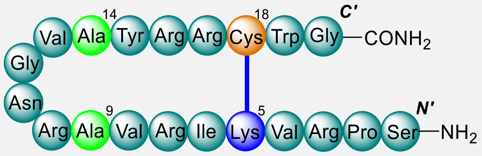 **10**	23.8	8
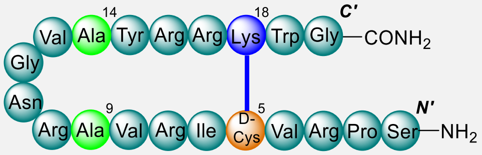 **11**	24.5*	> 64*

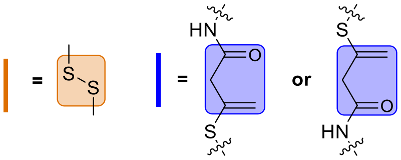

[a]Phenomenex Luna C18 (100Å, 5 µm, 4.6 mm x 250 mm), linear gradient 5%B to 45%B over 40 min (*ca.* 1%B/min) at 1 mL/min.

*** = Outlier as has inverted stereochemistry at residue 5.**

7.3 Abbreviation Structures

Trt =

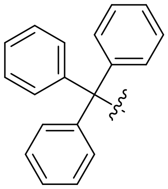

Boc =

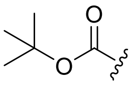

Pbf =

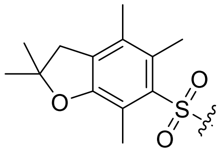

Fmoc =

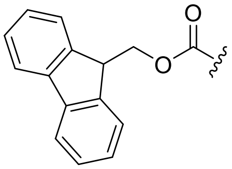

***t*Bu =**

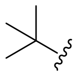

Alloc =

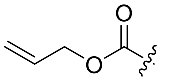

Dde =

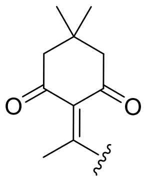

Acm =

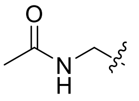

HATU =

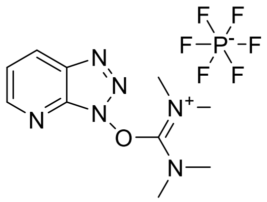

DIPEA =

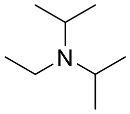

***sym*-collidine =**

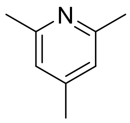

PyAOP =

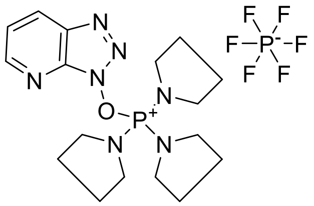

EEDQ =

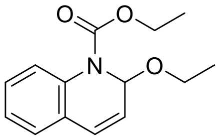

Rink Amide =

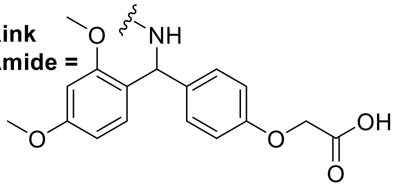

Supplementary Figure S47. Structures of protecting groups for peptidyl side chains and N^{α}-amino group, resin linkers and commonly employed amide bond forming reagents from syntheses performed within this work.

8 Flow Chemistry Setup

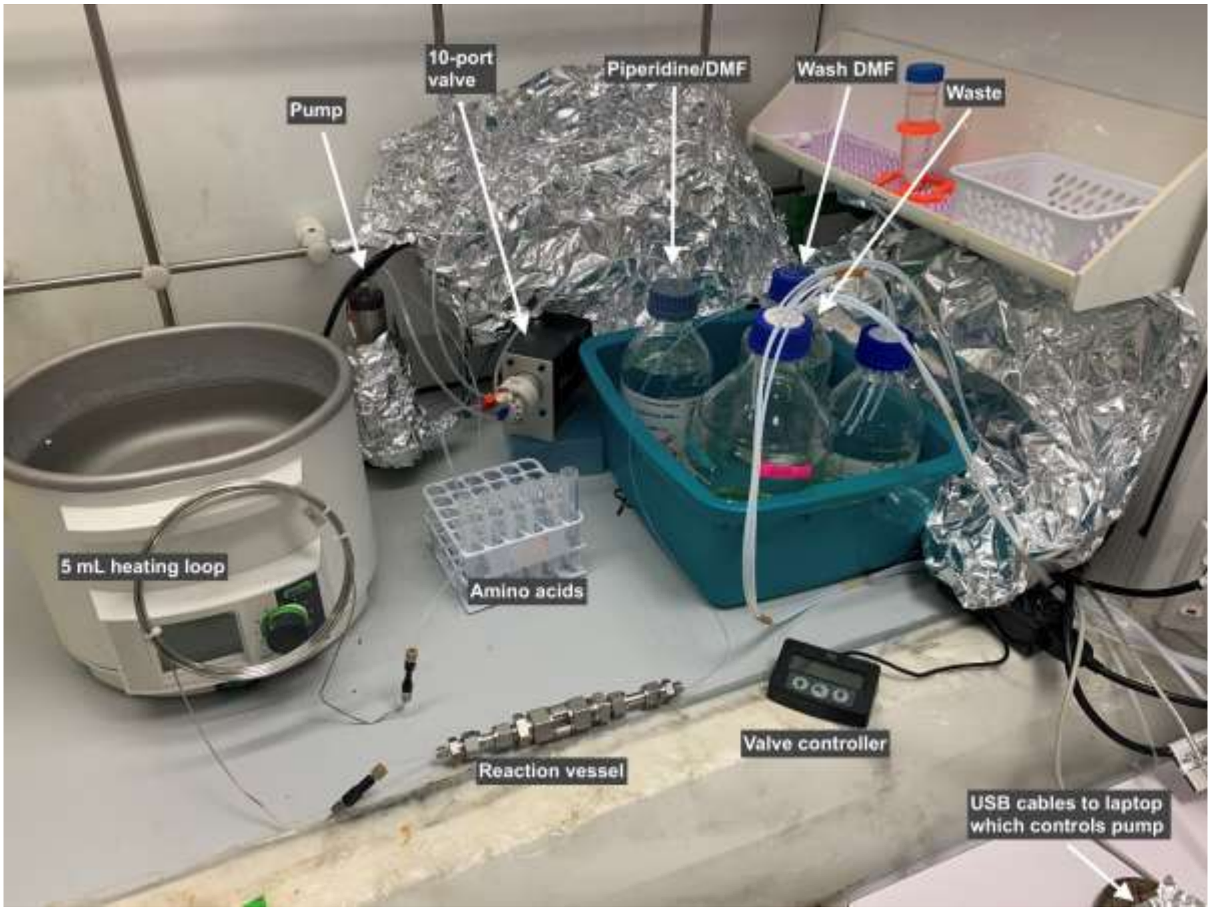

Supplementary Figure S48. Flow setup employed for the synthesis of peptides (**1**, **3 – 11**). System contained within a fume hood and key components labelled.

9 References

1. Simon, M.D.; Heider, P.L.; Adamo, A.; Vinogradov, A.A.; Mong, S.K.; Li, X.; Berger, T.; Policarpo, R.L.; Zhang, C.; Zou, Y.; et al. Rapid Flow-Based Peptide Synthesis. *ChemBioChem* **2014**, *15*, 713–720, doi:https://doi.org/10.1002/cbic.201300796.

2. Abdel Monaim, S.A.H.; Ramchuran, E.J.; El-Faham, A.; Albericio, F.; de la Torre, B.G. Converting Teixobactin into a Cationic Antimicrobial Peptide (AMP). *J. Med. Chem.* **2017**, *60*, 7476–7482, doi:10.1021/acs.jmedchem.7b00834.

3. Brimble, M.A.; Cameron, A.J.; Harris, P.W. On-Resin Preparation of Allenamidyl Peptides: A Versatile Chemoselective Conjugation and Intramolecular Cyclisation Tool. *Angew. Chem. Int. Ed.* **2020**.

4. Clinical and Laboratory Standards Institute. Performance standards for antimicrobial susceptibility testing; sixteenth informational supplement. CLSI document M100-S16, Wayne, Pa. **2006**.

5. National Committee for Clinical Laboratory Standards**.** Reference method for broth dilution antifungal susceptibility testing of yeasts. Approved standard-second edition M27-A2, Vol. 22 No. 15. National Committee for Clinical Laboratory Standards, Wayne, Pa. **2002**.

6. Panteleev, P.V.; Tsarev, A.V.; Safronova, V.N.; Reznikova, O.V.; Bolosov, I.A.; Sychev, S.V.; Shenkarev, Z.O.; Ovchinnikova, T.V. Structure Elucidation and Functional Studies of a Novel β-Hairpin Antimicrobial Peptide from the Marine Polychaeta Capitella Teleta. *Mar. Drugs* **2020**, *18*, 620, doi:10.3390/md18120620.

7. Henriques, S.T.; Peacock, H.; Benfield, A.H.; Wang, C.K.; Craik, D.J. Is the Mirror Image a True Reflection? Intrinsic Membrane Chirality Modulates Peptide Binding. *J. Am. Chem. Soc.* **2019**, *141*, 20460–20469, doi:10.1021/jacs.9b11194.
